# The impact and outcomes of cancer-macrophage fusion

**DOI:** 10.1186/s12885-023-10961-9

**Published:** 2023-06-01

**Authors:** Mengtao Li, John R. Basile, Sanjay Mallya, Yi-Ling Lin

**Affiliations:** 1 Division of Diagnostic and Surgical Sciences, School of Dentistry, University of California, CHS 23-068B. 10833 Le Conte Ave, Los Angeles, CA 90095 USA; 2 Department of Oncology and Diagnostic Sciences, University of Maryland Dental School, Baltimore, MD USA; 3 Gene regulation program, Jonsson Comprehensive Cancer Center, University of California, Los Angeles, CA USA; 4 Department of Oncology and Diagnostic Sciences, University of Maryland Dental School, 650 W. Baltimore St, Baltimore, MD 7261, 21201 USA

**Keywords:** Cancer, Cell fusion, Macrophages, Myeloid cells, Stromal cells, Tumorigenicity

## Abstract

**Background:**

Cancer’s hallmark feature is its ability to evolve, leading to metastasis and recurrence. Although genetic mutations and epigenetic changes have been implicated, they don’t fully explain the leukocytic traits that many cancers develop. Cell fusion between cancer and somatic cells, particularly macrophages, has been suggested as an alternative pathway for cancer cells to obtain new traits by acquiring exogenous genetic material.

**Methods:**

This study aims to investigate the potential biological outcomes of tumor-myeloid cell fusion by generating tumor-macrophage hybrid cells. Two clones with markedly different tumorigenicity were selected, and RNA-seq was used to compare their RNA expressions with that of the control cells. Based on the results that the hybrid cells showed differential activation in several upstream regulator pathways that impact their biological behaviors, the hybrid cells’ abilities to recruit stromal cells and establish angiogenesis as well as their cell cycle distributions were investigated through in vitro and in vivo studies.

**Results:**

Although both hybrid clones demonstrated p53 activation and reduced growth rates, they exhibited distinct cell cycle distributions and ability to grow in vivo. Notably, while one clone was highly tumorigenic, the other showed little tumorigenicity. Despite these differences, both hybrid clones were potent environmental modifiers, exhibiting significant abilities to recruit stromal and immune cells and establish angiogenesis.

**Conclusions:**

The study revealed that tumor-somatic cell fusion is a potent environmental modifier that can modulate tumor survival and evolution, despite its relatively low occurrence. These findings suggest that tumor-somatic cell fusion could be a promising target for developing new cancer therapies. Furthermore, this study provides an experimental animal platform to investigate cancer-myeloid fusion and highlights the potential role of tumor-somatic cell fusion in modulating the tumor environment.

**Supplementary Information:**

The online version contains supplementary material available at 10.1186/s12885-023-10961-9.

## Introduction

Cancer progression requires that the tumor cells obtain new capabilities, often through mutations, that allow them to evade immune defenses and treatment. Natural mutations due to environmental stress have been considered the main driving force for tumor heterogeneity. However, this process takes time and does not fully explain the speedy tumor evolution commonly associated with recurrence and metastasis [1]. It also cannot explain the observation that many cancer cells express leukocytic traits. Unlike mutations caused by environmental stress, cancer-somatic cell fusion allows the tumor cells to acquire exogenous genetic material through a non-mutational mechanism capable of generating diversity in the cancer genome within a relatively short time.

Although the ability to fuse with cancer cells is not limited to bone marrow-derived cells [2–4], myeloid cells, namely macrophages, are regarded as the most common fusion partner [5]. Most research shows that cancer-somatic cell fusion provides an opportunity to acquire novel properties to facilitate tumor progression, particularly when the fusion partners are myeloid cells [4, 6–10]. Nonetheless, the induction of anti-tumor events has been reported, mostly when cancer cells fuse with mesenchymal cells or stem cells [3, 11].

As many cancers exhibit leukocytic traits, it is postulated that tumor-leukocytic cell fusion may be an important mechanism for cancer plasticity and metastasis [1, 12–15]. In support of this notion, tumor-leukocyte hybrids have been demonstrated in animal studies and confirmed in patient specimens [16, 17]. Clinical studies further show that cancer-leukocyte hybrids potentiate tumor heterogeneity and impact patient prognosis [15, 18].

Cell-cell fusion is a physiological process required for the embryonic development and formation of skeletal muscle, osteoclasts, and syncytiotrophoblasts [19]. In adult tissue, heterotypic cell-cell fusion is rare, though it can be detected during tissue repair and chronic inflammation [20, 21]. Chemotherapy and radiation, two major treatment modalities for cancer therapy, have been shown to promote this otherwise uncommon phenomenon [22, 23]. By studying biopsies from patients receiving allogeneic stem cell transplants that later developed solid tumor metastases, Pawelek’s group used forensic genetics to document a high percentage of the metastatic tumor cells carrying donor and patient DNA sequences, indicating that hybrids were present and likely the source of metastasis [17]. This finding suggests that tumor-macrophage fusion may not be as rare as once thought in specific clinical scenarios and underscores the potential role of tumor-macrophage hybrids in recurrence and metastasis, particularly after cancer treatment.

Despite much research on cell-cell fusion, few studies have provided an experimental system that followed the fusion products from generation through their evolution. In this study, we examined cloned cancer-macrophage hybrids through in vitro and in vivo analysis. They were injected into the syngeneic mice to demonstrate their individual tumorigenicity and ability to impact the tumor microenvironment. RNA-seq-based transcriptome analysis revealed potential mechanisms associated with phenotypes of tested hybrid clones. The hybrid cells demonstrated varying levels of tumor-forming ability, ranging from no or minimal tumor formation to high tumorgenicity, and had a substantial capacity to manipulate the tumor microenvironment. Our results showed that tumor-myeloid hybrid cells are potent environmental modifiers and have important biological implications for cancer survival and evolution.

## Materials and methods

### Animal use

C3H/HeNCrl (C3H) mice were obtained from Charles River (Hollister, CA, USA), and NOD-scid IL2Rgammanull (NSG) mice were obtained from Radiation Oncology, UCLA (originally from Jackson Laboratory, Bar Harbor, ME, USA). Mice of both sexes, aged 2–4 months, were used. Mice were maintained under the care of the UCLA Division of Laboratory Animal Medicine. During the study period, trained laboratory staff conducted daily health and wellness check on the mice, and all efforts were made to minimize suffering.

### Tumor production, tissue harvest and processing

Tumor injection was performed under isoflurane anesthesia. For tumor formation, the mice received a subcutaneous injection of SCCVII/SF-derived cells (10^6^) suspended in PBS (50 µL) into the dorsal flank. Twenty-four hours before euthanasia, the mice received an intraperitoneal injection of BrdU (100 µL 10 mg/mL) to label the replicating cells. Tumor-bearing mice were euthanized on post-injection day 16 (NSG mice) or 21 (C3H mice) by CO2 inhalation followed by cervical dislocation. The injection site tissue (with or without tumor formation) was excised, weighed, and processed for the subsequent studies.

For histological analysis, the harvested tumors were fixed in formalin (10%) and paraffin-embedded to form tissue blocks. Tissue sections were cut, dewaxed and rehydrated before staining. For flow cytometry, the excised tumor was placed in serum-free RPMI-1640 (4 ml) and cut into small pieces. Final concentrations of liberase DL (0.28U/ml, Roche), liberase TL (0.28U/ml, Roche), and DNase I (80 ug/ml) were added to digest the tissue in a 37 °C shaking water bath. After 45 min, the tissue suspension was pipetted multiple times through a 5 ml pipette to break up the cellular clumps. PBS (10 ml) containing FCS (2%) was added to stop the digestion, and the suspension was passed through a 100 μm cell strainer to generate a single-cell suspension. The cells were then spun down, resuspended in RPMI containing FCS (10%) and DMSO (10%), aliquoted, and stored in liquid nitrogen. Before usage, the cells were thawed and resuspended in PBS with FCS (2%).

### Plasmid construction and retrovirus production

pMSCVpuro, pMSCVneo and pLEGFP-N1 plasmids were purchased from Takara Bio (Mountain View, CA, USA). pMSCVpuro-EGFP was constructed by subcloning the EGFP-N1 sequence from pEGFP-N1 plasmid (XhoI and NotI/Klenow) into pMSCVpuro (XhoI and HpaI). For pLEGFP-mH2B construction, primers 5’-ATACTCGAGATGCCTGAGCCTGCGAAG-3’ and 5’-CGCGGATCCTTCTGGTCTTTTGAATC-3’ were used to amplify the mouse histone H2B sequence. The PCR product was digested with XhoI and BamHI before insertion into the compatible cloning site in pLEGFPN1. This plasmid expresses a histone H2B-GFP protein in the nucleus. For retrovirus infection, supernatant from the retrovirally transfected 293FT cells was used to infect the cells as previously described [24].

### Cell-cell fusion and cloning of hybrid cells

The SCCVII/SF cell line was originally established by Dr. Herman D. Suit at Harvard University and has been widely used in C3H mice as an immunocompetent model for head and neck cancer [25, 26]. This cell line was a gift from Dr. Mae St. John, UCLA Head and Neck Surgery. The cells were cultured in RPMI-1640 media supplemented with FBS (10%) and maintained in a 5% CO_2_ incubator. pLEGFP-mH2B retrovirus was used to transduce SCCVII/SF to generate the SCCVII/SF-H2B-GFP cell line. SCCVII/SF-H2B-GFP was co-cultured with bone marrow macrophages (BMM) in MEM-α media supplemented with FBS (10%), M-CSF (50 ng/mL) and RANKL (50 ng/mL) to support BMM growth and promote cell-cell fusion. Bone marrow was harvested from the long bones of euthanized mice [24].

For cloning hybrid cell lines, pMSCVpuro-EGFP-transduced BMM were co-cultured with SCCVII/SF in MEM-α containing M-CSF and RANKL as described above. BMM and SCCVII/SF mono-cultures were used as the controls. The co-culture was withdrawn from M-CSF and RANKL after five days, and the media was switched to RPMI-1640 containing FBS (10%) and puromycin (5 µg/ml) to enrich the hybrid cells. No viable cells were seen in BMM or SCCVII/SF mono-cultures after one week. Only the SCCVII/SF cells that acquired the puromycin-resistant gene via fusion with the pMSCVpuro-EGFP-transduced BMM could survive in the co-culture. The hybrid cells also acquired the EGFP gene. The cells were further cloned and tested for tumorgenicity in mice.

### In vitro cell proliferation

The cells were plated in quadruplicate onto a 96-well plate (200 cells/well) and allowed to attach overnight. Cell proliferation analysis was carried out daily for four days with a CellTiter-Glo luminescent cell viability assay kit (#G7570, Promega, Madison, WI, USA), following the manufacturer’s instructions. The luminescence values were used to plot against the time for cell growth curves. The cell doubling time was estimated by an online Cell Doubling Time Calculator (https://www.omnicalculator.com/biology/cell-doubling-time).

### Antibodies

Anti-CD8a (#14-0808-80), anti-CD49b/ITGA2 (clone DX5. #14-5971-85), anti-FOXP3 (#14-5773-82), anti-F4/80 (#14-4801-82), anti-BrdU-APC (#17-5071-42), anti-iNOS-APC (#17-5920-82), anti-Arginase 1-APC (17-3697-82), and Alexa Fluor-conjugated secondary antibodies (# A-11011) were purchased from Thermo Fisher Scientific (Waltham, MA, USA). Anti-CD4-APC (#100412), anti-CD25-PerCP/Cy5.5 (#101911), anti-CD45-APC (#103112), anti-CD8-APC (#100712), anti-F4/80-PerCP/Cy5.5 (#123128), anti-CD11b-PE (#101208), anti-FOXP3-PE (#126404), and anti-CD16/32 (#101302) were purchased from BioLegend (San Diego, CA, USA). Anti-CD31 (#ab28364) was purchased from Abcam (Boston, MA, USA). Anti-PU.1 (#2266) was purchased from Cell Signaling Technology (Danvers, MA, USA).

### Immunofluorescence and immunohistochemistry

For immunofluorescence staining, cultured cells or tissue sections were fixed in paraformaldehyde (2%). Cultured cells were also permeabilized with TritonX-100 (0.1%). Normal goat serum (2.5%) was used for blocking. Cells were stained overnight at 4 °C with a primary antibody, and the signal was then detected by a corresponding Alexa Fluor-conjugated secondary antibody. DAPI was used to stain the nuclei. For bright field staining, the tissue sections were subjected to standard hematoxylin and eosin (H&E) or immunohistochemical (IHC) stains (ImmPRESS reagent, Vector Laboratories, Burlingame, CA, USA). Appropriate positive and negative controls were included for all immunostains.

### RNA-seq

RNA-seq was conducted by the UCLA Technology Center for Genomics and Bioinformatics. Briefly, total RNA was isolated from replicates of cells, followed by mRNA enrichment, reverse transcription to generate cDNA, end repair to generate blunt ends, A-tailing, adaptor ligation, and PCR amplification. Different adaptors were used for multiplexing samples in one lane. Libraries for RNA-Seq were prepared with KAPA Stranded RNA-Seq Kit (# KR1151, Roche, Wilmington, MA, USA). The data were sequenced on Illumina HiSeq 3000 for a single read 50 run. Data quality check was done on Illumina SAV. Demultiplexing was performed with Illumina Bcl2fastq2 v 2.17 program. The reads were mapped to the latest UCSC transcript set using Bowtie2 version 2.1.0 [27] and the gene expression level was estimated using RSEM v1.2.15 [28]. TMM (trimmed mean of M-values) was used to normalize gene expression. Differentially expressed genes were identified using the edgeR program. Using Fisher’s exact test, genes showing altered expression with p < 0.05 and more than 1.5-fold changes were considered differentially expressed. The datasets are available in the Gene Expression Omnibus (GEO) repository https://www.ncbi.nlm.nih.gov/geo/query/acc.cgi?acc=GSE209989. Ingenuity Pathway Analysis (QIAGEN) was used for data analysis on the upstream regulatory pathways, and the results are provided in S1, S2 and S3 Tables.

### Flow cytometry

For cell cycle analysis, the cells were fixed in formaldehyde (4%) for 1 h, permeabilized with TritonX-100 (0.5%) for 20 min, washed, and resuspended in PBS (100 µl) containing CaCl_2_ (0.5 mM), MgCl_2_ (2.5 mM), DNase I (10 units), and RNase A (40 µg). Cells were incubated for digestion at 37 °C. After 1 h, cells were washed, resuspended in PBS-FCS-Tween 20 solution (100 µl) containing FCS (2%) and Tween-20 (0.05%), and stained with APC-conjugated BrdU antibody overnight at 4 °C. Cells were washed before resuspending in PBS containing 7-Aminoactinomycin D (7-AAD) (1.25 µg/ml) and then stained for 20 min. The cells were washed and resuspended in PBS (0.5 ml) for flow cytometry analysis.

For immune cell analysis, the fixed cells were resuspended in the PBS-FCS-Tween-20 solution (100 µl) and incubated with an a-mouse CD16/32 antibody at room temperature for 30 min to block the Fc receptors. The cells were stained with fluorochrome-conjugated antibodies against various immune markers overnight at 4 °C. The stained cells were resuspended in PBS (0.5 ml) before being analyzed by flow cytometry.

For imaging flow cytometry, the fixed cells were stained with PE-conjugated CD11b antibody overnight at 4 °C. The cells were washed and permeabilized with TritonX-100 (0.1%) before DNA staining with 4,6-diamidino-2-phenylindole (DAPI) (300 nM). After 30 min, the cells were washed again and resuspended in PBS (150 µl) before being analyzed by the ImageStream system equipped with IDEAS software (Amnis, Austin, TX, USA).

### Image acquisition and analysis of tumor vascular network

The CD31 IHC-stained tumor sections were scanned with a Leica Aperio AT2 scanner under 40X to generate the whole slide images. Leica Aperio ImageScope software was used to visualize and capture the most vascularized areas (“hot spots”) within the tumor. Granulation tissue was not considered a “hot spot” and was excluded from the analysis. Depending on the size of the tissue section, images of 1 to 4 non-overlapping hot spots were acquired by ImageScope under the 10X zoom setting and exported to the MATLAB platform for automatic morphometric analysis. The images were segmented and analyzed by the Microvessel-Segmentation MATLAB plugin [29]. The microvessel number, vascular area, and vessel wall thickness were automatically quantified. The microvessel density (MDV), expressed as the number of microvessels per mm^2^, was calculated.

### Statistical analysis

Data were analyzed using an unpaired two-tailed Student’s t-test, except for RNA-seq (see above). *p* ≤ 0.05 is considered significant. Software R was used to generate the box-and-whisker and dot plots displaying individual data points and the distribution through their quartiles.

## Results

### Spontaneous fusion between squamous cell carcinoma and myeloid cells

**Fig. 1:**
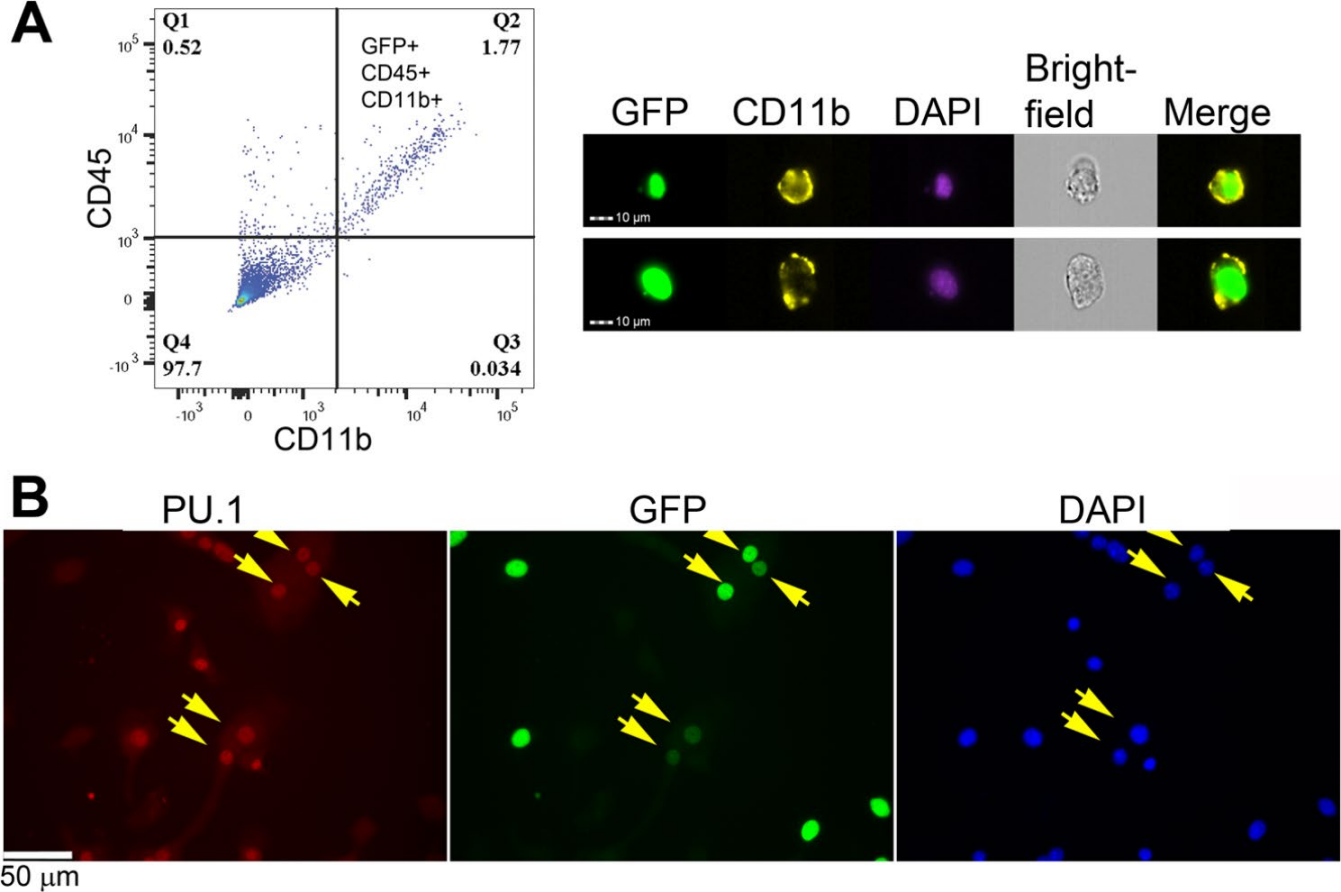
**In vivo and in vitro fusion between myeloid and SCCVII/SF cells.** Representative data are shown. (**A**) Flow cytometry analysis of tumors derived from SCCVII/SF-H2bGFP grown in C3H mice. The tumor cell suspensions were stained with appropriate antibodies (indicated in the text). Left panel: The tumor cells, gated by high GFP expression, were analyzed for co-expression of myeloid cell markers CD45 and CD11b. The proportion of CD45^+^CD11b^+^ expression in the tumor cells is indicated in the upper right quadrant. Right panel: Images of in vivo fusion between SCCVII/SF-H2bGFP cells and myeloid cells were captured by imaging flow cytometry. The tumor cell suspensions were stained with CD11b and DAPI. The double-positive hybrid cells exhibited nuclear GFP and membranous CD11b. (**B**) Co-culture of SCCVII/SF-H2bGFP cells and BMM formed multinucleated giant cells containing GFP + PU.1 + nuclei. Arrows point to double-positive nuclei

C3H mouse squamous cell carcinoma cell line SCCVII/SF was evaluated for suitability for cancer-myeloid fusion studies. SCCVII/SF-H2B-GFP cells, which are SCCVII/SF expressing nuclear GFP (H2B-GFP), were implanted in mice, and the resulting tumors were processed for flow cytometry. The hybrid cells formed by fusion between the implanted cancer cells and the host myeloid cells were expected to be GFP-positive and express myeloid markers. However, tumor cells phagocytosed by myeloid cells could also present as “pseudo-hybrids” and express these markers. In such cases, the “pseudo-hybrids” expressed GFP from remnants of SCCVII/SF-H2B-GFP cells in the phagosome; therefore, the signal was expected to be weak and cytoplasmic. Based on this assumption, the tumor cell suspensions were gated by high GFP expression followed by myeloid markers (CD45 + CD11b+) to minimize the inclusion of the “pseudo-hybrids.” Fig. 1A shows that ~ 1.77% of the GFP + cells expressed the myeloid markers, and the double-positive cells exhibited nuclear GFP and membranous CD11b. These results demonstrated that SCCVII/SF cells fused with myeloid cells in vivo and were a suitable cell line for cancer-myeloid fusion studies.

Bone marrow macrophages (BMM) require M-CSF to survive and RANKL to fuse and form multinucleated osteoclasts [30]. These cytokines were added to co-cultures of BMM and SCCVII/SF-H2B-GFP to support BMM growth and promote cell-cell fusion. Five days later, the culture formed multinucleated giant cells expressing PU.1 (macrophage marker) and nuclear GFP (from SCCVII/SF-H2B-GFP) (Fig. 1B). All the GFP + nuclei in the giant cells expressed PU.1, indicating the occurrence of nuclear fusion between BMM and SCCVII/SF-H2B-GFP cells. These multinucleated giant cells were able to survive for more than 1 month with no sign of death as long as the media was replenished. In contrast, multinucleated giant cells formed by mouse BMM in a mono-culture condition usually died after 10 days despite the support of M-CSF and RANKL.

### SCCVII/SF-BMM hybrid cell clones exhibit variable tumorigenicity

Most studies show that fusion with macrophages promotes tumor aggression [4, 6–10]. However, tumor cells fused with mesenchymal or stem cells have been reported to induce anti-tumor events [3, 11, 31]. To examine the possible outcomes from individual fusion event between SCCVII/SF and macrophages, we modified the culture condition described above, generated and cloned the hybrid cells. Briefly, BMM were retrovirally transduced to express an antibiotic resistance gene and cytoplasmic GFP before being co-cultured with SCCVII/SF cells in the presence of M-CSF and RANKL. After the culture formed multinucleated giant cells, the cytokines were withdrawn and antibiotic was added to enrich the hybrid cells, which were later cloned. The resulting cells acquired GFP expression and antibiotic resistance from the BMM; otherwise, they resembled SCCVII/SF, containing one nucleus and surviving indefinitely without the supporting cytokines.

**Fig. 2:**
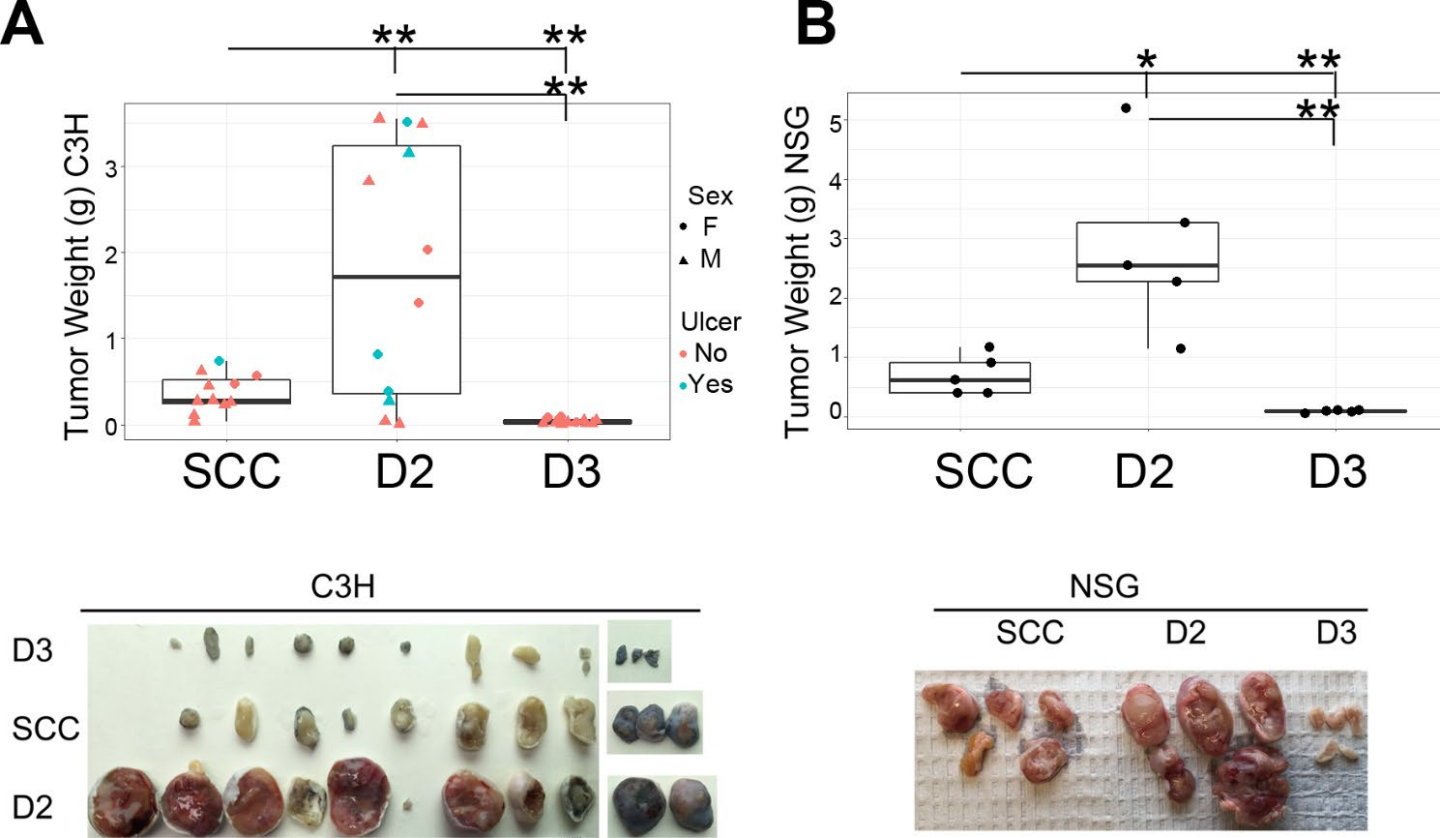
**The hybrid clones exhibited variable tumorigenicity.** SCCVII/SF-GFP (SCC), hybrid clone D2, and hybrid clone D3 cells were grown in the rear flank of C3H or NSG mice. Tumors were harvested on day 21 (C3H) or day 16 (NSG). (**A**) Top panel: Individual-value plot of tumor weight (C3H mice). Lower panel: Gross photos of the tumors. The injection site in one mouse receiving the D2 cells could not be identified, and the tissue was not included in the picture. (**B**) Top panel: Individual-value plot of tumor weight (NSG mice). Lower panel: Gross pictures of the tumors. *p ≤ 0.05, **p ≤ 0.01

Six clones of hybrid cells were implanted into the posterior dorsum of C3H mice to screen their abilities to form tumors. SCCVII/SF-GFP, which was SCCVII/SF transduced with the GFP retrovirus used for the cloned hybrid cells, served as the control. Clones D2 and D3 were selected for the current studies as they produced tumors with the most size variations from the control cells. The majority of the mice receiving the D2 cells developed significantly larger tumors (Fig. 2). Unexpectedly, ~ 16% of the C3H mice implanted with the D2 cells failed to develop grossly visible tumors within the study period of 3 weeks. In contrast, the D3 cells either failed to form tumors or produced very small tumors. The D3 cells never produced large tumors, even when the study period was extended. The growth disparities of these two hybrid cell clones were also seen when growing in the immunocompromised NSG mice.

### SCCVII/SF-BMM hybrid cells exhibited reduced growth rate and changed cell cycle distribution

RNA-seq was used to investigate the differential gene expression profiles of the hybrid cells (D2 and D3) and control cells (SCCVII/SF-GFP), analyzed by Ingenuity Pathway Analysis (QIAGEN). Selected upstream regulators pathways that showed differential activation in the hybrid cells were extracted from the analysis (S1-S3 Tables) and shown here as Table 1 for further studies.

**Table 1:** **Selected upstream regulator pathways.** The pathways predicted to be activated are listed on the left side of the z-score column, while those predicted to be inhibited listed on the right side. A z-score ≥ 2 predicts an activation state, while a z-score ≤ -2 predicts an inhibitory state of the pathway. For targeted molecule information, refer to S1-S3 Tables

RNA-Seq	Category	Upstream Regulator	Activation z-score	*p*-value of overlap
D2 vs. SCC	Cell Cycle	TP53	2.056	0.00019
	Cytokine	TNF	3.663	1.77E-11
		IL1b	2.837	0.00018
		IFNg	2.811	0.0312
		OSM	2.211	0.0156
		IL1a	2.007	0.00542
	Growth factor	VEGFa	2.771	0.00084
		EGF	2.585	0.0374
D3 vs. SCC	Cell Cycle	CDKN1A	2.532	2.2E-05
		TP53	2.928	1.34E-12
		CDKN2A	2.07	0.00735
	Cytokine	TNF	2.018	1.85E-11
	Growth factor	VEGFa	2.244	0.00153
		FGF7	-2.169	0.00779
		AREG	-3.352	9.53E-06
D2 vs. D3	Cell Cycle	CDKN1A	-2.266	5.7E-07
		TP53	-3.37	8.49E-14
		CCNK	2.366	0.00246
	Cytokine	IL6	2.021	0.0126
	Growth factor	AREG	2.917	8.30E-10
		FGF7	2.028	0.0114

**Fig. 3:**
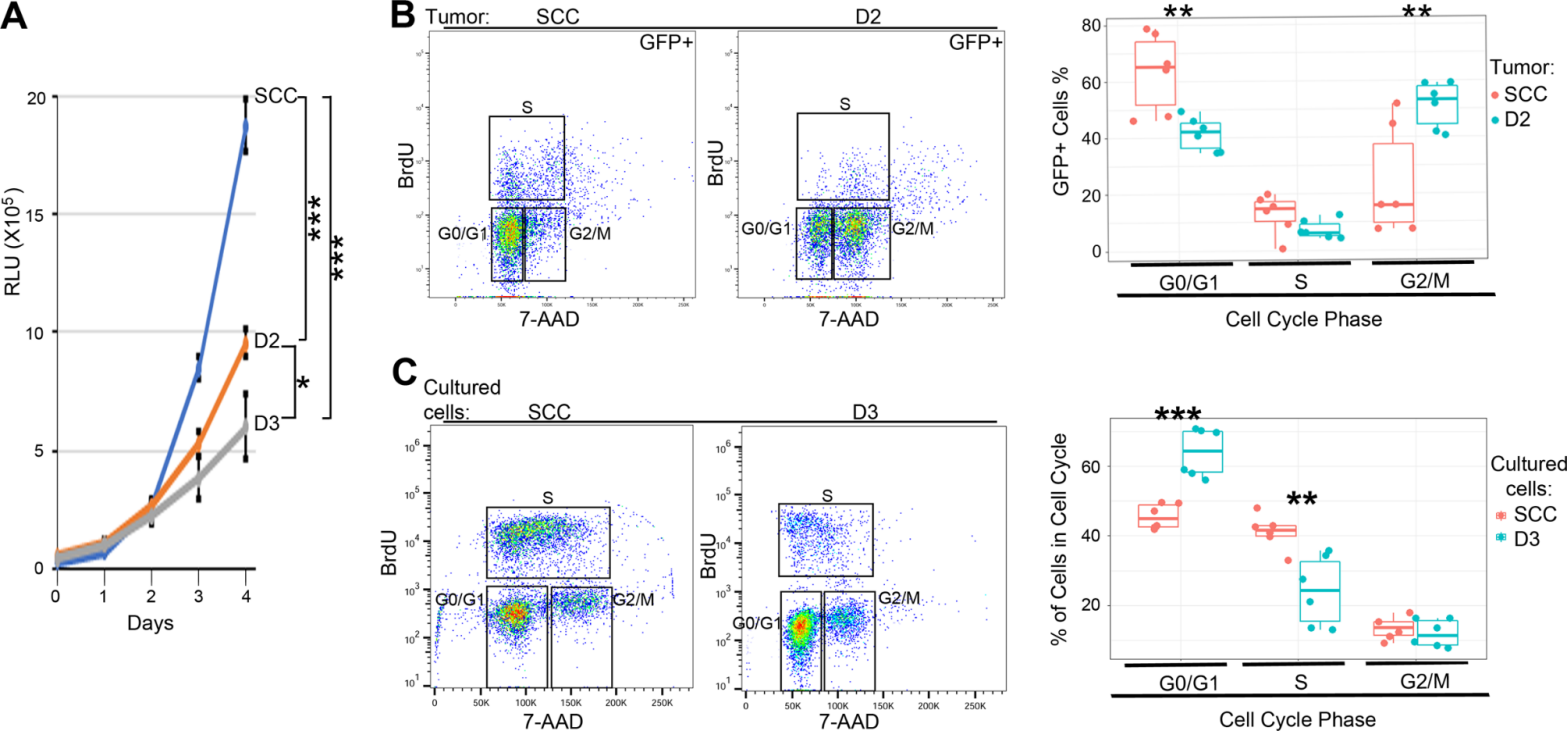
**The growths and cell cycle distributions of SCCVII/SF-GFP (SCC) and hybrid cells D2, D3.** (**A**). 4-day growth curves of SCC, D2, and D3 in cell culture. Both hybrid clones showed slow growth kinetics. Growth was evaluated by cell viability assay and expressed as relative luminescent units (RLU). The day 4 data were used to calculate the *p*-values. (**B**). Cell cycle distribution of tumor SCC and D2 cells (GFP+) (left panel) and the quantified results (right panel). (**C**). Cell cycle distribution of the cultured SCC and D3 cells (left panel) and the quantified results (right panel). *p ≤ 0.05, **p ≤ 0.01, ***p ≤ 0.001. Representative data of biological replicates were shown for flow analysis

Increased activation of the TP53 pathway was detected in both hybrid cells (Table 1), presumably caused by the abnormal chromosome numbers [32, 33]. As TP53 regulates cell growth and plays a critical role in the checkpoints [34, 35], its impacts on the growth and cell cycle of the hybrid cells were investigated. The growth of the hybrid cells was studied using a 4-day growth curve experiment. The results showed that while all studied cells were within their log phases of growth, D2 and D3 cells exhibited slower growth and prolonged doubling times (D2 ~ 23.97 h, D3 ~ 28.31 h) than the control cells (~ 16.79 h) (Fig. 3A).

The impact on the cell cycle distribution of the hybrid cells was analyzed by flow cytometry. As in vivo material is preferred over in vitro material for investigating biological activity, tumor tissue harvested from the mice was used whenever possible, as was the case for the D2 cells. In contrast, the D3 cells either did not form tumors or formed tumors that were too small to generate sufficient cells for flow cytometry; cultured cells were used instead for flow analysis. The cell cycle analysis showed that the tumor D2 cells (GFP+) had increased presence in the G2/M phase while decreased presence in the G1/G0 phase compared with the tumor control cells (Fig. 3B). In contrast, their stromal cells (GFP-) showed no differences in the cell cycle distribution, with the majority of the cells in G0/G1 while only a minority transitioned through the G2/M phase (S1 Fig). Similar to the D2 cells, the D3 cells also showed different cell cycle distributions from the control cells. Unlike the D2 cells, the D3 cells had an increased presence in the G0/G1 phase and decreased presence in the S phase compared with the control cells (Fig. 3C).

### SCCVII/SF-BMM hybrid cells show an increased ability to recruit stromal cells

The RNA-seq results also showed that the D2 and D3 hybrid cells exhibited increased activation of a number of cytokine and growth factor pathways (Table 1). As BMM is a known source of these cytokines and factors, the hybrid cells presumably acquired the capability during the cancer-BMM fusion process. Among these pathways, TNF and VEGF-a activations were seen in both D2 and D3 cells. TNF is a multifunctional cytokine playing important roles in diverse cellular events, ranging from proinflammatory effects on inflammatory cells to mitogenic effects on fibroblasts [36–38]. VEGF-a is known to promote the proliferation and migration of vascular endothelial cells, and its function is enhanced by inflammation [39]. Upregulation of these pathways potentially could promote the complexity of the tumors derived from the hybrid cells.

Immunofluorescence stain and flow cytometry were used to examine the cellular composition of the tumors derived from the hybrid cells. The D3 tumors were only examined by immunofluorescence staining as they were too small to generate enough cells for flow analysis.

**Fig. 4:**
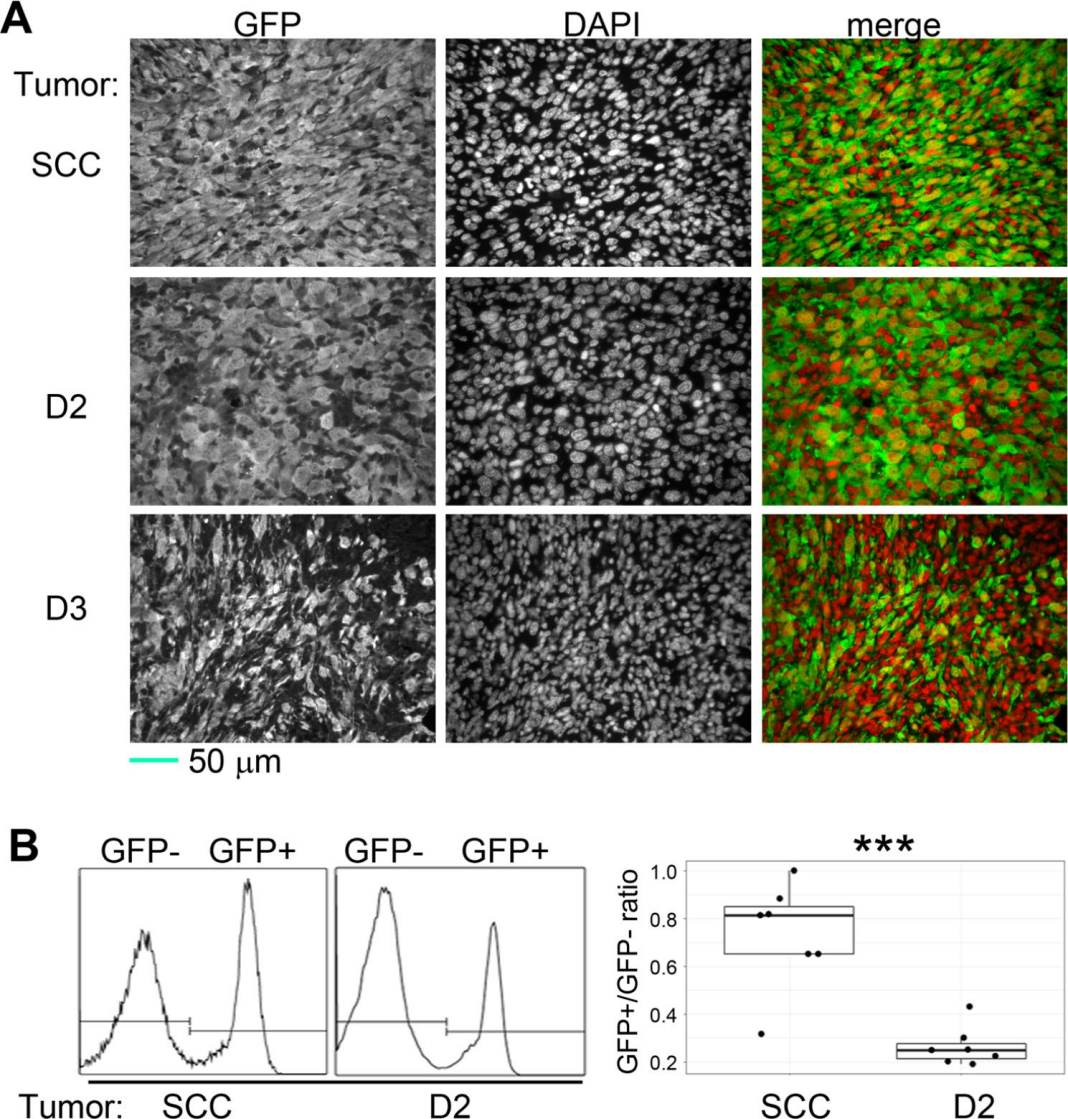
**The majority of the mass in the hybrid cell-derived tumors comes from non-tumor cells.** (**A**) Tissue sections of SCCVII/SF-GFP (SCC), D2 and D3 tumors were stained with anti-GFP antibody and DAPI (nuclear stain), and the resulting signals were pseudo-colored as green and red, respectively. Tumor cells: yellow nuclei; non-tumor cells: red nuclei. (**B**) Left panel: GFP expression was used to gate the cell suspensions to differentiate between the tumor cells (GFP+) and non-tumoral cells (GFP-). These are representative data of biological replicates. Right panel: GFP+/GFP-: The ratios between the tumor and non-tumor cells. ***p ≤ 0.001

GFP expression was used to distinguish the tumor cells (GFP+) from the non-tumor stromal cells (GFP-). DAPI was used to stain the nuclei and was pseudo-colored as red signals so that the tumor cells could be identified by their yellow nuclei, differentiated from the non-tumor cells’ red nuclei (Fig. 4A). The immunofluorescence stains showed that the D3 tumor had the most abundant presence of red nuclei (the non-tumor cells). The percentages of the non-tumor cells in the D2 and control tumors were analyzed by flow cytometry. The quantification showed that the D2 tumor, like the D3 tumor, also exhibited a higher presence of non-tumor (GFP-) cells and a significantly lower GFP+/GFP- ratio than the control tumor (Fig. 4B). These results indicate that the large size of the D2 tumor did not result from a high quantity of the tumor cells. Instead, the bulk mass was mostly composed of the non-tumor cells. Similarly, the D3 cells also exhibited an increased ability to recruit the stromal cells despite their minimal tumorigenicity.

As the RNA-seq data indicated that the hybrid tumors had activation of a number of proinflammatory cytokine pathways, immunohistochemistry and flow cytometry were used to analyze the presence of several major kinds of inflammatory cells in the tumors. For the reason stated before, the D3 tumor was only studied by immunohistochemistry.

**Fig. 5:**
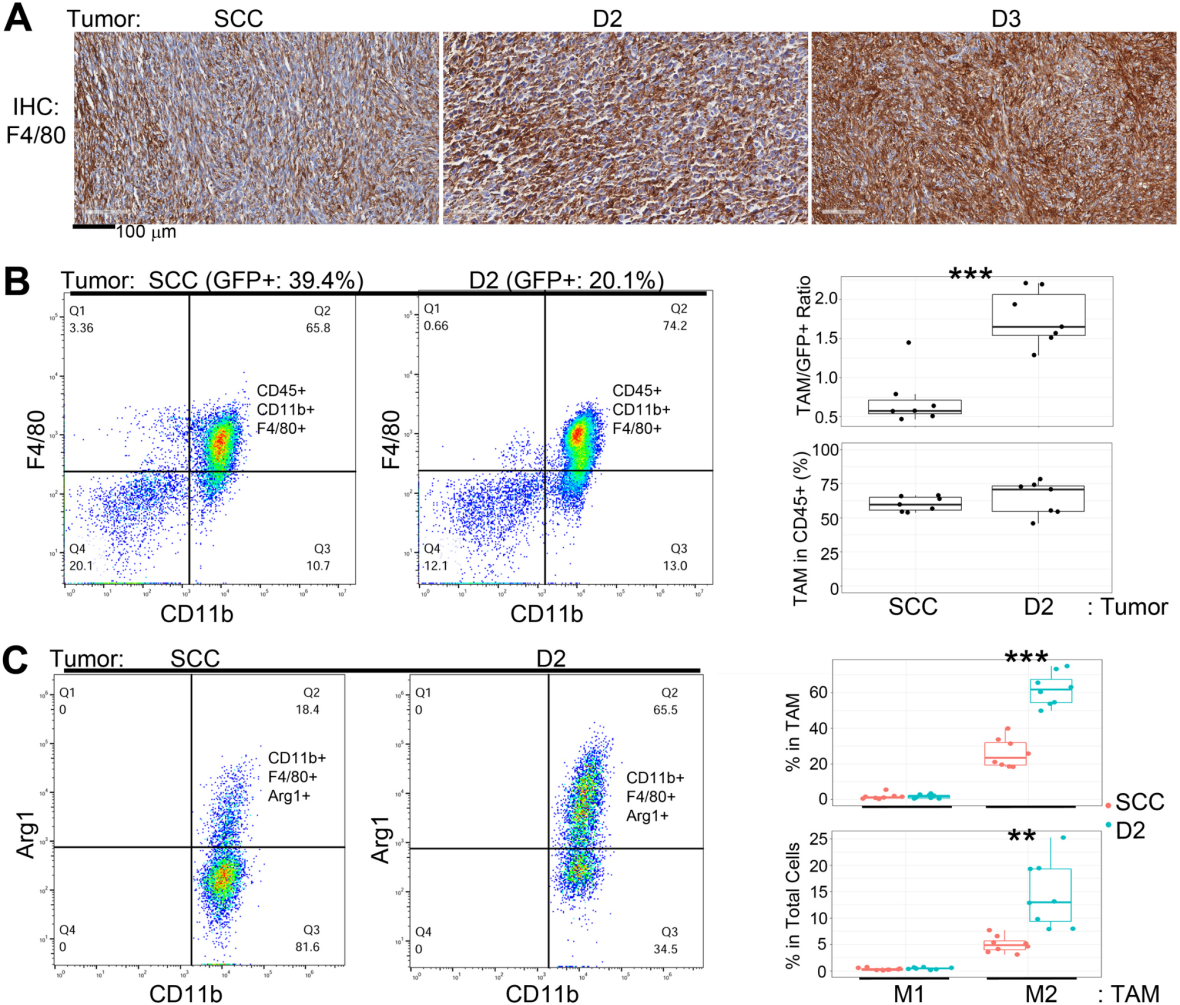
**TAM in tumors formed by SCCVII/SF-GFP (SCC), D2, and D3 cells.** (**A**) F4/80 IHC was used to stain TAM in the tumor tissue sections. (**B**) Flow cytometry analysis of TAM. Left panel: TAM (CD45 + CD11b + F4/80+) are shown in the upper right quadrant of the flow chart. Top label shows GFP + percentages in the tumor. Right panel: Quantified results of TAM/GFP + ratio (top) and TAM percentage in total leukocytes (CD45+) (lower). (**C**) Flow cytometry analysis of TAM polarization. Left panel: M2 TAM (CD11b + F4/80 + Arg1+) are shown in the upper right quadrant of the flow chart. Right panel: The percentages of M1 and M2 cells in total TAM (top). The percentages of M1 and M2 cells in total cells (sum of tumor and non-tumor cells). Representative data of biological replicates were shown for flow analysis. **p ≤ 0.01, ***p ≤ 0.001

F4/80 IHC was used to stain the macrophages, revealing much more intense staining in the hybrid tumors than in the control tumor (Fig. 5A). The signal was especially strong in the D3 tumor. Flow cytometry was used to quantify the number of tumor-associated macrophages (TAM) in the D2 and control tumors, conducted by gating the tumor cell suspensions with GFP and macrophage markers (CD45 + CD11b + F4/80+). The results showed that the D2 tumor had a significantly higher ratio of TAM/GFP+, indicating that the D2 cells, similar to the D3 cells, were more capable of recruiting TAM than the control cells (Fig. 5B). There were no differences in the percentages of TAM in total leukocytes (CD45+) between the D2 and control tumors (Fig. 5B).

Furthermore, TAM polarization was examined by gating the tumor cell suspensions with M1 (CD11b + F4/80 + iNos+) and M2 (CD11b + F4/80 + Arg1+) macrophage markers (Fig. 5C). The results showed that the D2 tumor had a higher percentage of M2 in TAM (top) and total cells (lower) than in the control tumor. Most of the TAM were M2. In contrast, M1 macrophages were not significantly present, and their numbers were not different between the D2 and the control tumors.

**Fig. 6:**
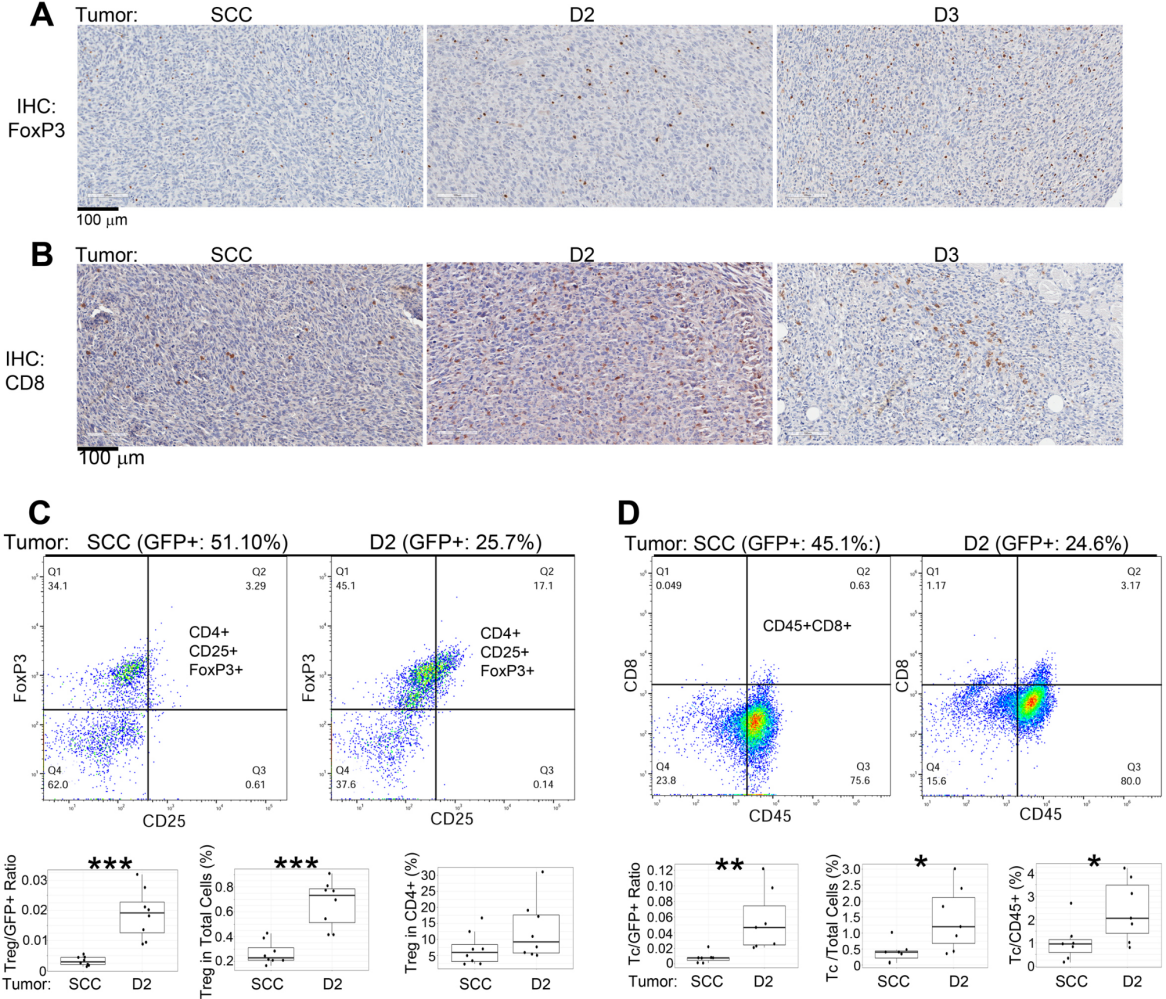
**Treg and Tc in tumors formed by SCCVII/SF-GFP (SCC), D2, and D3 cells.** (**A**). FoxP3 IHC was used to show Treg in the tumor tissue sections. (**B**). CD8 IHC was used to show Tc cells in the tumor tissue sections. (**C**) Upper panel: flow cytometry shows Treg (CD4 + CD25 + FoxP3+) in the upper right quadrant. The top label shows GFP + percentages in the tumor. Lower panel: Quantified results of Treg/GFP + ratio, Treg percentage in total cells, and Treg percentage in helper T cells. (**D**) Upper panel: flow cytometry shows Tc (CD45 + CD8+) in the upper right quadrant. The top label shows GFP + percentages in the tumor. Lower panel: Quantified results of Tc/GFP + ratio, Tc/total cell ratio, and Tc percentage in total leukocytes (CD45+). *p ≤ 0.05, **p ≤ 0.01, ***p ≤ 0.001. Representative data of biological replicates are shown for flow analysis

FoxP3 and CD8 IHCs were used to qualitatively assess Treg and Tc in the tumors, respectively (Fig. 6A and B). T cells in the D2 and control tumors were also analyzed by flow cytometry: Treg was identified by CD4 + CD25 + FoxP3 + expressions, Tc was identified by CD45 + CD8 + expressions, and Th was identified by CD45 + CD4 + expressions (Fig. 6 C and 6D upper panels). Similar to the TAM results, the D2 tumor also exhibited higher numbers of Treg and Tc per tumor cells (GFP+), indicating an increased ability to recruit these T cells (Fig. 6 C and 6D lower panels). While the D2 tumors also showed higher percentages of these T cells in total cells and a slight increase of Tc among total leukocytes (CD45+), there was no difference from the control tumor with the percentage of Treg in the helper T cells (CD4+). Compared to TAM, tumor-infiltrating Treg and Tc cells were relatively lower numbers, and NK cells were not even detected. The scant presence of NK cells was not unexpected for SCC-VII/SF-derived tumors since they produce high levels of TGF-β, suppressing NK cells [40, 41].

### Tumors derived from SCCVII/SF-BMM hybrid cells exhibit increased angiogenesis

**Fig. 7:**
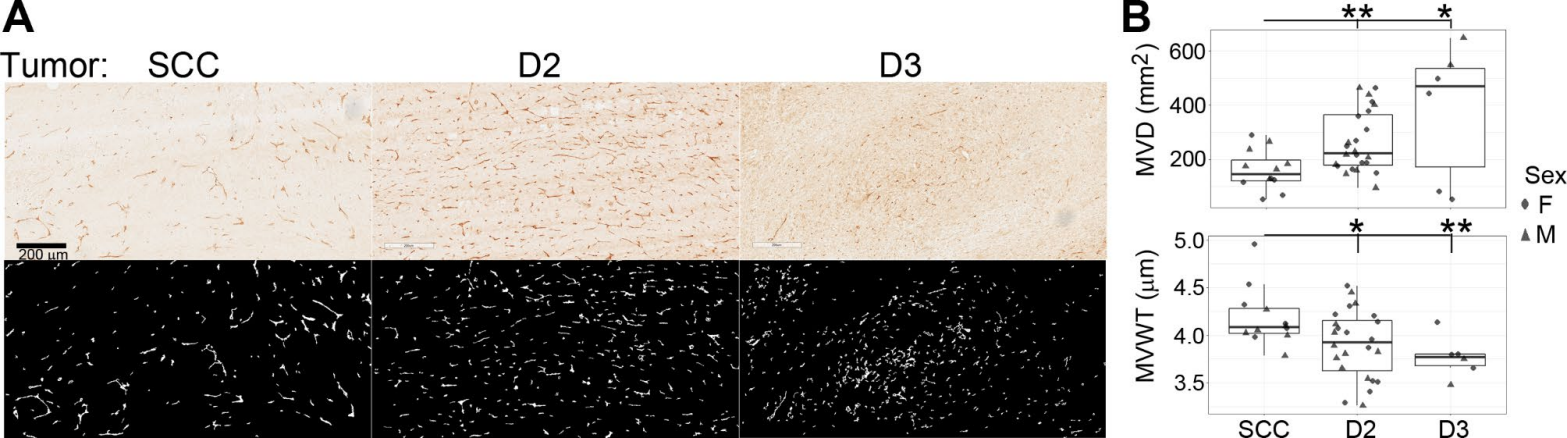
**Vasculature in tumors formed by SCCVII/SF-GFP (SCC), D2, and D3 cells.** (**A**) CD31 IHC: Top panel: bright field view. Lower panel: computer-generated segmented CD31 + Signals. Representative data of biological replicates are shown. (**B**). Quantified results of micro-vessel density (MVD) and micro-vessel wall thickness (MVWT). Each dot on the scatter plot represents the averaged data value generated from one mouse. Since the D3 hybrid cells often failed to form tumors, the number of animals allowed for analysis was smaller than those used for the D2 and control cells. F: female, M: male. *p ≤ 0.05, **p ≤ 0.01

VEGF-α is an essential angiogenic factor [39] and the RNA-seq data indicated that both D2 and D3 hybrid cells had increased activation of this pathway (Table 1). CD31 IHC, which stains endothelial cells, was used to examine the micro-vessels in the tumors (Fig. 7A, top panel). The IHC images were segmented (Fig. 7A, lower panel) before being analyzed by the Microvessel-Segmentation MATLAB plugin [29]. The results show that tumors formed by the hybrid cells, despite their considerable size differences, exhibited higher microvessel densities (MVD) and thinner micro-vessel wall thickness (MVWT) than the control tumors (Fig. 7B). There were no significant differences in other angiogenesis parameters, such as micro-vessel sizes and percentage of vessel area.

## Discussion

This study examined the diverse biological consequences of fusion between cancer and myeloid cells. The hybrid cancer-BMM cells were generated in vitro and began as multinucleated cells. At this stage, the cell markers from the parental cells were detectable. Multinucleated hybrid cells had minimal chance of being successful in proliferation due to their heterokaryon status, which leads to multipolar division during chromosome segregation [42]. For proliferation to be successful, the heterokaryon-type hybrids need to merge the nuclei into one common nucleus and become synkaryons. Antibiotic selection was used to enrich the synkaryon-type hybrids in the culture, which were subsequently cloned for further study of phenotypes.

Chromosome loss often occurs during the transition from heterokaryon to synkaryon, which leads to aneuploidy, activation of TP53, and alterations in cell cycle [32, 33]. Both hybrid cell clones had lost their myeloid markers and exhibited slow growth kinetics (Fig. 2A). Paradoxically, while the D3 cells showed minimal tumorigenicity, the D2 cells formed large tumors in mice. The drastic differences in tumorgenicity were observed in syngeneic C3H and immunocompromised NSG mice, indicating that intrinsic mechanisms, not host immunity, were the main cause of the growth differences. Compared with the D2 cells, the D3 cells had smaller nuclei (S2 Fig) and higher activation levels of TP53 and CDKN1A pathways (Table 1). The smaller nuclear sizes might be related to more chromosome loss in the D3 cells, which led to high levels of TP53 and, subsequently, CDKN1A activation [34, 43]. High levels of CDKN1A are known to lead to G0/G1 arrest, which may cause the D3 cells’ increased presence in the G0G1 phase and minimal tumorigenicity in mice. In contrast to the D3 cells, the D2 cells not only could form tumors, but the tumor sizes were significantly larger than the control cells. The D2 cells had less extensive TP53 activation. Despite increased distribution in the G2/M phase and slowed growth kinetics, the D2 cells were able to grow and form large tumors in mice by recruiting high numbers of stromal cells, possibly due to the activated cytokine and growth factor pathways acquired from the parental BMM. The D2 cells also exhibited higher levels of cyclin K (CCNK) than the D3. It is unclear whether or not CCNK activation plays a role in the cell cycle progression of the D2 cells [44].

Surprisingly, the large tumors formed by the D2 cells had a high stromal content, a feature also seen in the small tumors formed by the D3 cells. Most of the stromal cells were presumably fibroblasts since they are the most abundant cell type in solid tumors [45], which may be established via the activated TNF pathway in the hybrid cells [36–38]. Despite the fact that the D2 cells often produced large tumors, ~ 16% of the mice that received the D2 cells failed to develop grossly visible tumors within the study period, a phenomenon seen in most mice receiving the D3 cells. Therefore, the D2, although less frequently, could also develop severe chromosomal instability that was significant enough to stall tumor growth. In this study, we did not pursue the roles of bone marrow-derived mesenchymal cells in the biological behavior of the hybrid cells. They were scantly present in the BMM-cancer co-culture used to generate the hybrid cells. Although unlikely, we could not completely rule out the possibility that they also fused with the SCCVII/SF-BMM hybrids and played roles in tumorigenicity.

In this study, we showed that tumor-BMM fusion could enhance the complexity of the tumoral contents via boosting angiogenesis and increased establishment of stromal cells, which provide tumor cells diversified pathways to gain survival advantages in response to an ever-changing environment. Thus, tumor-myeloid fusion has important biological implications for cancer survival and evolution. While some hybrid cells gained functions in pro-tumoral pathways, they also inadvertently triggered cell cycle checkpoints and altered their growth kinetics. Despite this, the pro-tumor microenvironment created by these hybrid cells could provide growth benefits to the existing tumor cells. This study underscores hybrid cells’ potential supportive roles in altering the tumoral microenvironment. Hypothetically, the particular hybrid cells do not necessarily have to persist for a long time. As long as the replenishment of new hybrid cells continues, the pro-tumor characteristics of stroma could be maintained while waiting for accumulations of genetic and epigenetic changes that lead to the emergence of rare cells for tumor progression.

## Conclusion

In conclusion, hybrid cells resulting from tumor-myeloid fusion are potent environmental modifiers. Their presence, even a temporal one, provides significant advantages to other tumor cells in promoting tumor survival and allowing the escape of rare cells, which could potentially be the source of tumor recurrence or metastasis. Knowledge of tumor-myeloid fusion may help develop novel clinical interventions to control cancer progression. Moreover, our study has revealed that tumor-myeloid fusion can activate various pathways involving cell cycle, cytokines and growth factors, and it also contributes to tumorigenicity. As a future direction for our research, we plan to investigate the fate and functionality of immune and stromal cells that are altered after tumor-myeloid fusion, as this will shed light on the broader implications of this phenomenon in cancer progression.

## Electronic supplementary material

Below is the link to the electronic supplementary material.

Supplementary Material 1

Supplementary Material 2

Supplementary Material 3

Supplementary Material 4

Supplementary Material 5

Supplementary Material 6

Supplementary Material 7

Supplementary Material 8

Supplementary Material 9

Supplementary Material 10

Supplementary Material 11

Supplementary Material 12

Supplementary Material 13

Supplementary Material 14

## Data Availability

The RNA-seq datasets generated during the current study are available in the GEO repository https://www.ncbi.nlm.nih.gov/geo/query/acc.cgi?acc=GSE209989. Upstream regulatory pathway analysis results are provided as supplementary tables (S1, S2 and S3 Tables). Other IPA analyses (Canonical Pathways, Disease and Function and Network) are also provided as supplemental files (Excel files). All other data that support the findings of this study are available from the corresponding author on reasonable request.
