# Supplementary material for: The impact and outcomes of cancer-macrophage fusion
Source: BMC Cancer. 2023 Jun 1;23:497. doi: 10.1186/s12885-023-10961-9 (PMC10236829; doi:10.1186/s12885-023-10961-9)

**Supporting information**

**S1 Fig. Cell cycle distribution of the stromal cells (GFP-) in the tumors formed by SCCVII/SF-GFP (SCC) and D2 cells in C3H mice.** Left panel: representative flow cytometry data of the stromal cells (GFP-). Right panel: quantified results.


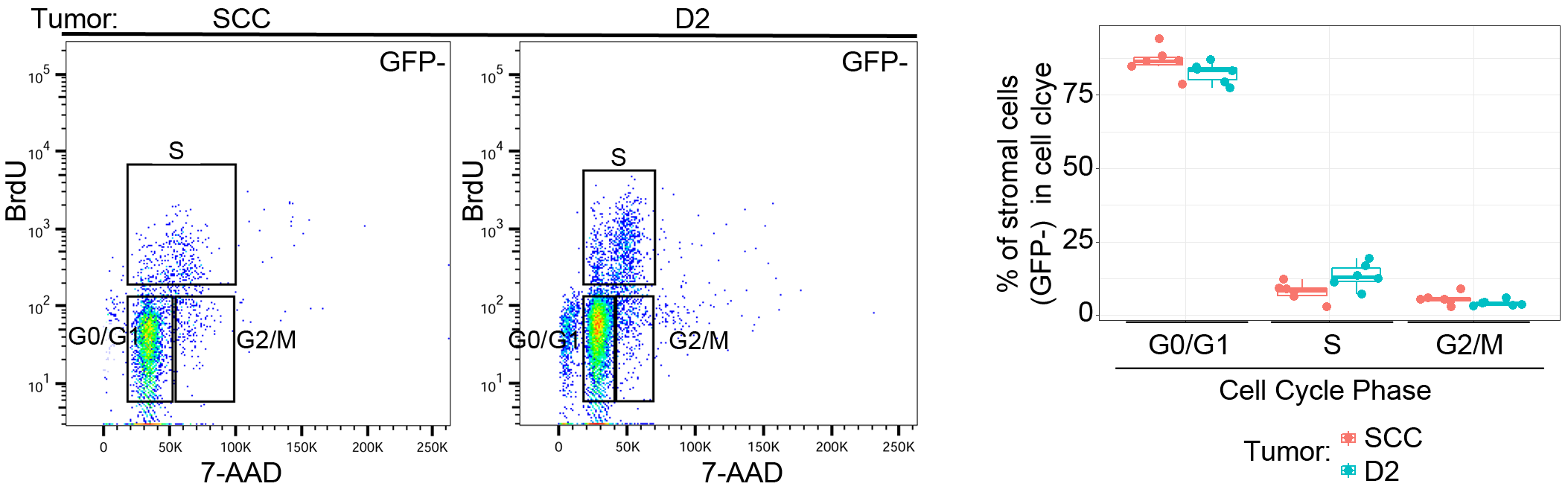

Supplement: Supplementary file 1 — Supplementary Material 1 [file 12885_2023_10961_MOESM1_ESM.docx]
