# Supplementary material for: The impact and outcomes of cancer-macrophage fusion
Source: BMC Cancer. 2023 Jun 1;23:497. doi: 10.1186/s12885-023-10961-9 (PMC10236829; doi:10.1186/s12885-023-10961-9)
Supplement: Supplementary file 2 — Supplementary Material 2 [file 12885_2023_10961_MOESM2_ESM.docx]

**Supporting information**

**S2 Fig. Nuclear sizes of control (SCCVII/SF-GFP), D2, and D3 cells.** Nuclear sizes are expressed as relative values (pixel^2^). ****p*≤0.001.
