# Supplementary material for: The impact and outcomes of cancer-macrophage fusion
Source: BMC Cancer. 2023 Jun 1;23:497. doi: 10.1186/s12885-023-10961-9 (PMC10236829; doi:10.1186/s12885-023-10961-9)
Supplement: Supplementary file 3 — Supplementary Material 3 [file 12885_2023_10961_MOESM3_ESM.pdf]

Supporting information

S1 Table. Upstream regulatory analysis of D2 hybrid cells vs. SCCVII/SF-GFP. The RNA-seq data analyzed by Ingenuity Pathway Analysis (QIAGEN).

© 2000-2018 QIAGEN. All rights reserved.

Upstream Regulator	Expr Fold Change	Molecule Type	Predicted Activation State	Activation z-score	p-value of overlap	Target molecules in dataset	Mechanistic Network
TNF		cytokine	Activated	3.663	1.77E-11	A4GALT,ACTA2,ALDH2,APLN,BTG2,BTG3,C3,CCL17,CCL5,CCND3,CDH11,CFB,CLEC11A,COL1A2,CSF1,CX3CL1,CXCL16,CXCL2,CYP27A1,DBI,DUSP1,DUSP5,EGR1,FAS,FLT1,FOS,FST,GADD45A,GSTM2,HBEGF,IER3,IFIT3,IL1RN,ITGB3,LAMB3,LBP,MARCKSL1,MMP13,MMP3,NOTCH1,NR1H4,OAS1,OAS2,OSMR,PIK3CD,PLAU,RBPMS,RGS2,RHOB,SEMA3C,SERPINB1,SERPINE1,SOX4,TERT,TIMP3,TNFRSF11B,TNFSF10,TSLP,VEGFC,WISP1	141 (12)
TGFB1		growth factor		1.371	3.93E-10	ACTA2,ADAM19,AFP,ASPN,BCL2L11,BGN,CCL5,CCND3,CDC42SE1,CDH11,COL18A1,COL1A1,COL1A2,DOCK4,FAS,FBN1,FLT1,FOS,FSCN1,HBEGF,HEY1,IL1RL1,IRS1,ITGB2,ITGB3,LTBP1,MMP13,MMP3,MRC1,NRP1,PDGFB,PDLIM4,PIK3CD,PLAU,PTP4A3,PTPRK,RHOB,RHOD,SERPINA1,SERPINE1,SOX4,TERT,TGFBI,TGM2,THPO,TIMP3,TSPAN7	142 (12)
TP73		transcription regulator		1.164	2.03E-09	ABCB1,ACTA2,ADA,CCND3,CDC42EP2,CLMN,COL1A1,CTH,CXCL2,DRAM1,FAS,FKBP1B,FST,GRK5,HBEGF,HES1,IGFBP4,ITGB4,LTBP1,NAP1L3,PDGFB,PIEZO2,PTEN,PTP4A3,SERPINA1,SERPINE1,SFN,TERT,VEGFC	101 (5)
TRIB3	-3.914	kinase	Activated	2.954	7.27E-09	ASNS,CTH,GARS,GDF15,MTHFD2,PCK2,PSPH,STC2,TRIB3	
NEUROG1		transcription regulator		-1.291	1.79E-07	ADD3,C1S,C3,CEMIP,CFH,DSP,FAM198B,GREM1,ITGB3,LRRN3,P4HA2,SERPINF1,SULF1,TMTC2,TSPAN7	
estrogen receptor		group		-0.207	3.12E-07	ABCA1,ABCG2,C3,CA12,CDH11,CNKSR1,COL12A1,COL4A6,COL6A2,DCHS1,DSP,FGF1,FOS,GREB1,HBEGF,JUP,KRT18,LY6E,MAPK12,MAPT,MMP15,PCDH10,PCDH7,PLAU,RGS2,SERPINE1,TERT,TIMP3,VEGFC	
STAT5A		transcription regulator			3.2E-07	ABCB1,ALDH3A1,C15orf48,CDH17,CEACAM1,CEMIP,CISH,EGLN3,FAM198B,FKBP14,FOXQ1,GCHFR,GCNT3,HIST1H1C,HIST2H2AA3/HIST2H2AA4,KRT80,LAMB3,MAP1LC3A,OAS1,TGFBI,TXNIP	
SMAD3		transcription regulator		0.952	3.57E-07	ACTA2,ASPN,C3,COL1A1,COL1A2,DOCK4,HEY1,MMP13,PTP4A3,PTPRK,RHOB,SERPINE1,TERT,TIMP3,VEGFC	

HNRNPA2B1		other			1.78E-06	ABCB1,CA12,CEMIP,CLDN1,CP,CRIP1,DNER,ELOVL7,FSCN1,GDF15,GJA1,HYAL1,INHBB,JUP,NR3C2,OAS1,PDE1A,PLCB4,SEMA3B,SERPINA1,SYT1,WNT4	
CBX5		transcription regulator		-1.199	1.83E-06	ABCB1,ALDH3A1,C15orf48,CDH17,CEACAM1,CEMIP,FAM198B,FKBP14,FOXQ1,GCHFR,GCNT3,HIST1H1C,HIST2H2AA3/HIST2H2AA4,KRT80,LAMB3,MAP1LC3A,OAS1,TGFBI,TXNIP	
F7		peptidase		0.781	2.75E-06	AR,CXCL2,EGR1,FOS,FOSL1,GADD45A,HBEGF,MMP13,PTGER2	40 (4)
ATF4		transcription regulator	Inhibited	-2.189	2.75E-06	ASNS,CA9,CHAC1,FUT7,PCK2,PSPH,SLC1A4,SLC1A5,SLC7A11	
FOXO3		transcription regulator		1.746	3.61E-06	AR,BCL2L11,CLDN1,EGR1,EGR2,GADD45A,GADD45B,IER3,MMP13,OVOL1,SERPINE1,TNFSF10,TXNIP	
TREM1		transmembrane receptor		0.684	4.3E-06	ASNS,ATP1B1,CCL17,CCL5,CFB,CSF1,CXCL2,EGR1,EGR2,EGR3,ELOVL7,FOSL1,GADD45B,GREM1,HBEGF,HES1,HSD3B7,IL15RA,LY9,OSGIN1,SLC1A3,SPP1,TNFSF15,TNIP3	
FSH		complex		-1.672	5.26E-06	ACTA2,ADAMTS1,AMOTL2,ATP9A,BDNF,BTG2,CARD10,COL18A1,DUSP1,EZR,GATA6,GRK5,INHA,INHBB,KRT18,MAP3K5,P4HA2,PIK3CD,PTGER2,PTPRF,PTPRN,RAB27A,RASAL2,RASSF2,RGS4,RGS7,RHOB,TNFRSF11B,TP53I11,UPP1,VEGFC,WT1	
PDGF BB		complex	Activated	2.287	5.35E-06	CYR61,DUSP1,DUSP5,EGR1,EGR2,EGR3,FOS,FOSB,GADD45A,IER3,PLAU,RGS2,RHOB,SLCO1A2,THPO	
Lh		complex		-1.133	5.62E-06	ACTA2,AR,ATP9A,CARD10,COL18A1,DUSP1,EZR,GJA1,GRK5,INHA,INHBB,KRT18,MAP3K5,P4HA2,PIK3CD,PTPRF,PTPRN,RAB27A,RAPGEF3,RASAL2,RGS4,RGS7,RHOB,TNFRSF11B,TP53I11,UPP1,VEGFC	
TP63	8.063	transcription regulator	Activated	2.17	0.000012	ADA,ADAMTS1,BCL2L11,CCND3,CYR61,DLX1,FAS,FBN1,FOSL1,FST,GADD45A,HBEGF,HES1,ID3,IER3,ITGB4,ITGB8,MMP13,NOTCH1,PLAU,POSTN,PTEN,SERPINE1,SFN,TINAGL1,TNFSF10,TP63,WNT4	132 (11)
SMAD1		transcription regulator		0.339	1.54E-05	ACTA2,BTG2,COL1A1,COL1A2,CXCL2	
TGFBR2		kinase		1.253	2.35E-05	ACTA2,BDNF,CDHR1,COL4A6,GADD45B,GATM,JUP,MAP3K11,MMP3,PDGFB,RBMS3,SERPINE1,TXNIP	

P38 MAPK		group	Activated	3.139	2.59E-05	CCL5,CXCL2,EGR1,EGR2,FAS,FOS,FST,IER3,ITGB3,ITGB4,ITGB8,KDELR3,MMP13,MMP3,PLA2G4A,PLA2G7,SERPINE1,TERT,TNFSF10,TSLP,VDR	170 (14)
NFIL3		transcription regulator			0.00003	FAS,GADD45A,GADD45B,TNFSF10	14 (2)
IGF1		growth factor		1.054	3.51E-05	BCL2L11,EFNB2,ELN,FOS,GAP43,IRS1,IRS2,PLAU,SERPINE1,SFN,SYN1,TUBB3	65 (8)
ERK		group		1.147	4.55E-05	BCL2L11,COL1A1,CXCL2,DUSP1,EGR1,FAS,FOS,FOSL1,FST,GDF15,HBEGF,ITGB2,ITGB3,MMP13,SERPINE1,TGM2	115 (10)
JUN		transcription regulator	Activated	2.607	4.83E-05	ABCB1,ASNS,CCND3,DUSP1,DUSP5,FOSL1,FTH1,GJA1,GPT,ITGB4,ITGB8,LTBP1,MMP13,NFKBIZ,SERPINE1,SPP1,SULF2,VAV3,ZNF385A	99 (6)
NR4A1		ligand-dependent nuclear receptor		0.198	5.26E-05	ACTA2,CCL5,COL1A1,COL1A2,SERPINE1,TNFSF10	
YBX1		transcription regulator		1.23	6.43E-05	ABCB1,ACTA2,CCL5,COL1A2,EFEMP1,FOS,MMP3,STC2	
SYK		kinase		-0.269	7.39E-05	ATP8A1,BCL2L11,CCL5,DOCK10,FOSL1,FST,GADD45A,OAS1,TNFSF10,TP53INP1,TRIB2	21 (2)
FGF2		growth factor		1.915	8.35E-05	BGN,DCN,EFNB2,EGR1,FLT1,PLAU,SERPINE1,TFPI,THPO	
FGF8		growth factor		-0.632	9.02E-05	AIF1L,COL18A1,CRIP1,LAMB3,RUNX3,SLPI,SPP1,TM7SF2,TNXB,VDR	
SMARCA4		transcription regulator	Activated	2.681	0.000103	ABCA1,ABCB1,ALDH2,ASNS,C6orf141,CD74,CP,CXCR6,EFNB1,EGR1,EMP3,ESPNL,FAS,FOS,GADD45A,GCHFR,GJB5,GULP1,HEPH,ID3,IER3,IFITM3,IGFBP4,IL15RA,KRT18,NAP1L3,NFKBIZ,NRP1,PDE4B,PKIA,PLAT,RAMP1,RGS2,SEMA3B,SERPINB7,SOD3,SPP1,STAMBPL1,STXBP6,TFB1M,TRNP1	
CLDN7		other		-0.729	0.000111	ABLIM1,ALDH3A1,C1S,C3,CA12,CX3CL1,FOSL1,FOXQ1,HIST2H2AA3/HIST2H2AA4,MCU,NETO2,NNMT,RPP40,SERPINB1,SERPINB7,TEAD2,TINAGL1	
VTN		other		1	0.000112	EFNB2,IRS1,ITGB3,SERPINE1,SFN	
TLR3		transmembrane receptor	Activated	2.586	0.000115	C3,CCL5,CFB,CX3CL1,DHX58,FOS,IFIT3,LIPA,OAS1,PIK3CD,SLPI,TNFSF10,TP63,TSLP	150 (12)

NOTCH1	3.853	transcription regulator		1.127	0.000144	ACTA2,CHST1,EFNB2,HES1,HEY1,MMP3,PDGFB,PTPRK,RUNX3,TP63,TSLP	23 (2)
EPAS1		transcription regulator		0.067	0.000147	CA9,CEMIP,CKMT1A/CKMT1B,CLDN1,EGLN3,FOS,GJA1,INHBB,IRS2,PFKFB3,SERPINE1,SLC7A5,STC2,WISP2	
Cdk		group		1.195	0.000168	CCL5,CXCL2,DUSP1,GADD45A,GADD45G,RGS2,TXNIP	
DUSP1	5.249	phosphatase		-0.97	0.000168	DUSP1,GDF15,NRP1,PLAT,PTPRK,VEGFC,WISP2	
SOX11		transcription regulator		0	0.000168	ADSS,CCL5,EGR1,FAS,NIPSNAP1,NREP,RNF122,SPIB,TIAM1,TP53INP1,TUBB3,YPEL1	
ZNF217		transcription regulator		-0.816	0.000168	ATL1,KRT18,NEFL,NMNAT2,PLAT,SEC14L2,SH3RF2,SHC4,STRA6,TDGF1,ZHX2,ZNF616	
IL1B		cytokine	Activated	2.837	0.000178	A4GALT,C3,CCL5,COL1A1,CXCL2,DBI,DUSP1,EGR1,FAS,FLT1,IL1RN,ITGB8,LBP,MMP13,MMP3,NFKBIZ,NR1H4,NRP1,PLA2G4A,SERPINE1,TNFAIP6,TSLP,VEGFC	118 (10)
ESR1		ligand-dependent nuclear receptor		1.402	0.000179	ABCB1,ABCG2,ABLIM1,C3,CP,CRABP2,EFEMP1,FAM102A,FAS,FOS,FRAT2,FST,GREB1,IER3,LTBP1,MAPK12,OTUB2,PROS1,PTEN,RAMP3,RGS3,SEMA3B,SERPINE1,SLC7A11,SLC7A5,STC2,TERT,TMEM74,TMEM97,TNFAIP6,TNFRSF11B,VAV3,WISP2,YPEL1	
PRKCD		kinase		-0.622	0.000183	CEMIP,COL1A1,COL1A2,FBN1,FOSL1,FZD1,IL1RN,IL2RG,LAMB3,LBH,LIPG,MMP13,NOTCH1,OAS1,SERPINE1,SPRY1,TNFRSF11B	61 (5)
NFkB (complex)		complex	Activated	2.253	0.000185	ABCB1,C3,CCL17,CCL5,CX3CL1,CXCL2,FAS,FTH1,GDF15,HLA-DMB,IER3,IL15RA,ITGB8,LSP1,MMP13,MMP3,NFKBIZ,NOTCH1,PLAU,SLC7A5,SPIB,TGM2,TNFSF10,TRIB3,TSLP,VEGFC	112 (9)
TP53		transcription regulator	Activated	2.056	0.000185	ABCB1,ACTA2,ADA,AMOTL2,AR,BCL2L11,BTG2,CCND3,CMBL,COL18A1,CYP26B1,DLX1,DRAM1,DUSP5,EGR1,EGR3,FAM212B,FAS,FDFT1,FDPS,FKBP1B,FOS,FOSL1,GADD45A,GDF15,IRS1,ITGB4,LTBP1,MAGEB1,MAPK12,MBNL2,MIR17HG,MMP3,MST1,MVD,MVK,NOTCH1,NOX4,OAS1,OSGIN1,PDE2A,PDE4B,PFKFB3,PRKCB,PTEN,PTP4A3,SEMA3C,SERPINE1,SFN,SRGAP3,SULF2,TCEA3,TERT,TMEM97,TP53INP1	122 (9)
TMPO		other		1	0.000194	ASPN,COL12A1,COL1A1,MMP15	

MAP3K7		kinase		0.707	0.000231	CCL5,CFB,IFIT3,MMP13,MMP3,OAS2,RGS2,SERPINE1,TNFSF10	121 (10)
FBN1	-2.804	other		0.692	0.000286	COL1A2,FBN1,LTBP1,MMP3,TIMP3	
SBDS		other			0.000297	CHAC1,CRABP1,CTH,EGR1,FOS,GADD45B,GAP43,OPN3,P2RX5,PCDH7,RGS4,SPP1,TRIB3	
HIF1A		transcription regulator		0.806	0.000363	BGN,CA9,CEMIP,CLDN1,CYR61,EGLN3,FOS,FSCN1,GADD45B,GJA1,INHBB,IRS2,ITGB2,KIFC2,NOX4,P4HA2,PFKFB3,SERPINE1,STC2,SULF1,TERT,TRIM66,VEGFC	
ITGAL		transmembrane receptor		0.787	0.000372	HES1,HEY1,ITGB2,NOTCH1	
IDO1		enzyme		-1.941	0.000372	SLC1A4,SLC1A5,SLC7A11,SLC7A5	
PROX1		transcription regulator			0.000372	HEY1,NRP1,PCK2,RGS4	
ERK1/2		group	Activated	2.91	0.000385	ABCA1,BCL2L11,C3,CCL5,CLEC11A,EGR1,EZR,FOS,FOSB,HBEGF,MAP3K11,MMP3,PFKFB4,TERT,TSLP,VNN1,WISP1	117 (10)
MYOC		other			0.000401	ADAMTS1,CARD10,CDH11,FBN1,FSCN1,PRKCB,PTGER4,RGS17,SLC7A11,STC2,UPP1	
Cg		complex	Activated	2.107	0.000406	AR,DMD,EGR1,FAS,FST,GATA6,ITGB3,PFKFB3,PKIA,PLAT,PLAU,RGS4,UPP1,VEGFC	
Jnk		group	Activated	2.412	0.000421	ACTA2,BCL2L11,DUSP1,FOSB,GDF15,GJA1,MAP3K11,MMP13,MMP3,PTEN,SERPINE1,TERT,TGM2	159 (13)
FOXO1		transcription regulator	Activated	2.071	0.000421	BCL2L11,EGR1,EGR2,GADD45A,GADD45B,IER3,IRS1,IRS2,MMP3,OVOL1,TNFSF10,TXNIP,WNT4	
CTNNB1		transcription regulator		0.894	0.000455	ABCB1,ALDH1A1,CDH11,CEACAM1,EPHB3,FOXQ1,ID3,INHBB,IRS1,LBH,PCCA,PDE4B,PLAU,SEMA3C,SERPINA1,SERPINE1,SFN,SLC1A5,TERT	19 (2)
MAPK14		kinase		0.686	0.000457	EFEMP1,FOS,GREM1,ITGB8,MMP13,PTEN,VDR	
Creb		group			0.000478	BDNF,DUSP1,EGR1,FOS,FOSL1,FSCN1,HES1,IER3,RGS2,WISP1	
TCF		group			0.000536	ALDH1A1,CEACAM1,EPHB3,ID3,INHBB,IRS1,PCCA,SEMA3C,SERPINA1,SERPINE1,SFN,SLC1A5	19 (2)
CD24		other		-1.546	0.000553	ADD3,CHAC1,DUSP5,EMP3,GDF15,MTUS1,PDGFB,PHF10,PLAU,PLPPR2,TFPI,TP53INP1,TUFT1,VDR	
FOS	17.107	transcription regulator		0.218	0.000594	CCL5,FTH1,GJA1,LTBP1,MMP13,MMP3,SERPINE1,SPP1,SULF2,TSLP,VAV3	51 (4)

PPP1R13L		transcription regulator		1.4	0.000614	CLDN1,DSP,GJA1,JUP,TP63	
CIP2A		other		-1.265	0.000635	CFB,CRLF1,DCN,GADD45A,IER3,PDE2A,RHOD,S100A16,SLC22A18,SNCA	
IgG		complex		0.118	0.000678	ATP1B1,CCL5,CRABP2,CYP27A1,DSP,DUSP1,EZR,FST,IFITM3,IL1RN,KRT16,KRT18,LY9,SERPINB7,TNFSF15	
NFKB1		transcription regulator	Activated	2	0.000801	CCL5,COL1A1,CXCL2,CYR61,DUSP1,FOSB,FSCN1,HLA-DMB,IL1RN,PLAU,TERT,TNFSF10,TSLP	53 (4)
PGR		ligand-dependent nuclear receptor		1.729	0.000818	ABCG2,AK4,ATP1B1,CCND3,CPT1B,EZR,FOS,HBEGF,HES1,IER3,ITGB4,KRT18,PFN2,PLAU,PRRX1,RASSF2,WNT4	41 (2)
VEGFA		growth factor	Activated	2.771	0.000839	EFNB2,FLT1,HES1,ITGB3,MMP13,NOTCH1,NRP1,PLAU	
Raf		group			0.000839	EGR1,FOSL1,GDF15,IER3,PTEN,SEMA3C,SEMA6A,SPRY1	15 (2)
EFNA2		kinase		-1.667	0.000869	BACH2,CRABP2,FOSL1,FOXL1,FOXQ1,ITGB4,KRT16,KRT18,PLAT	
GLI1		transcription regulator		1.32	0.000986	ABCG2,ARC,C11orf96,CMBL,CMPK2,COL1A1,EGR3,FAS,FGF1,MEF2C,NREP,PDZRN3,SPP1,VEGFC	
ICAM1		transmembrane receptor		1.987	0.00103	FOS,HES1,HEY1,NOTCH1	
IGFBP5		other		1	0.00103	EFNB2,IRS1,SERPINE1,SFN	22 (3)
RBPJ		transcription regulator		-1.342	0.00105	CSF1,FGF11,FOS,HES1,MMP13,PDGFB,RUNX3	26 (3)
SEL1L		other			0.00116	NOTCH1,PTEN,TUBB3	
GNE		kinase			0.00116	ASNS,CHAC1,TRIB3	
HDAC6		transcription regulator		0.115	0.00121	ADAMTS1,COL1A1,PDGFB,PLAU,SERPINE1,TGFBI	
PI3K (family)		group		0.717	0.00144	ABCA1,ACTA2,BCL2L11,FOS,FTH1,MMP3,PDGFB,PTEN,SERPINE1,SOX4,TGM2,TNFSF10,TXNIP	77 (9)
EZH2		transcription regulator		0.458	0.00148	C15orf48,C3,CCND3,CEMIP,CX3CL1,CXCL2,DAB2IP,FLT1,FZD1,GDF15,LAMB3,PCDH10,PLA2G4A,PRICKLE1,RAP1GAP,SERPINA1,SFN,SLC1A3	
MUC1		other		0.723	0.00151	ALDH1A1,CDH11,EGLN3,MRC1,PLAU,PYCR1,TERT	22 (3)

MAPK9		kinase		-1.414	0.00152	DMD,FOSL1,GADD45B,ITGB4,LMO7,MARCKSL1,NR3C2,PLA2G4A,PTEN	
SP1		transcription regulator		1.856	0.00165	ADAMTS1,ADRA1B,ADRA1D,AR,ASNS,ATP2A3,CEACAM1,COL1A1,CTH,EGR1,EZR,FLT1,FOSL1,GDF15,GJA1,HBEGF,HGF,KRT16,KRT18,LIPA,NOX4,PLAU,SPP1,TERT,TNFSF10	
IL6		cytokine		1.664	0.00173	ABCA1,ABCG2,AFP,C3,DCN,FOS,GADD45G,IL1RN,LBP,LYZ,MMP13,PDGFB,PLAU,SERPINA1,TGM2	47 (3)
TCF4		transcription regulator			0.0018	CDH17,FGF1,IRS1,PLAU,SPP1,TERT,WISP2	
SIN3A		transcription regulator		0.447	0.00186	COL1A1,COL1A2,GADD45B,SERPINE1,TERT,TXNIP	
PRNP		other		-0.346	0.00186	ABCB1,FOSL1,FST,GJA1,IRS1,TCF7L1	
IL13		cytokine		-0.321	0.002	ABCA1,ADA,BDNF,C3,CCL5,CD37,CISH,COL1A2,FAM162A,IL1RN,LIPA,MRC1,PID1,RPS6KA2,SERPINF1,SNCA,SWAP70,TGM2,TSLP	48 (4)
RELA		transcription regulator	Activated	2.704	0.00207	ABCG2,BACH2,BTG2,CCL5,COL1A1,CXCL2,DUSP1,EGR1,FAS,FOSB,FSCN1,IL1RN,PDE4B,PLAU,TGM2,TP63,TSLP,TUBB3	109 (8)
TWIST1		transcription regulator		0.956	0.00213	ALDH1A1,AR,C3,COL1A1,DCN,FAS,FMOD	
Hdac		group	Inhibited	-2.47	0.00223	ARC,EGR1,EGR3,FOS,GADD45B,JUP,SPP1,TP63,TXNIP	
TGFBR		group			0.00223	ACTA2,CDH11,NOX4	
RICTOR		other			0.00223	BCL2L11,EGR1,PTEN	14 (2)
mir-296		microrna			0.00223	CA9,CALML4,COL1A1	
CRP		other		0.843	0.00224	EGR1,PRKCB,SERPINE1,TNFSF10	101 (7)
RFX5		transcription regulator			0.00224	CD74,COL1A2,GCNT2,HLA-DMB	
SP3		transcription regulator		0.927	0.0023	ASNS,COL1A1,COL1A2,EGR1,FLT1,FOSL1,GDF15,HGF,ITGB8,KRT16,PLAU,TERT	105 (5)
IL5		cytokine		1.463	0.00243	CCND3,CEACAM1,CSF2RB,EGR2,EGR3,IER3,SLC1A5,UPP1	
Pdgfr		group			0.0025	ACTA2,EGR1	
mir-196		microrna			0.0025	COL1A1,FOS	
NKX2-5		transcription regulator			0.0025	ECE1,SYN1	

AFF1		transcription regulator			0.0025	EPHA7,PTEN	
KLB		enzyme			0.0025	EGR1,FOS	36 (4)
CTR9		other		-1.342	0.00257	EGR3,GREB1,IGFBP4,TIAM1,TP53INP1	
KRAS		enzyme		1.109	0.00278	CCND3,DUSP5,FTH1,GADD45A,IER3,LAMB3,PDE4B,UPP1	
CAPN3		peptidase	Activated	2	0.0031	CCL5,DUSP1,FTH1,NOX4	
SAA		group		1	0.0031	CCL17,IL1RN,MMP13,MRC1	101 (7)
ABL1		kinase		1	0.0031	FAS,GADD45A,IER3,TP63	83 (4)
Tgf beta		group		-0.152	0.0031	COL1A1,COL1A2,PDGFB,TSLP	
miR-21-5p (and other miRNAs w/seed AGCUUAU)		mature microrna		-1.348	0.0031	PTEN,RECK,TIMP3,TNFRSF11B	
WWTR1		transcription regulator			0.0031	CYR61,ITGB2,SERPINE1,TP63	
LYL1		transcription regulator			0.0031	BTG2,CCND3,EGR1,ID3	
CHI3L1		enzyme			0.00324	CDH11,COL6A2,NNMT,SERPINB1,SERPINE1	
SUPT16H		transcription regulator			0.00324	DUSP5,EGR1,HIST1H1C,HIST1H2BA,PLAU	
MAP2K1		kinase		1.671	0.00338	BCL2L11,DUSP1,DUSP5,FOS,FOSL1,PLA2G4A,RRAGD	14 (2)
ERN1		kinase			0.00338	ANG,GPT,OAS2,PROS1,SYT11,TINAGL1,TSPAN2	
SAFB		other		-1.238	0.0036	CCL5,CD74,CX3CL1,FOS,GULP1,MAP3K11,TNFRSF11B,TNFSF10	
AURK		group	Inhibited	-2.828	0.0036	BTG2,CMBL,CYP26B1,FAM212B,PDE2A,SRGAP3,SULF2,TCEA3	
ANLN		other	Inhibited	-2.828	0.0036	BTG2,CMBL,CYP26B1,FAM212B,PDE2A,SRGAP3,SULF2,TCEA3	
Calcineurin protein(s)		complex			0.00376	AQP5,BCL2L11,RGS2	
AMH		growth factor			0.00376	BTG2,CXCL2,IER3	53 (5)

OSMR	4.048	transmembrane receptor			0.00376	LBP,OSMR,SERPINA1	
DYRK1B		kinase			0.00376	CCND3,CP,SOD3	
PIAS3		transcription regulator			0.00376	FTH1,MMP13,MMP3	93 (6)
EDN1		cytokine			0.00376	CYR61,FST,HBEGF	
CNOT7		transcription regulator			0.00389	CLDN1,CMPK2,OAS1,OAS2,SLC14A1,SLC7A11	
MDM2		transcription regulator		0.179	0.0039	HIST2H2AA3/HIST2H2AA4,IL1RN,INHBB,LYZ,SFN,TERT,TNFSF10	
CTGF		growth factor		0.555	0.00402	EGLN3,MMP13,MMP3,SERPINE1,TIMP3	
mir-21		microrna		-0.553	0.00402	ACTA2,PTEN,RECK,TIAM1,TIMP3	
TAB1		enzyme		-1.342	0.00402	CCL5,CFB,MMP13,RGS2,TNFSF10	57 (6)
ZEB1		transcription regulator		-0.492	0.00493	ITGB4,KRT18,PLAU,SERPINE1,TP63	
BRD4		kinase		0.13	0.00497	ABLIM1,ACTA2,COL1A1,COL1A2,CXCL2,FBN1,FKBP11,FOS,KCNQ5,LOXL1,PDGFB,PLAT,PLAU	
EGR1	3.734	transcription regulator			0.00513	ACTA2,BCL2L11,COL1A2,FOSL1,GDF15,HBEGF,TNFSF10	52 (5)
MAP2K1/2		group		1.273	0.00515	BCL2L11,C3,DUSP1,EGR1,EGR2,ELN,FOS,FOSL1	23 (3)
IL17A		cytokine		0.982	0.00515	ACTA2,COL1A1,CXCL2,GADD45A,IL1RN,MMP3,MRC1,NRP1	112 (9)
ATM		kinase			0.00538	DUSP1,GADD45A,GADD45B,GADD45G,SERPINE1,TGM2	
IL1A		cytokine	Activated	2.007	0.00542	ALDH1A1,CCL5,CSF1,CXCL2,NFKBIZ,PDZK1IP1,PLAU,SERPINA1,SERPINE1,TNFRSF11B	115 (8)
COL18A1	3.205	other		-1.744	0.00542	EFNB2,FOS,HGF,ID3,ITGB2,ITGB3,NRP1,PLAU,SERPINE1	
NR3C1		ligand-dependent nuclear receptor		-1.724	0.00543	ATP1B1,BCL2L11,CARD10,CCL5,CORO2A,DNER,GADD45A,GADD45B,GULP1,IER3,IL15RA,ITGB2,MAGI3,NME5,PDE4B,PIK3CD,PLA2G4A,PRKCB,RBMS3,RGS2,RHOB,RTN4,SERPINE1,SNCA,SOX4,SPP1,TIMP3,TLR5,TNFAIP6,TNFRSF11B,TRIB3,UNC13B	
HOTAIR		other		0.881	0.00547	GDF15,LAMB3,OAS1,PCDH10	19 (2)
mir-181		microrna		-0.635	0.00547	AR,BCL2L11,GATA6,TIMP3	

HOXD10		transcription regulator		-1	0.00547	EZR,ITGB4,RHOB,SERPINE1	
mir-25		microrna			0.0058	BCL2L11,ITGB3,PTEN	
USP18		peptidase			0.0058	IFITM3,OAS1,TNFSF10	
RCOR1		transcription regulator			0.0058	COL1A1,COL1A2,SERPINE1	
KMT2D		transcription regulator		1.477	0.00597	CRIP1,LAMB3,LOXL1,PCDH7,TNNT2	
SSRP1		other			0.00597	DUSP5,EGR1,HIST1H1C,HIST1H2BA,PLAU	
LDL		complex		1.773	0.00663	ABCA1,C3,DUSP1,ECE1,PLAT,SERPINE1,VNN1	76 (8)
JUNB		transcription regulator			0.00716	FOSL1,FTH1,ITGB4,MMP13,SERPINE1	
ENTPD5		enzyme			0.00726	COL1A1,COL1A2	
MBNL1		other			0.00726	ABLIM1,PTEN	
miR-92a-3p (and other miRNAs w/seed AUUGCAC)		mature microrna			0.00726	BCL2L11,PTEN	
AMBP		transporter			0.00726	ACTA2,PLAU	
EIF2AK4		kinase			0.00726	EGR1,PCK2	
RND3		enzyme			0.00726	HES1,NOTCH1	
HIPK2		kinase			0.00726	BDNF,ITGB4	
TBPL1	-2.449	transcription regulator			0.00726	FOS,TP63	
Muscarinic cholinergic receptor		group			0.00726	FOS,RGS2	
EGLN		group		-0.818	0.00785	CA9,CSRP2,DYRK4,EGLN3,EIF4EBP1,GPR146,PEX11A,PGAM1,PGM1,RNF122,RRAGD	
CD44		other		1.51	0.00798	ABCB1,ABCG2,ACTA2,CRABP2,FAS,MBNL3,PLAU,SBK1,SERPINA1	83 (6)
FAT1		other			0.00837	MMP3,PLAU,VEGFC	

mir-19		microrna			0.00837	ABCA1,BCL2L11,PTEN	
UBE3A		enzyme			0.00837	ARC,TERT,TP53INP1	
SPARC		other			0.00837	HES1,NOTCH1,SERPINE1	
MNT		transcription regulator			0.00837	GADD45B,TERT,TXNIP	
ELK1		transcription regulator		0.447	0.00851	FOSL1,MMP13,MTHFD2,SLC7A11,SPP1	
LMNA		other			0.00881	COL12A1,MMP3,PTEN,TIMP3	
SIN3B		transcription regulator			0.00881	COL1A2,GADD45B,HES1,TXNIP	
E2F3		transcription regulator		0.508	0.00898	CA12,ECE1,FAM102A,FST,HIST1H1C,HIST1H2BJ,HOXB9,KRT80,MIR17HG,RBPMS,TERT,TIMP3	
CEBPA		transcription regulator		-0.632	0.00927	ASNS,BTG2,C3,GJA1,IL1RN,OAS2,PFN2,RGS2,SERPINB1,SERPINF1,SPP1,TNFSF10,VDR	
PAF1		other		1.89	0.00945	CCL5,IFIT3,IFITM3,LRRN3,NFKBIZ,OAS2,SERPINE1	
IGFBP2		other		-0.883	0.00953	DDAH1,EFEMP1,GDF15,POSTN,PTEN,RBMS3	
USF1		transcription regulator			0.00953	APLN,CEACAM1,SERPINE1,SPP1,TERT,TXNIP	
TAL1	28.621	transcription regulator		-1.585	0.00974	ARVCF,C3,CHST11,DNMBP,FBN1,GDF5,HIVEP3,LRRN3,MCTP1,MVD,NEFL,NFKBIZ,NOTCH1,OXR1,PTGER4,SOX4,TNFSF10,TSPAN7	
MGEA5		enzyme		0	0.00989	ABLIM1,ADAM19,CEACAM1,CLDN1,EFNB1,EFNB2,EPHB3,FDFT1,FDPS,FSCN1,FZD6,GADD45A,GALK2,GPT2,IGFBP4,PLAU,RAB27A,RHOB,TGDS,TIMP3	
SMAD2		transcription regulator		0	0.01	ACTA2,BCL2L11,SERPINE1,TIMP3,VEGFC	
Pkc(s)		group		1.857	0.0107	BCL2L11,FOS,FOSL1,GADD45A,GADD45B,LIPA,OVOL1,RGS2	
IL1		group		0.9	0.0109	FOS,MMP13,MMP3,TNFRSF11B	
FOXO4		transcription regulator		-0.064	0.0109	GADD45A,GADD45B,IER3,OVOL1	

miR-100-5p (and other miRNAs w/seed ACCCGUA)		mature microrna			0.0115	CCND3,EGR2,MMP13	
mir-203		microrna			0.0115	HES1,LIFR,TP63	
CSF2		cytokine		1.929	0.0115	CCND3,CEACAM1,CSF2RB,EGR2,EGR3,FLT1,IER3,SLC1A5,TLR5,UPP1	
NRG1		growth factor		0.031	0.0117	ABCA1,CYR61,FOS,ITGB3,VEGFC	
BCL6		transcription regulator			0.0118	ALCAM,CISH,COL1A1,FMOD,FTH1,FUT7,GADD45A,SERPINE1	
WISP2	175.782	growth factor		-0.128	0.0123	CLDN1,DSP,JUP,KRT18,LAMB3,SERPINE1	
FOXL2		transcription regulator		1.01	0.0131	CXCL2,FOS,IER3,RGS2,RSPO3,SOX4,SPRY1	
TFAP2A		transcription regulator			0.0131	ABCA1,ALCAM,CXCL2,ITGBL1,PLAU,TERT,WISP2	
HGF	-4.034	growth factor		1.754	0.0131	CA9,CCL5,EFNB2,EGR1,FOS,PGAM1,PLAU,RHOB,TMEM97,VEGFC,WT1	
CD3		complex		-0.06	0.0131	CCL17,CCND3,CD74,EGR2,FAS,IL2RG,NDFIP2,ORAI3,PDE4B,PTGER4,TNFSF10	
TGFBR1		kinase		1.165	0.0132	DOCK4,PDGFB,PTPRK,VEGFC	
DCAF1		kinase		0	0.0132	OPN3,TGM2,TNFSF10,TXNIP	
Smad2/3		group			0.0132	ABCG2,SERPINE1,TGM2,TIMP3	
PDLIM2		other		0	0.0134	BST1,CEMIP,FHDC1,GNG2,GSTM2,HBEGF,IFIT3,RASGEF1A,STC2,TXNIP	
HDAC1		transcription regulator		-0.447	0.0134	COL1A1,COL1A2,DAB2IP,ITGB4,PTEN,RHOB,SERPINE1,SPP1,TERT	
MAP3K14		kinase		0.447	0.0136	CCL5,FAS,GADD45A,PLA2G4A,TNFAIP6	
EFNA5	3.182	kinase		-1.633	0.0139	CRABP2,FOSL1,ITGB4,KRT16,KRT18,PLAT	
FANCA		other			0.014	EGR1,HES1	
GDF15	-8.448	growth factor			0.014	GDF15,PLAU	

miR-516a-3p (and other miRNAs w/seed GCUUCCU)		mature microrna			0.014	AR,SULF1	
miR-19b-3p (and other miRNAs w/seed GUGCAAA)		mature microrna			0.014	BCL2L11,PTEN	
mir-32		microrna			0.014	BCL2L11,BTG2	
NR1H2		ligand-dependent nuclear receptor			0.014	ABCA1,C3	
MAPK12	-3.066	kinase			0.014	ITGB8,MAPK12	
PTH		other			0.014	CSF1,FOS	
CANX		other			0.014	ABCA1,NOX4	
ISG15		other			0.014	IFITM3,OAS1	
HBEGF	2.616	growth factor			0.014	AR,NRP1	
TNFAIP6	19.953	other			0.014	ACTA2,TNFAIP6	
mir-148		microrna			0.0153	ALCAM,EGR1,MMP15	
ENG		transmembrane receptor			0.0153	ITGB3,PLAU,SERPINE1	
JMJD1C		enzyme			0.0153	EGR1,FLT1,TAL1	
POU2F2		transcription regulator		-0.378	0.0155	GATM,MVK,PFKFB3,PFKFB4,PRR7,SPP1,SYDE2,TERT	
CXCL8		cytokine		-0.64	0.0156	ABCG2,AR,CD74,COL12A1,COL18A1,ITGB2	
OSM		cytokine	Activated	2.211	0.0156	FOS,LBP,LIFR,OSMR,SERPINA1	

ITGB1		transmembrane receptor		0.662	0.0156	COL1A1,EIF4EBP1,FOS,ITGB3,PLAU		
JUND		transcription regulator			0.0159	FOSL1,FTH1,ITGB4,MMP13		
EFNA1		other		-1.89	0.016	BACH2,CRABP2,FOSL1,GATA6,ITGB4,KRT18,PLAT		
PPARG		ligand-dependent nuclear receptor		0.38	0.0169	ABCG2,ACTA2,COL1A1,COL1A2,GPT,PTEN,SERPINE1,TNFSF10		
HMOX1		enzyme		1.455	0.0174	FGF1,HGF,ID3,IL1RN,ITGB3,MAPT		
EFNA4		kinase		-1.633	0.0174	CRABP2,FOSL1,ITGB4,KRT16,KRT18,PLAT		
EFNA3		kinase		-1.633	0.0174	CRABP2,FOSL1,ITGB4,KRT16,KRT18,PLAT		
MYOCD		transcription regulator		1.821	0.0176	COL1A1,COL1A2,DES,GJA1,LMOD1,MEF2C,TNNT2		
Secretase gamma		complex		1.216	0.0189	HES1,HEY1,NOTCH1,PTPRF		
Mapk		group			0.0189	FOS,IRS1,MMP13,MMP3		
SNAI2		transcription regulator			0.0189	ITGB4,JUP,TP63,VDR		
PTGS2		enzyme			0.0193	CCL5,EGR1,EZR,FLT1,ITGB4,TNFSF10,VNN1		
BCOR		transcription regulator			0.0196	CD37,CD74,LSP1		
mir-30		microrna			0.0196	AR,BCL2L11,GADD45A		
PAEP		other			0.0196	CCL5,CXCL16,HBEGF		
PRL		cytokine		1	0.0197	CISH,CMPK2,DHX58,GMPR,IFIT3,LY6E,OAS1,OAS2,TRIM14		
BMP4		growth factor		0.574	0.0197	ABCG2,ACTA2,HEY1,OVOL1,PAPPA2,RUNX3,SPNS2,TGM2,TRPV2		
GAPDH		enzyme		-0.447	0.0204	C2,DUSP1,OAS1,OAS2,SPP1		
HDAC2		transcription regulator			0.0204	COL1A2,ITGB8,RHOB,TERT,TP63		
DICER1		enzyme			0.0212	COL18A1,CYR61,GNG2,ID3,LIPG,PLAU,PROS1,SERPINA1,SERPINF1		
BMP2		growth factor		0.651	0.0222	GADD45B,RUNX3,SPP1,WNT4		

ATF2		transcription regulator			0.0222	ASNS,DUSP1,ITGB8,PTEN		
KDM3A		transcription regulator			0.0226	GDF15,SERPINE1		
NOTCH4		transcription regulator			0.0226	ACTA2,HEY1		
KCNN4		ion channel			0.0226	CCL5,CX3CL1		
PHLPP1		enzyme			0.0226	IRS1,PRKCB		
CTBP2		transcription regulator			0.0226	NEFL,PTEN		
ZBTB7B		transcription regulator			0.0226	COL1A1,COL1A2		
IL2		cytokine		0.728	0.024	CARD10,CCND3,CISH,CSF2RB,FAS,GADD45B,GADD45G,IER3,IL2RG,NETO2,PDE4B,RHOB,SPP1,TNFRSF11B,TNFSF10,TP53INP1,TRIB3,WT1		
IFNA2		cytokine		1.238	0.0244	CISH,FAS,IFIT3,IFITM3,LY6E,OAS1,OAS2,TNFSF10,TRIM14		
MDGA2		other			0.0245	FAS,TP63,WT1		
TSC22D3		transcription regulator			0.0245	BCL2L11,CCL5,DUSP1		
PIK3R1		kinase			0.0245	AR,PIK3CD,PLAU		
FOSL2		transcription regulator			0.0245	COL1A2,FOSL1,ITGB4		
PDCD4		other			0.0245	MMP3,PLAU,VEGFC		
IRF9		transcription regulator			0.0245	IFIT3,IFITM3,OAS2		
STAT1		transcription regulator		1.738	0.0247	C1S,C4A/C4B,CX3CL1,FAS,GBP5,IFIT3,LRRTM2,NOX4,OAS1,OAS2,TNFSF10		
SPDEF		transcription regulator		-1.342	0.0251	CDH11,COL1A1,COL4A6,COL6A2,PLAU,PTPRF,SERPINE1		
TGM2	-2.477	enzyme	Activated	2.668	0.0255	BTG2,C3,CD74,CEACAM1,GJA1,IFIT3,ITGB3,KCNQ5,LSP1,LY6E,MIR17HG,OAS1,OAS2,SIRPA,SLC16A13,SLPI,SPP1		
CDK9		kinase			0.0258	CCL5,CXCL2,FOS,FRAT2		
TRAP1		enzyme		1.342	0.0259	AK3,ALDH1L2,GARS,GPT2,MTHFD2		
PTEN	2.453	phosphatase		-1.009	0.0259	AR,BCL2L11,CCL5,FTH1,PTEN		
H2AFY		other		-1.342	0.0259	EGR1,GADD45A,PLAU,SERPINE1,TERT		

INHBA		growth factor		0.711	0.0281	ACTA2,CCL17,CPNE8,DTNA,EFNB2,GULP1,PTEN,SOX4,TAL1,VAV3		
Mek		group		-0.018	0.0288	BCL2L11,EGR1,FOSL1,GDF15,IER3,MMP13,NOX4,PTEN,SEMA3C,SEMA6A,SERPINE1,SPRY1,VEGFC		
BCL2L1		other		-0.447	0.029	COL6A2,FAS,TNFRSF11B,TNFSF10,TUBB3		
HIC1		transcription regulator		0.378	0.0297	ACTA2,CA12,FST,KAZN,PLA2G4A,SLC7A11,SPP1		
CREB1		transcription regulator			0.0297	CYR61,FLT1,FOSB,FUT7,MMP13,PLA2G4A,RGS2		
mir-15		microrna		0.165	0.0298	AR,CCL5,OAS1,RECK		
SFN	3.3	other			0.0298	BTG2,DSP,JUP,KRT18		
PTP4A3	2.818	phosphatase			0.0301	ITGB3,JUP,PTEN		
ITGB3	14.022	transmembrane receptor			0.0301	CEACAM1,COL1A1,ITGB3		
IFNG		cytokine	Activated	2.811	0.0312	BCL2L11,CCL17,CCL5,CEACAM1,CHAC1,CISH,CP,CX3CL1,CXCL16,CXCL2,FAS,IFIT3,IGFBP4,IL1RN,ITGB3,MARCKSL1,MMP13,OAS1,OAS2,PARVG,TLR5,TNFSF10,TRIB2,TXNIP		
SRF		transcription regulator			0.0318	DUSP5,EGR1,EGR2,FGF1,FOS,FOSB,FOSL1,GADD45G,RAI2		
NEDD9		other		1.342	0.0324	CA9,FOS,GDF15,SERPINE1,TXNIP		
TBX5		transcription regulator		1.283	0.0324	COL1A1,COL1A2,DES,GJA1,TNNT2		
HAND2		transcription regulator		1.281	0.0324	COL1A1,COL1A2,DES,GJA1,TNNT2		
CUX1		transcription regulator			0.0324	CCL5,ITGB2,PCDH10,WISP2,WNT10A		
PADI4		enzyme			0.0328	GADD45A,OSGIN1		
PHLPP2		enzyme			0.0328	IRS1,PRKCB		
SMYD3		enzyme			0.0328	GREB1,TERT		
HIF1AN		enzyme			0.0328	CA9,EGLN3		
AGR2		other			0.0328	EGR1,FOS		
BHLHE40		transcription regulator			0.0328	MEF2C,TP63		
AKT3		kinase			0.0328	BCL2L11,FOSL1		

PDE4B	28.271	enzyme			0.0328	CCL5,DUSP1		
MAP2K7		kinase			0.0328	CCL5,MMP3		
PTPRR		phosphatase			0.0328	FOS,FOSL1		
DPP4		peptidase			0.0328	EGR2,IL1RN		
SKI		transcription regulator			0.0328	ACTA2,SERPINE1		
TNFRSF12A		transmembrane receptor			0.0328	CCL5,MMP3		
INSR		kinase			0.0328	EGR1,FOS		
CD99		other			0.0328	FOSB,TUBB3		
GSK3A		kinase			0.0328	IRS1,TNFSF10		
NAB2		transcription regulator			0.0328	FLT1,PLAU		
BHLHE41		transcription regulator			0.0328	CA9,MEF2C		
SF1		transcription regulator			0.0328	ACTA2,COL1A1		
SUZ12		enzyme			0.0339	ANXA8/ANXA8L1,DAB2IP,LAMB3,PCDH10,PLAU,PLCB4,PTEN,SLC7A5,TNFRSF11B		
Hsp27		group		-0.152	0.0341	CSF1,EIF4EBP1,FOS,NOX4		
ANXA2		other		-0.849	0.0341	ANG,FAS,GADD45A,PTEN		
MAPK1		kinase		-0.671	0.0356	AR,C1S,CCL5,CFB,CRYL1,DUSP1,FOS,IFIT3,IFITM3,OAS1,OAS2,PLA2G4A,TNFSF10,TRIM14,VDR		
MAX		transcription regulator			0.036	GADD45A,GADD45B,LRRN3,TERT,TXNIP		
KRT14		other			0.036	GJA1,KLK10,KRT16,KRT18,MMP13		
F2R		g-protein coupled receptor			0.0363	BCL2L11,CYR61,GJA1		
mir-17		microrna			0.0363	AR,BCL2L11,FAS		
SMARCD3		transcription regulator			0.0363	ALCAM,COL1A1,FMOD		
CARM1		transcription regulator			0.0363	EGR3,FTH1,IGFBP4		
CD46		other			0.0363	C3,PTGER4,SLC7A5		

CEACAM 1	11.152	transporter			0.0363	ALDH1A1,HEY1,VEGFC	
TBXT		transcription regulator			0.0363	ALCAM,COL1A1,FMOD	
CDK4		kinase			0.0367	CPEB1,ELN,LRRTM2,MMP3,MORC4,PBLD,RBMS3,RGS2,TM7SF2,TMEM17,TP53INP1,ZNF423	
STAT3		transcription regulator		1.706	0.0371	ACTA2,CA9,CEACAM1,COL1A1,EGR2,EGR3,FOS,FSCN1,HIST2H2AA3/HIST2H2AA4,IL1RN,LBP,NOTCH1,SERPINE1,STC2,TERT	
EGF		growth factor	Activated	2.585	0.0374	CCL17,DUSP1,DUSP5,EGR1,EZR,FOS,SERPINE1	
FANCC		other			0.0378	ATP1B1,GDF15,HES1,MTUS2,OAS1,PRKCB	
Akt		group		0.994	0.0383	ABCB1,ACTA2,BTG2,CCND3,FOS,FOSL1,NOTCH1,SERPINE1,SOX4	
SATB1		transcription regulator		-1.941	0.0383	CEACAM1,GADD45B,GNG4,GPT2,HLA-DMB,HVCN1,LRRN3,PRKCB,SIPA1L2	
REST		transcription regulator		0.919	0.0388	GAP43,NRP1,SYN1,TUBB3	
mir-29		microrna		-1.951	0.0388	BDKRB2,CLDN1,ITGA11,PTEN	
KDM1A		enzyme		-0.555	0.0398	COL1A1,COL1A2,KRT18,PRICKLE1,SERPINE1	
TFRC		transporter		-1	0.0398	FOS,GADD45A,ITGB2,SULF2,TNFSF10	
EP300		transcription regulator			0.041	AR,CA12,GREB1,ID3,ITGB2,PLA2G4A,PLA2G7,TERT	
EGFR		kinase		0.132	0.0419	ABCG2,ACTA2,AR,BCL2L11,EGR1,FOS,PLAU,POSTN,SLC7A11,TGM2	
Interferon alpha		group		-0.524	0.0425	A4GALT,ADAM19,BTG2,CCND3,CSF2RB,CTH,DHX58,FOS,IFIT3,IGFBP4,OAS1,OAS2,PARVG,SERPINB1,SLC1A5,SPRY1,TNFSF10,TRIB2	
PIK3CA		kinase			0.043	IRS2,PDGFB,PIK3CD	
SPHK1		kinase			0.043	CCL17,CCL5,SERPINE1	
ATF1		transcription regulator			0.043	FOSL1,FTH1,FUT7	
ELF4		transcription regulator			0.043	ABCB1,CXCL2,MAPKAPK3	
ADORA3		g-protein coupled receptor			0.043	CCL5,CSF1,SPP1	

PRKCA		kinase		-0.369	0.0433	EGR1,HES1,HEY1,IFIT3,MMP3,PTEN,SERPINE1	
YAP1		transcription regulator		0.865	0.0438	AMOTL2,BCL2L11,CYR61,PTEN,TP63	
CREBBP		transcription regulator			0.0438	CYR61,FOSB,FUT7,RGS2,TNFSF10	
ARID3B		transcription regulator			0.0445	BCL2L11,TNFSF10	
ZNF100		other			0.0445	DDAH1,POSTN	
ZNF85		transcription regulator			0.0445	DDAH1,POSTN	
ZNF254		other			0.0445	DDAH1,POSTN	
RASSF8		other			0.0445	DDAH1,POSTN	
ZNF431		other			0.0445	DDAH1,POSTN	
CNOT6L		enzyme			0.0445	PTEN,SEMA3E	
IGF2R		transmembrane receptor			0.0445	DDAH1,POSTN	
MIA		other			0.0445	ITGB3,PLAT	
AGT		growth factor			0.0445	COL1A1,EGR1	
U1 snRNP		complex			0.0445	CCL5,LY9	
HES1	31.466	transcription regulator			0.0445	DUSP1,HES1	
NUMB		other			0.0445	HES1,HEY1	
miR-221-3p (and other miRNAs w/seed GCUACAU)		mature microrna			0.0445	PTEN,TIMP3	
SMARCA1		transcription regulator			0.0445	GADD45A,SFN	
mir-154		microrna			0.0445	BCL2L11,PTEN	
ZNF665		other			0.0445	DDAH1,POSTN	
ZNF528		other			0.0445	DDAH1,POSTN	

ZNF43		other			0.0445	DDAH1,POSTN		
ZNF429		other			0.0445	DDAH1,POSTN		
VIM		other			0.0445	ITGB4,PLAU		
PIK3CB		kinase			0.0445	APLN,AR		
RPS6KA3		kinase			0.0445	FOS,FSCN1		
CDK2		kinase			0.0445	FOS,TERT		
ZNF91		transcription regulator			0.0445	DDAH1,POSTN		
ZNF708		other			0.0445	DDAH1,POSTN		
CEBPB		transcription regulator		-0.152	0.0466	ABCB1,ALDH1A1,ASNS,C3,CCL5,GADD45A,KRT18,MMP3		
TLR7/8		group		0.277	0.0481	NFKBIZ,SLC7A11,TNFAIP6,TRIB3,VDR		
NFE2L2		transcription regulator			0.0481	FTH1,IL1RN,OSGIN1,SERPINE1,SHMT2		
EHF		transcription regulator		1.89	0.0531	CRABP2,IL1RN,PLAT,SERPINE1,TFPI,VEGFC,VNN1		
TLR7		transmembr ane receptor		1.97	0.059	CXCL2,GNG2,IER3,IFIT3,NR1H4,OAS2,PLAT,PLAU		
JAG2		growth factor		-1.982	0.0672	C3,CCL5,IL1RN,SPP1		
IL18		cytokine		1.832	0.0788	FAS,MMP13,MMP15,MMP3,NFKBIZ,PTEN		
GNA12		enzyme	Activated	2.236	0.0847	EZR,IRS1,LAMB3,PIK3CD,VAV3		
AREG		growth factor		-1.969	0.0946	C3,HIST2H2AA3/HIST2H2AA4,IFIT3,ITGB8,MMP15,PLAU		
CDH1		other		-1.969	0.112	CCL17,EIF4EBP1,MMP3,TSLP		
IRF3		transcription regulator		1.982	0.138	CCL5,CX3CL1,MMP3,TSLP		
RABL6		other	Inhibited	-2.236	0.225	BTG2,CX3CL1,DRAM1,HBEGF,TMEM97		
LONP1		peptidase	Activated	2	0.235	ALDH1L2,GARS,GPT2,MTHFD2,PGAM1		
MTOR		kinase		1.929	0.274	AR,CXCL2,MAPT,SOX4		
TLR9		transmembr ane receptor	Activated	2	0.285	CCL5,IFIT3,NR1H4,OAS2		
