# Supplementary material for: The impact and outcomes of cancer-macrophage fusion
Source: BMC Cancer. 2023 Jun 1;23:497. doi: 10.1186/s12885-023-10961-9 (PMC10236829; doi:10.1186/s12885-023-10961-9)
Supplement: Supplementary file 4 — Supplementary Material 4 [file 12885_2023_10961_MOESM4_ESM.pdf]

Supporting information

S2 Table. Upstream regulatory analysis of D3 hybrid cells vs. SCCVII/SF-GFP. The RNA-seq data analyzed by Ingenuity Pathway Analysis (QIAGEN).

© 2000-2018 QIAGEN. All rights reserved.							
Upstream Regulator	Expr Fold Change	Molecule Type	Predicted Activation State	Activation z-score	p-value of overlap	Target molecules in dataset	Mechanistic Network
TGFB1		growth factor		1.538	1.44E-15	ACTA2,AFP,ARHGEF2,ASPN,BARD1,BCL2L11,BGN,BIRC5,BRIP1,CCNA2,CCNB1,CDH11,CDH2,CDT1,CKS2,COL18A1,COL1A1,COL1A2,COL5A1,COL6A3,CTGF,DOCK4,EDN1,ESR2,FAS,FLT1,FN1,FOS,FOXO1,FSCN1,FSTL3,GLI1,HEY1,IGF1,IL1RL1,IRS1,ITGA1,ITGA3,ITGA6,ITGB2,ITGB3,KDM5B,LAMC2,LTBP2,MMP13,MMP2,MMP3,MRC1,MXD3,MYBL2,NET1,NRP1,PIK3CD,PLAU,PLOD2,PRIM1,PTGS2,PTPRK,RAD51AP1,RHOB,SCD,SERPINE1,SIX1,SNAI1,TAGLN,TBX2,TERT,TGFA,TGM2,THPO,TIMP1,TIMP3,TOP2A,TWIST2,VCAN,WNT5B	270 (18)
ERBB2		kinase	Inhibited	-2.451	1.67E-15	AR,BARD1,BIRC5,BRIP1,CCNA2,CCNB1,CDC25B,CDC42BPG,CDC45,CDC6,CDH11,CDH2,CDT1,CKS2,COL18A1,COL1A1,COL5A1,COL6A3,CTGF,CXCL10,DHFR,E2F2,E2F7,E2F8,EDN1,FN1,FOS,FOXO1,FSCN1,FSTL3,GINS1,GINS2,GINS4,HEY1,IL6R,KDM5B,LAMC2,LTBP2,MCM10,MCM2,MCM3,MCM5,MCM7,MXD3,MYBL2,NET1,ORC2,ORC6,PGK1,PIK3CD,PLAU,POLD2,POLE,PRIM1,PRIM2,PTGS2,PTPRK,RAD51AP1,RFC3,RPA2,RRM2,SERPINE1,SHMT1,TAGLN,TK1,TOP2A,VEGFC,WNT5B	238 (19)
TP53		transcription regulator	Activated	2.928	1.34E-12	ABCB1,ACSL3,ACTA2,ADA,AKAP12,AMOTL2,AR,ARHGEF2,ATF3,AURKB,AXIN2,BCL2L11,BIRC5,BMF,BNIP3,BRCA1,BTG2,CCNA2,CCNB1,CCNG1,CDC25B,CDC6,CDT1,CERS6,CHEK1,CHIC1,COL18A1,CPOX,CTGF,CYFIP2,CYP26B1,CYP51A1,DAPK1,DBP,DDIT3,DEK,DHFR,DUSP5,DUT,FAM212B,FAS,FDFT1,FDPS,FKBP1B,FOS,FOXO1,FUBP1,GADD45A,GDF15,HMGCR,HMGCS1,HSPA4L,IGF1,IL7,IRS1,ISG15,ITGB4,LSS,MAD2L1,MAP2K6,MCM2,MCM3,MCM7,MMP2,MMP3,MVD,MVK,NME1,NOTCH1,NOX4,OAS1,OSGIN1,PARD6B,PBK,PDE2A,PDE4B,PDK1,PHGDH,PHLDB3,PIDD1,POLD2,PRIM1,PRKD1,PTEN,PTP4A1,RAD23A,RECQL4,RFC3,RPS6KA1,RRM2,SEMA3C,SERPINE1,SMAD6,SNAI1,SORBS1,SOX2,SQLE,SRGAP3,STMN1,SULF2,TAGLN2,TCEA3,TERT,TGFA,TMEM97,TOP2A,TP53INP1,UIMC1,UNC5B,VCAN	269 (15)

TNF		cytokine	Activated	2.018	1.85E-11	ACTA2,ALDH2,BIRC5,BTG2,C3,CCL17,CDH11,CDH2,CHST2,COL1A2,CSF1,CX3CL1,CXCL10,CXCL16,DUSP5,EDN1,F2RL1,F3,FAS,FGFR2,FLT1,FN1,FOS,GADD45A,GCH1,GPR176,HDAC9,HIVEP1,IFIT3,IGF2,IL15,IL1R1,IL4R,INHBA,ITGB3,LAMB3,LAMC2,LSS,LTBP2,LYN,MARCKSL1,MCAM,MCM3,MMP13,MMP2,MMP3,NGF,NOTCH1,NR1H4,NR4A2,OAS1,OSMR,PHGDH,PIK3CD,PKMYT1,PLAU,PLOD2,PRSS23,PTGS2,RBPMS,RFTN1,RGS2,RHOB,RND3,RRM2,SEMA3C,SERPINB9,SERPINE1,SLC7A1,SNAI1,SQLE,TERT,TGFA,TIMP1,TIMP3,TLR2,TM4SF1,TNFAIP2,TNFRSF11B,TNFSF10,USP2,VEGFC,WISP1	282 (19)
TRIB3	-17.457	kinase	Activated	3.416	3.6E-11	ASNS,CTH,DDIT3,GARS,GDF15,HERPUD1,MTHFD2,PCK2,PSAT1,PSPH,STC2,TRIB3	
E2F4		transcription regulator			3.89E-10	ASF1B,ATRX,AURKB,BARD1,BRCA1,CCNA2,CDC6,CHEK1,CKS2,DHFR,DUT,E2F2,FDFT1,GINS1,H2AFZ,HIST1H2AC,MAD2L1,MCM10,MCM3,MCM5,MYBL2,PRIM2,PSAT1,RAD51,RAD51AP1,RAD54L,RBBP8,RBL1,RFC3,RRM2,SLC3A2,TEAD4,TERT,TK1,TMPO,TOP2A,TP53INP1,UNG,UXT	
SMAD3		transcription regulator		1.874	2.62E-09	ACTA2,ASPN,C3,COL1A1,COL1A2,DEPTOR,DOCK4,FN1,FSTL3,GLI1,HEY1,MMP13,NET1,PTGS2,PTPRK,RHOB,SERPINE1,SNAI1,SOX2,TERT,TIMP3,VEGFC	106 (7)
estrogen receptor		group		-1.311	6.84E-09	ABCA1,ABCG1,C3,CA12,CD68,CDH11,COL12A1,COL4A1,COL4A2,COL4A6,COL5A1,COL6A2,CTGF,CXCL12,DSP,EDN1,FGF1,FGFR2,FGFR3,FN1,FOS,GREB1,ITGA3,KRT18,LAMC2,LYN,MAPT,MMP15,PCDH10,PCDHB14,PDGFC,PLAU,RGS2,SERPINE1,SMAD6,TERT,TGFA,TGFB2,TGFB3,TIMP1,TIMP3,VEGFC,WNT5B	
AR	-791.064	ligand-dependent nuclear receptor		0.815	1.62E-08	ABCA1,ABCG1,AR,BIRC5,CCNA2,CDC25B,CDH2,CDT1,CHTF18,CXCL12,EBP,ENO2,GDF15,HGF,HMGCR,IGF1,IL1R1,ITGA6,ITGB4,KDM4B,KNTC1,KPNA2,LMOD1,LRIG1,MAD2L1,MAOA,MCM2,MSMO1,MTHFD2,MYOM1,NUPR1,PDIA5,PKMYT1,PLOD2,PROS1,RHOB,SERPINE1,SLC7A11,TGFB2,TUBB3,VCAN	281 (19)
MITF		transcription regulator	Inhibited	-2.502	2.33E-08	ALCAM,ATRX,AURKB,BRCA1,CCNB1,CDC25B,CDT1,CENPH,CENPM,CHTF18,COL1A1,FANCA,FMOD,FOS,ITGA3,MCM2,MCM5,ORC6,PSMC3IP,RECQL4,SEMA3C,SERPINE1,SOX5,SPAG5,TERT,TXNIP,UIMC1,UPP1	

ATF4	-3.996	transcription regulator	Inhibited	-3.369	3.07E-08	ASNS,ATF3,CA9,CHAC1,DDIT3,PCK2,PHGDH,PSAT1,PSPH,PTGS2,SLC1A4,SLC1A5,SLC7A11	
Mek		group	Inhibited	-2.715	5.98E-08	ABCE1,BCL2L11,BIRC5,BMF,CXCL10,DDR1,DDX21,DSCC1,ETV5,GDF15,GPER1,ITGA6,LRP8,MAF,MMP13,MMP2,NGF,NOL3,NOX4,POLR3G,PTEN,PTGS2,RABGGTB,RND3,SEMA3C,SEMA6A,SERPINE1,SLC20A1,SPRY1,SPRY4,STON1,UNG,VEGFC	133 (13)
Cg		complex		-1.071	8.31E-08	AR,BARD1,CDC6,CDH2,ESR2,F2RL1,FAS,GATA6,IL33,ITGA1,ITGB3,MAF,MCAM,MCM10,MMP2,PLAT,PLAU,PTGS2,RECQL,SLC20A1,SLC4A4,TIPIN,TM4SF1,UNG,UPP1,VEGFC	116 (6)
HDAC1		transcription regulator		-1.633	1.26E-07	AXIN2,BRCA2,CCNB1,CDC6,COL1A1,COL1A2,DAB2IP,DHFR,FOXM1,GSTP1,ITGB4,PREX1,PTEN,PTGS2,RBL1,RGS10,RHOB,RRM2,SERPINE1,SPP1,TERT,TOP2A	241 (12)
E2F1		transcription regulator		1.612	1.27E-07	AR,AURKB,BCL2L11,BIRC5,BRCA1,CCNA2,CCNB1,CDC6,CWC27,DHFR,DUT,EYA4,FAS,FGFR2,FLT1,GINS1,HIST1H2AC,IGF2,LACTB,MAF,MCM10,MCM3,MCM5,MYBL2,PRIM2,PSAT1,RAD51,RAD54L,RBBP8,RBL1,RFC3,RRM2,SLC3A2,TERT,TK1,TOP2A,TP53INP1,UNG,UXT	237 (10)
E2F6		transcription regulator	Activated	3	1.32E-07	BRCA1,CDC45,CDC6,DHPS,E2F2,EFNA5,GINS2,GMNN,MCM2,MCM3,MCM5,RAD51,RAD51AP1,RBBP8,RECQL,RRM2,UXT	
ESR1		ligand-dependent nuclear receptor		-0.143	6.1E-07	ABCB1,ABLIM1,BIRC5,BRCA1,C3,CCNA2,CENPM,CENPN,CENPU,CPE,CXCL12,DDX21,DUSP9,EDN1,EIF5A2,FAM102A,FAS,FOS,FOXM1,GPER1,GREB1,IGF1,ITGA6,KDM4B,MAD2L1,OTUB2,PBK,PDCD4,PLK4,PLXNA2,PROS1,PRSS23,PTEN,PTGS2,RAMP3,SCUBE2,SEMA3B,SERPINE1,SHANK3,SIAH2,SLC7A11,SLC7A5,SMAD6,SNAI1,STC2,TERT,TGFA,TM4SF1,TMEM74,TMEM97,TNFAIP2,TNFRSF11B,TSKU,VAV3,WISP2,YPEL1,ZNF367	193 (17)
RABL6		other	Inhibited	-4.359	7.62E-07	AURKB,BTG2,CCNA2,CCNB1,CHEK1,CX3CL1,DAPK1,DUT,MAD2L1,MCM10,MCM2,MCM5,MCM7,PBK,PRIM1,RFC3,SMAD6,TMEM97,TOP2A	179 (7)
NEUROG1		transcription regulator		-0.471	9.71E-07	ADD3,AMIGO2,C1S,C3,CEMIP,CFH,DSP,FAM198B,FN1,GREM1,INHBA,ITGB3,LRIG1,LRRN3,P4HA2,PXDN,TACR1,TMTC2	

SP1		transcription regulator		-0.821	9.81E-07	ACVRL1,ADAMTS1,ADRA1B,ADRA1D,AR,ASNS,ATF3,ATP2A3,BIRC5,CDC42BPG,CDH2,CEBPD,COL1A1,CTH,CXCL10,CYP51A1,DLC1,EZR,FLT1,FN1,FOXM1,GDF15,HGF,HINT1,HMGA1,IGF2,IL15,IL2RB,KRT16,KRT18,LIPA,MAOA,MAOB,MMP2,NOX4,PDK1,PLAU,PREX1,PTGS2,RBL1,SLC22A4,SPP1,TERT,TGFB2,TLR2,TNFSF10	173 (8)
HNRNPA2B1		other			1.08E-06	ABCB1,ARSJ,ATRX,CA12,CACNB2,CEMIP,CLDN1,CRIP1,DDR1,ELOVL7,EYA4,FHL1,FN1,FSCN1,GDF15,HYAL1,INHBB,NR2F1,OAS1,PBX1,PDE1A,PDGFC,PLOD2,SEMA3B,SNAI1,STK32B,SYT1,TGFB2,VCAN,WNT4	
PRKCD		kinase		0.897	1.14E-06	BIRC5,CEMIP,COL1A1,COL1A2,CXCL10,CXCL12,DENND3,FZD1,GEM,GLI1,GPRC5A,IL2RG,KLF5,LAMB3,LBH,MMP13,MMP2,MSI1,NOTCH1,OAS1,OSR2,PDCD4,SERPINE1,SMAD6,SOX2,SPRY1,TLR2,TNFRSF11B	266 (22)
HIF1A		transcription regulator		1.315	1.41E-06	ABCF2,AKAP12,BGN,CA9,CEMIP,CLDN1,CTGF,CXCL12,CYP4F3,EGLN3,ENO2,FHL1,FN1,FOS,FSCN1,GADD45B,GPER1,IGF1,IL15,INHBB,ITGA1,ITGB2,KDM4B,KIFC2,MMP2,NOV,NOX4,P4HA2,PDK1,PLOD2,PTGS2,SERPINE1,SLC16A4,STC2,TAF9B,TERT,TGFB3,TLR2,VEGFC	161 (9)
SMARCA4		transcription regulator		0.126	1.68E-06	ABCA1,ABCB1,AHR,ALDH2,ANO1,ARHGDIB,ASNS,BEND5,BIRC5,CD74,CDC6,CTGF,DLX2,EPHA4,ESPNL,F3,FAS,FN1,FOS,GADD45A,GCHFR,GJB5,GPR68,GSTP1,HEPH,HFE,IFITM3,IGF1,INHBA,ITGA3,ITGA7,KRT18,MAOB,MMP2,NAP1L3,NRP1,OCIAD2,PDE4B,PDK1,PER3,PLAT,PLEKHG2,PLS1,PTP4A1,RAMP1,RBL1,RBP1,RGS2,SEMA3B,SEMA7A,SLC11A1,SOX2,SPP1,STAMBPL1,SYK,TAGLN,TFB1M,TGFB2,TLR2,TMCC3,TMEM154,TRNP1,UBD,UBE2H,UNC13D,WDR45	
DUSP1		phosphatase		-0.47	1.82E-06	GDF15,MERTK,NRP1,PIK3R1,PLAT,PTGS2,PTPRK,THBD,TLR2,VEGFC,WISP2	
E2F3		transcription regulator	Inhibited	-2.573	2.73E-06	CA12,CCNA2,CCNB1,CDC6,E2F2,EDN1,FAM102A,FAM46A,HIST3H2A,HMGB2,HOXB9,IGF2,INHBA,MAD2L1,MYBL2,RBL1,RBPMS,RRM2,TERT,THBD,TIMP3,TK1,TMPO,TNFAIP2,UXT	
CTGF	-3.243	growth factor		0.915	3.77E-06	COL10A1,EGLN3,FN1,MMP13,MMP2,MMP3,SERPINE1,SOX2,TIMP1,TIMP3	98 (7)

CDK4		kinase			4.11E-06	CDC45,CDC6,CENPH,CENPK,CENPN,CPEB1,CPED1,DHFR,E2F7,EIF5A2,ELN,ENPP2,LRRTM2,MCM7,MFSD6,MMP3,MORC4,PBLD,PDCD4,PSMC3IP,RBMS3,RGS2,STARD4,TM7SF2,TP53INP1,TRIP13,ZNF367,ZNF423	
SMAD2		transcription regulator		1.179	4.55E-06	ACTA2,BCL2L11,FN1,FSTL3,MMP2,NET1,SERPINE1,SNAI1,SOX2,TIMP3,VEGFC	
PI3K (family)		group		-1.065	5.45E-06	ABCA1,ABCG1,ACTA2,BCL2L11,BIRC5,CXCL10,CXCL12,DDIT3,FOS,FOXO1,GLI1,ISG15,ITGA6,KLF2,MMP2,MMP3,PREX1,PTEN,PTGS2,SERPINE1,TGM2,TNFSF10,TXNIP	248 (18)
P38 MAPK		group		1.253	7.34E-06	ATF3,CTGF,CXCL10,CXCL12,CYP4F3,FAS,FN1,FOS,INHBA,ITGB3,ITGB4,KDELR3,MMP13,MMP2,MMP3,PLA2G4A,PLA2G7,PTGS2,RBP1,RND3,SCNN1A,SERPINE1,SNAI1,TERT,TGFA,TIMP1,TLR2,TNFSF10,TOP2A,VDR	315 (19)
IDO1		enzyme	Inhibited	-2.39	7.74E-06	SLC1A4,SLC1A5,SLC3A2,SLC6A9,SLC7A11,SLC7A5	
TP73		transcription regulator		-0.181	8.75E-06	ABCB1,ACTA2,ADA,ARNTL,BIRC5,CLMN,COL1A1,CTH,DBP,DDIT3,DHFR,DIMT1,EDN1,FAS,FGFR3,FKBP1B,GAB2,HIVEP1,IL4R,IL7,ITGB4,NAP1L3,PIEZO2,POLD2,PTEN,SERPINE1,SNAI1,STMN1,TCEAL1,TERT,VEGFC	198 (12)
RB1		transcription regulator			9.02E-06	BCL2L11,BIRC5,BRCA1,CCNA2,CCNB1,CDC6,CWC27,DHFR,FAS,FLT1,FOS,IGF1,LACTB,MAF,MYBL2,RAD51,RBL1,RRM2,TERT,TUBG1,UXT	91 (4)
AREG		growth factor	Inhibited	-3.352	9.53E-06	AURKB,BIRC5,C3,CCNA2,CCNB1,CDC45,FOXM1,HIST1H2AC,IFIT3,IQGAP3,MMP15,MYBL2,PCDHGA11,PLAU,PTGS2,RRM2,TOP2A	113 (7)
FOXO3		transcription regulator		-0.382	0.00001	AR,BCL2L11,BIRC5,BNIP3,CLDN1,CTGF,CXCL10,FOXO1,GADD45A,GADD45B,MCAM,MMP13,SERPINE1,SNAI1,TNFSF10,TXNIP	226 (13)
PDGF BB		complex		0.621	0.000012	ATF3,CTGF,DUSP5,EDN1,F3,FOS,FOSB,GADD45A,GEM,NR4A2,PLAU,RGS2,RHOB,RND3,SLC2A3,SLCO1A2,SYK,THPO,TOB1	
TP63		transcription regulator		-1.706	1.22E-05	ADA,ADAMTS1,BCL2L11,BRCA1,CCNA2,CDH2,CKS2,COL4A1,COL5A1,F3,FAS,FGFR3,FN1,FUBP1,GADD45A,INHBA,ITGA3,ITGB4,LYN,MAD2L1,MCM10,MMP13,NOTCH1,PLAU,POLD2,POSTN,PTEN,RAD51,RBBP8,RCC1,SERPINE1,SNAI1,TAGLN,TGFB2,TGFB3,TIPIN,TNFSF10,UBE2H,WNT4	242 (16)

PGR		ligand-dependent nuclear receptor		-0.546	0.000015	AK4,AMD1,CDC6,DDX21,EDN1,EZR,F3,FN1,FOS,GOT1,GPER1,IL1R1,ITGA6,ITGB4,KLF5,KLF9,KRT18,LAMB2,NET1,P2RY2,PLAU,PTGS2,PTP4A1,RASSF2,TGFB3,TM4SF1,VCAN,WNT4	198 (14)
FGF8		growth factor		-0.535	1.69E-05	AIF1L,CDK20,COL18A1,CRIP1,CYCS,DDAH2,FBXL16,FGFR2,LAMB3,RTKN2,RUNX3,SPP1,TM7SF2,VDR	
TCF4		transcription regulator		-0.721	1.91E-05	AXIN2,BIRC5,EDN1,FGF1,GLI1,IRS1,LAMC2,PLAU,SPP1,TERT,VCAN,WISP2	
IGF1	254.721	growth factor		0.53	2.04E-05	BCL2L11,BIRC5,DDIT3,ELN,F3,FN1,FOS,GAP43,IGF1,IGF2,IRS1,PLAU,SERPINE1,SLC20A1,SYN1,TUBB3	283 (21)
PAX8		transcription regulator		-1.159	2.16E-05	CCNA2,CDC6,DHFR,MCM3,TERT,TG	
CDKN1A		kinase	Activated	2.532	2.16E-05	BIRC5,BRCA1,CCNA2,CCNB1,CDC25B,CHEK1,EXO1,FOXM1,IL1R1,KRT18,MAD2L1,MMP3,STMN1,TGFA,TOP2A	227 (10)
FSH		complex		-1.859	2.25E-05	ACTA2,ACTG2,ADAMTS1,AKAP12,AMOTL2,ATP9A,BTG2,COL18A1,CYFIP2,DAPK1,DUSP9,EZR,FOXK2,GATA6,GEM,GOT1,GPRC5A,INHA,INHBB,ITGA3,KRT18,MAPK6,MMP2,MSMO1,NOL3,P4HA2,PGK1,PIK3CD,PRKD1,PTGER2,PTGS2,PTP4A1,RASSF2,RGS7,RHOB,SMAD6,TK1,TNFRSF11B,TOB1,TP53I11,UPP1,VEGFC,VGF	
CBX5		transcription regulator		-1.279	2.45E-05	ABCB1,ALDH3A1,AXIN2,BIRC5,C15orf48,CD68,CDC6,CEMIP,FAM198B,FKBP14,GCHFR,GCNT3,LAMB3,MAP1LC3A,OAS1,PRSS23,QPCT,SLC2A3,SLC7A7,SYTL2,TCEAL1,TM4SF1,TXNIP	
Jnk		group		0.26	2.64E-05	ACTA2,BCL2L11,BIRC5,BRCA1,CDH2,CTGF,CXCL12,EDN1,FOSB,GDF15,MMP13,MMP2,MMP3,PTEN,PTGS2,SERPINE1,TERT,TGM2,TIMP1,VCAN	281 (16)
STAT5A		transcription regulator			2.69E-05	ABCB1,ALDH3A1,AXIN2,C15orf48,CD68,CEMIP,CISH,EGLN3,FAM198B,FKBP14,GCHFR,GCNT3,LAMB3,MAF,MAP1LC3A,OAS1,PRSS23,QPCT,SLC2A3,SLC7A7,SYTL2,TCEAL1,TM4SF1,TXNIP	
EZR	5.903	other	Activated	2.02	2.89E-05	ATF3,ATF4,CDH2,DDIT3,PTGS2,TMEM8B,TRIB3	
KDM5B	4.625	transcription regulator		1.227	2.99E-05	BBS9,BRCA1,CCNB1,DDIT3,FBXO5,FHL1,GADD45A,INSIG1,ISG15,IVNS1ABP,LIMCH1,MCAM,MCM2,MCM3,NOL3,PBK,PDE3B,RECQL,REEP1,SCNN1A,TOP2A,TUBB2A	

Akt		group		0.028	4.41E-05	ABCB1,ACTA2,BIRC5,BMF,BTG2,CTGF,F3,FOS,FOXO1,IL1R1,MCAM,MMP2,MSI1,NET1,NOTCH1,PDK4,PTGS2,SERPINE1,SNAI1,TIPARP	231 (17)
NPPB		other	Activated	2.804	4.47E-05	ACAT2,EBP,FDFT1,HMGCR,HMGCS1,IDI1,LSS,MSMO1	
YAP1		transcription regulator		-1.316	4.72E-05	AMOTL2,AURKB,BCL2L11,BIRC5,CTGF,EDN1,FOXM1,MSLN,PTEN,PTGS2,SLC2A3,TAGLN	
MGEA5		enzyme		1.808	4.85E-05	ABLIM1,ARHGEF25,CD302,CLDN1,CPE,CYFIP2,EPHA4,EPHB3,FDFT1,FDPS,FN1,FSCN1,GADD45A,GPER1,GPT2,HMGCR,HMGCS1,IGF2,IL6R,ITGB7,ITPKB,LRIG1,LSS,MCAM,MED14,MSMO1,NSDHL,PDK1,PLAU,RHOB,SERPINB9,STARD4,TCF19,TGFA,TIMP1,TIMP3,UXT	
MXI1		transcription regulator	Activated	2.611	5.37E-05	CCNB1,FOXM1,IARS,LARS,MTHFD2,SLC1A4,SLC7A1	
SPDEF		transcription regulator	Inhibited	-2.673	5.42E-05	CDH11,CDH2,COL1A1,COL4A1,COL4A2,COL4A6,COL5A1,COL6A2,COL6A3,CTGF,ITGA3,ITGA6,LAMB2,PLAU,SERPINE1	91 (4)
SFN		other		1.342	7.44E-05	BTG2,CDH2,DSP,F3,IL1R1,KRT18,NET1,PDK4,PKP3	
CREB1		transcription regulator	Inhibited	-2.158	8.28E-05	BIRC5,CCNB1,CEBPD,FLT1,FN1,FOSB,MCM5,MMP13,MYBL2,NR4A2,PLA2G4A,PTGS2,RAD54L,RGS2,TSKU	
NUPR1	-3.647	transcription regulator		-0.041	8.37E-05	ACAD10,ACTR3B,AGRN,AKAP12,ANGEL1,AS3MT,ASIC1,ATF3,BNIP3,BRCA1,BRINP3,CCNA2,CD68,COL1A2,CYB5B,DDIT3,DEPTOR,DHTKD1,DUSP5,DUSP8,E2F8,ENO2,EXO1,FAM114A1,FAM162A,FGF1,GAB2,GADD45A,GBP2,GCH1,GDF15,GINS1,HFE,HSPA2,IL13RA1,IL6R,LRP8,MAN2B2,MCM10,NEIL3,NFIL3,P4HA2,PDK1,PER3,PFKFB4,PIK3R1,PTPRJ,PXDC1,RAD51,RNFT2,RTN4IP1,SERPINE1,SHCBP1,SHROOM3,SLC16A10,SPAG5,SPDL1,SPG7,SYTL2,THG1L,TMPO,TOB1,TRERF1,TRIB3,UNC5B,UPP1	
CCND1		transcription regulator		0.784	8.62E-05	AFAP1,AR,ARHGEF2,CDC45,CDC6,CENPH,CENPK,CENPN,CPEB1,CPED1,E2F7,EIF5A2,ENPP2,FAM120A,ITGB3,LAMB2,LRRTM2,MCM7,MFSD6,MMP3,MORC4,MYO7A,PBLD,PDCD4,PSMC3IP,RAD51,RBMS3,RGS2,STARD4,TALDO1,TM7SF2,TP53INP1,TRIP13,ZNF367,ZNF423	
FOXO1	2.994	transcription regulator		-0.352	8.75E-05	BCL2L11,BIRC5,BRIP1,CCNB1,CTGF,CXCL10,EDN1,FOXO1,GADD45A,GADD45B,IRS1,ITGA3,MCM5,MMP3,MTTP,SOX2,TNFSF10,TXNIP,WNT4	133 (4)

HOXD10		transcription regulator		-1.89	9.36E-05	EZR,ITGA3,ITGB4,NME1,RHOB,SERPINE1,TIMP1	
ENG		transmembrane receptor		-1	0.000103	BARD1,ITGA1,ITGA6,ITGB3,PLAU,SERPINE1	35 (3)
CLDN7		other		-0.444	0.000134	ABLIM1,ALDH3A1,ARHGDIB,C1S,C3,CA12,CD68,CTGF,CX3CL1,F3,LGR4,MLEC,MMP2,NETO2,NNMT,NTPCR,PHGDH,PKMYT1,PRSS23,SLC35B1,SLCO2A1,SYTL2,TEAD2	
F7		peptidase		-0.611	0.000153	AR,CTGF,F3,FOS,GADD45A,KLF5,MMP13,PTGER2,RND3	
SMAD1		transcription regulator		1.067	0.000175	ACTA2,BTG2,COL1A1,COL1A2,CTGF	
SP3		transcription regulator		-1.039	0.00019	ASNS,ATF3,BIRC5,CDH2,COL1A1,COL1A2,CYP51A1,FLT1,GDF15,HGF,IGF2,KRT16,MAOB,MMP2,PLAU,PREX1,PTGS2,RBL1,TERT	236 (12)
IL2		cytokine		-0.493	0.00021	ACVR1B,ADCY3,AHR,CCNG1,CDC6,CISH,CXCL12,DAPK1,EEF1E1,ENPP2,EPHA4,FAS,FGFR2,GADD45B,IDI1,IL18RAP,IL2RB,IL2RG,MAP2K6,NETO2,PDCD4,PDE3B,PDE4B,PDGFC,RFTN1,RHOB,SERPINB9,SESN3,SLC2A3,SPP1,TNFRSF11B,TNFSF10,TP53INP1,TRIB3	36 (2)
SIN3A		transcription regulator		-0.816	0.000212	COL1A1,COL1A2,DDIT3,GADD45B,GSTP1,PTGS2,SERPINE1,TERT,TXNIP	
GNE		kinase	Activated	2	0.000219	ASNS,CHAC1,DDIT3,TRIB3	
SEL1L		other			0.000219	NOTCH1,PTEN,TIMP1,TUBB3	
NFIL3	-3.81	transcription regulator			0.000219	FAS,GADD45A,GADD45B,TNFSF10	20 (2)
Lh		complex		-0.964	0.000222	ACTA2,ACTG2,AKAP12,AR,ATP9A,COL18A1,CYFIP2,DAPK1,DUSP9,EZR,GEM,INHA,INHBB,ITGA3,KRT18,MAPK6,MMP2,MSMO1,NOL3,P4HA2,PGK1,PIK3CD,PRKD1,PTGS2,PTP4A1,RAPGEF3,RGS7,RHOB,TK1,TNFRSF11B,TP53I11,UPP1,VEGFC	82 (3)
SYVN1		transporter		0	0.000223	ACSL3,ADAM9,AHR,AMOTL2,ASF1B,BCAT1,F3,GPRC5A,ITGA3,ITGA6,ITGB4,LDHB,MCAM,PTPRJ,SCARA3,SLC1A5,SLC20A1,SLC2A3,SLC3A2,SLC43A2,SLC7A5,TARS,TRERF1,UNC13D	
ERK		group		-0.811	0.000233	BCL2L11,BMF,COL1A1,CTGF,CXCL10,FAS,FOS,FOXO1,GDF15,ITGB2,ITGB3,MMP13,MMP2,ODC1,PDK4,PTGS2,SERPINE1,TGFA,TGM2,VCAN	218 (20)

CIP2A		other		0.64	0.000257	ACTG2,CRLF1,DCN,E2F2,ENO3,GADD45A,NPTX1,PDE2A,PDK4,PXYLP1,S100A16,SCIN,SLC22A18,SLC22A4	
MYOC		other			0.000261	ADAMTS1,CDH11,CHRDL1,DDIT3,FN1,FSCN1,NOL3,PTGER4,PXDN,RAB27B,RGS17,SLC2A3,SLC7A11,STC2,UPP1	
RBL2		other			0.00029	AURKB,BRCA1,CDC6,DHFR,MYBL2,RBL1,RRM2,TERT,UXT	
TGFBR2		kinase		0.915	0.00031	ACSL3,ACTA2,ATF4,CDHR1,COL4A6,GADD45B,GATM,MAN2B2,MMP2,MMP3,RBMS3,SERPINE1,SLC2A3,TIMP1,TXNIP	198 (8)
CTNNB1		transcription regulator		0.932	0.000325	ABCB1,ABCD2,AXIN2,BIRC5,CDH11,CDH2,DDIT3,EDN1,ENO2,EPHB3,FN1,IL1R1,INHBB,IRS1,ITGA1,LAMC2,LBH,MMP2,PDE4B,PLAU,QPCT,RAD23A,SEMA3C,SERPINE1,SLC1A5,TERT,VCAN	245 (11)
miR-21-5p (and other miRNAs w/seed AGCUUAU)		mature microrna		-1.119	0.000329	PDCD4,PIK3R1,PTEN,RECK,TIMP3,TNFRSF11B	
FBN1		other		-1.455	0.000329	COL1A2,COL4A2,COL6A3,CTGF,MMP3,TIMP3	
TAL1	28.393	transcription regulator	Inhibited	-2.286	0.000358	ADCY3,ARVCF,C3,CCNB1,CENPU,CIB2,CWC27,DNMBP,DSCC1,EEF1E1,GINS1,GSDMD,LRR1,LRRN3,MAP2,MCM2,MSLN,MVD,NEFL,NOS1,NOTCH1,NTNG2,OXR1,PTGER4,SLC2A3,SYK,TNFSF10,TOB1,UBE2H,YARS,ZNF827	
EPAS1		transcription regulator		-1.108	0.000364	ABCF2,AKAP12,CA9,CEMIP,CLDN1,CTGF,EDN1,EGLN3,ENO2,FHL1,FOS,INHBB,KDM4B,SERPINE1,SLC7A5,STC2,TAF9B,WISP2	92 (4)
FZD8		g-protein coupled receptor		0.465	0.000367	ACTA2,COL1A1,CTGF,FN1,VCAN	
HDL		complex		0.412	0.000367	ATF3,INSIG1,ITGB2,ITGB3,KLF2	
FAT1		other		-0.772	0.000367	MMP3,PDCD4,PLAU,PTGS2,VEGFC	
Pkc(s)		group	Activated	2.272	0.000372	ATF3,BCL2L11,DDIT3,F3,FOS,GADD45A,GADD45B,GPER1,HMGCR,IGF2,LIPA,MMP2,PTGS2,RGS2	266 (23)
STAT3		transcription regulator		1.792	0.0004	ACTA2,BIRC5,CA9,CDH2,CHEK1,COL1A1,CXCL10,DDIT3,EDN1,ESR2,FN1,FOS,FSCN1,MMP2,NFATC2,NOTCH1,NR4A2,PDK1,PGK1,PTGS2,SERPINE1,SNAI1,SOX2,STC2,TAGLN,TERT,TIMP1,TNS1,VCAN	271 (17)

MBD2		transcription regulator		-1.076	0.000469	BRCA1,CENPH,CKS2,EFNA5,GSTP1,MGMT,MMP2,NEIL3,NUPR1,ORC6,PLAU	
IL13		cytokine		-0.114	0.000482	ABCA1,ADA,ATF3,C3,CD14,CD48,CHST2,CISH,COL1A2,ENPP2,FAM162A,FGD2,IARS,IL13RA1,IL1R1,LIPA,MAF,MAOA,MCUR1,MRC1,MSMO1,PDGFC,PID1,RFTN1,RPS6KA2,SLA,SLC7A7,TGM2,TNS1	
TGFBR1		kinase		0.679	0.000545	BIRC5,CTGF,DOCK4,EDN1,PTPRK,SNAI1,VEGFC	27 (3)
EGR1		transcription regulator		-0.53	0.000583	ACTA2,ATF3,BCL2L11,COL1A2,F3,FN1,GDF15,GLI1,IGF2,IL6R,TNFSF10	
ERK1/2		group		0.699	0.000625	ABCA1,BCL2L11,BRCA1,C3,DDIT3,ESR2,EZR,FGFR2,FN1,FOS,FOSB,FOXO1,KCNH2,MAPK8IP1,MMP2,MMP3,PFKFB4,PTGS2,SNAI1,SPRY4,TERT,TIMP1,WISP1	297 (17)
TWIST1		transcription regulator		0.923	0.000643	AR,C3,CDH2,COL1A1,DCN,FAS,FGFR3,FMOD,FOXO1,MMP2	
Hdac		group		-0.531	0.000758	ARC,ATF3,BIRC5,DDIT3,FOS,GADD45B,KCNH2,KLF9,PIK3R5,PREX1,RGS10,SPP1,TXNIP	156 (6)
CCL2		cytokine		1.134	0.000779	EGLN3,IGF1,INHBA,MMP2,SERPINE1,SLC11A1,TIMP1	
TREM1		transmembrane receptor		-1.391	0.00078	ACSL3,ASNS,ATF3,CCL17,CENPU,CKS2,CSF1,DTNBP1,E2F7,EDN1,ELOVL7,F3,GADD45B,GEM,GPRC5A,GREM1,INHBA,ISG15,MOAP1,NR4A2,OSGIN1,PTGS2,RHOU,SPP1,STARD4,THBD,TMPO	
HOTAIR		other		0.744	0.000831	GDF15,LAMB3,LAMC2,OAS1,PCDH10,SNAI1	25 (2)
NR4A1		ligand-dependent nuclear receptor		-1.782	0.000831	ACTA2,BIRC5,COL1A1,COL1A2,SERPINE1,TNFSF10	
N-cor		group			0.000831	AXIN2,CXCL10,GSTP1,IGF1,INPP4B,PTGS2	
COL18A1	4.864	other	Inhibited	-2.197	0.000865	ANTXR1,F2RL1,F3,FN1,FOS,HGF,ITGB2,ITGB3,MMP2,NRP1,PLAU,PTGS2,SERPINE1,VWF	194 (13)
miR-34a-5p (and other miRNAs w/seed GGCAGUG)		mature microrna		1.557	0.000871	ATF3,BIRC5,DHFR,E2F2,MCM10,MCM3,NOTCH1,SOX2,TP53INP1	

HDAC2		transcription regulator			0.000871	BRCA1,COL1A2,DPT,FOXM1,IGF1,PREX1,PTGS2,RHOB,TERT	30 (3)
let-7		microrna		0.757	0.000909	ACTA2,AR,AURKB,BCAT1,BTG2,CDH2,CEBPD,E2F2,FN1,GAB2,HMGA1,ITGB3,LSM6,MAPK6,MCM2,PTGS2,SERPINE1	
E2f		group	Inhibited	-3	0.00109	CDC45,CDC6,DHPS,E2F2,GINS2,GMNN,MCM10,MCM2,MCM5,MYBL2,RAD51,RBL1,RECQL,RRM2,UXT	
BRD4		kinase		0.918	0.00116	ABLIM1,ACTA2,AURKB,COL1A1,COL1A2,COL4A1,COL5A1,FN1,FOS,ITGA1,KCNQ5,LOXL1,NME1,PDGFC,PLAT,PLAU,POLR3G,PTPN22,THBS2,TIMP1	
I kappa b kinase		complex	Activated	2.224	0.00117	BRCA1,BRCA2,FANCA,FANCC,RAD51	
STAT5a/b		group		1.066	0.00125	AHR,CISH,EPHA4,IDI1,MAF,NETO2,PDE4B,RBP1,RFTN1,SLC2A3	
MAP2K1		kinase		0.825	0.00125	ATF3,BCL2L11,DUSP5,F3,FOS,MMP2,NET1,PLA2G4A,RRAGD,UNC5B	154 (12)
NFE2L2		transcription regulator		-1.054	0.00125	ATF4,BRCA1,CTGF,DDIT3,OSGIN1,PHGDH,PSAT1,SERPINE1,SHMT2,TALDO1	
26s Proteasome		complex	Inhibited	-2.498	0.00133	ATG9B,BAG3,BCL2L11,BRCA2,CCNA2,CD68,CDC6,EXO1,HMGB2,MAD2L1,PTEN,RFC3,S1PR1,SNAI1,TGM2,TOP2A,UBE2H	185 (8)
TMPO	-2.455	other	Activated	2	0.00134	ASPN,COL12A1,COL1A1,MMP15	
MIA		other		1.982	0.00134	CDH2,FN1,ITGB3,PLAT	
Calcineurin protein(s)		complex		0	0.00134	BCL2L11,NFATC2,PTGS2,RGS2	
mir-21		microrna		-1.385	0.00148	ACTA2,FN1,PDCD4,PTEN,RECK,TIAM1,TIMP3	
EIF4G1		translation regulator		-1.89	0.00148	ATRX,BIRC5,BRCA1,BRCA2,CHEK1,GADD45A,RAD51	
SBDS		other			0.00149	ADD2,CHAC1,COL27A1,CRABP1,CTGF,CTH,DDIT3,EPDR1,FBXL16,FOS,GADD45B,GAP43,IL4R,MAOA,SPP1,TRIB3	
NRG1		growth factor		0.022	0.0015	ABCA1,BRCA1,FN1,FOS,HMGA1,ITGB3,SNAI1,VEGFC	248 (15)
RBL1	-3.257	transcription regulator			0.0015	AURKB,CDC6,DHFR,MYBL2,RBL1,RRM2,TERT,UXT	
VEGFA		growth factor	Activated	2.244	0.00153	CXCL10,F3,FLT1,ITGA1,ITGB3,MMP13,MMP2,NOTCH1,NRP1,PLAU	247 (20)

Raf		group			0.00153	ETV5,GDF15,PTEN,RND3,SEMA3C,SEMA6A,SLC20A1,SPRY1,SPRY4,STON1	40 (3)
ZNF217		transcription regulator		-1.134	0.00158	ATL1,GPRC5A,KRT18,NEFL,NLGN1,NMNAT2,PLAT,SH3RF2,SHC4,STRA6,TDGF1,WNT5B,ZHX2,ZNF616	
EP300		transcription regulator		-1.471	0.00166	AR,AXIN2,BIRC5,CA12,GREB1,IGF1,ITGB2,MGMT,PLA2G4A,PLA2G7,PTGS2,SMAD6,SOX2,TERT,TLR2	245 (15)
CD24		other	Inhibited	-2.28	0.00169	ADD3,ATF3,CHAC1,DEPDC1B,DUSP5,GDF15,KNTC1,LDB1,MCAM,PHF10,PLAU,PLPPR2,PTP4A1,SLC4A4,TOP2A,TP53INP1,TUFT1,VDR	
BCL2L1		other		0.333	0.00175	COL6A2,FAS,FN1,ITGA3,MAP2,TIMP1,TNFRSF11B,TNFSF10,TUBB3	74 (4)
SIN3B		transcription regulator			0.00178	COL1A2,DDIT3,DHFR,GADD45B,RRM2,TXNIP	
TCR		complex		1.696	0.00183	ABLIM1,BCL2L11,CISH,CS,CXCL10,FOS,GBP4,HSPA9,IFIT3,IL7,ISG15,ITGB7,LDHB,LIPA,MAF,NME1,NRP1,OAS1,ODC1,PDCD4,PDE3B,PGK1,PIK3CD,PIK3R1,SIAH2,SLC1A5,SLC2A3,TALDO1,TLE3,UNC13D,VDAC3,VDR	131 (10)
PTTG1		transcription regulator			0.00187	CCNA2,CCNB1,CDH2,SNAI1,SOX2	177 (7)
SYK/ZAP		group			0.00213	PTGS2,TIMP1,VEGFC	231 (13)
miR-515-5p (and other miRNAs w/seed UCUCCAA)		mature microrna			0.00213	FGFR2,PIK3C2B,TCF7L1	
miR-19b-3p (and other miRNAs w/seed GUGCAAA)		mature microrna			0.00213	BCL2L11,BIRC5,PTEN	
RGCC		other			0.00213	ACTA2,CDH2,SNAI1	
NEDD9		other		1.667	0.00216	BNIP3,CA9,FOS,GDF15,MMP2,PGK1,PLOD2,SERPINE1,TXNIP	76 (4)

ESR2	4.894	ligand-dependent nuclear receptor		1.751	0.00224	BIRC5,C3,CCNA2,CXCL12,FOS,FOXM1,GSTP1,TERT,TGFA,VAV3	99 (7)
TGFB2	11.266	growth factor		0.563	0.00248	DOCK4,FN1,FOS,PLOD2,SCD,VCAN	
RNF20		enzyme		0	0.0025	FOS,HBA1/HBA2,NR4A2,RHOB	
mir-25		microrna		-1.98	0.0025	BCL2L11,ESR2,ITGB3,PTEN	
RCOR1		transcription regulator			0.0025	COL1A1,COL1A2,PTGS2,SERPINE1	
EFNA2		kinase		-1.508	0.00262	BACH2,CTGF,ETV5,FOXL1,ITGB4,KRT16,KRT18,NFIL3,PKP1,PLAT,SLC20A1	
RUNX2		transcription regulator		1.213	0.00269	ACTA2,CDH2,COL10A1,FGFR2,MMP13,MMP2,PLAU,SERPINE1,SNAI1,TAGLN	70 (7)
NFkB (complex)		complex		0.01	0.00273	ABCB1,AHR,C3,CCL17,CCNB1,CDC25B,CX3CL1,CXCL10,CXCL12,EDN1,ERAP1,F3,FAS,FOXF1,GDF15,HLA-DMB,IL15,LSP1,MMP13,MMP2,MMP3,NOS1,NOTCH1,PLAU,PTGS2,RFTN1,SLC7A5,SPIB,TACR1,TGM2,TNFSF10,TRIB3,VEGFC	260 (11)
FOXM1	-2.818	transcription regulator	Inhibited	-2.191	0.00274	AURKB,AXIN2,BIRC5,CCNA2,CCNB1,CDC25B,CKS2,FOXF1,FOXM1,MMP2,PGK1,PLK4,SOX2,STMN1	193 (10)
SAA		group		1.485	0.00283	CCL17,F3,IL33,MMP13,MRC1	142 (8)
SIM2		transcription regulator		-0.109	0.00283	BNIP3,CDH2,GADD45A,MMP2,MMP3	
PDCD4	2.78	other		-0.277	0.00283	MMP3,PLAU,PTGS2,SNAI1,VEGFC	
S100A4		other			0.00283	BNIP3,MMP13,MMP2,PTEN,TIMP1	111 (9)
FGF2		growth factor		1.709	0.00322	BGN,BIRC5,DCN,F3,FLT1,MGMT,PLAU,SERPINE1,THPO	192 (16)
GLI1	-3.917	transcription regulator		1.622	0.00323	ARC,C11orf96,CCSAP,COL10A1,COL1A1,FAS,FGF1,FHL1,FUCA2,MMP2,NAV3,NREP,RGS10,RPS6KA1,SESN3,SPP1,VEGFC,ZC3HAV1L	
HIC1	7.81	transcription regulator		0.093	0.00333	ABHD3,ACTA2,AMIGO2,CA12,INHBA,KAZN,LRP8,MCAM,NOV,PLA2G4A,SLC7A11,SPP1	
Histone h4		group			0.00333	ABCB1,ADA,DHFR,FOXM1,MGMT,POLD2,PREX1,PTGS2,RBL1,RRM2,TERT,TNFSF10	152 (6)
HDAC6		transcription regulator		1.698	0.00335	ADAMTS1,BIRC5,COL1A1,IGF1,LTBP2,PLAU,SERPINE1	

Hsp27		group		-0.57	0.00335	CD14,CSF1,EIF4EBP1,FOS,IL6R,NOX4,PTGS2	97 (7)
NR3C2		ligand-dependent nuclear receptor		1.741	0.00338	CNKSR3,INHBA,PLAT,PTGS2,SCNN1A,SERPINE1	
DCAF1		kinase		-0.883	0.00338	BMF,NOV,TGM2,TNFSF10,TOB1,TXNIP	
SIRT1		transcription regulator		1.578	0.0038	BIRC5,BNIP3,CDH2,FGF21,FN1,HMGCR,IGF1,MMP2,MMP3,TERT	179 (8)
PTP4A3		phosphatase		-0.246	0.00411	FN1,ITGB3,PTEN,SNAI1,STMN1	96 (7)
HGF	-198.447	growth factor		0.867	0.00414	CA9,DDX21,FOS,HLX,IGF1,ISG15,MERTK,MMP2,ORC2,PGK1,PLAU,PTGS2,RHOB,TAGLN2,TMEM97,TNFAIP2,VEGFC	278 (23)
IL17A		cytokine		0.017	0.00419	ACTA2,CD14,CD68,COL1A1,CTGF,GADD45A,MMP3,MRC1,NRP1,PTGS2,TIMP1	300 (22)
RLIM		enzyme		1	0.0042	CDH2,CTGF,FN1,SERPINE1	13 (2)
SPARC		other		0.851	0.0042	MAP2,NOTCH1,SERPINE1,SNAI1	129 (12)
mir-19		microrna		-1	0.0042	ABCA1,BCL2L11,MSMO1,PTEN	
miR-210-3p (miRNAs w/seed UGUGCGU)		mature microrna		-1.964	0.0042	EFNA3,FGFRL1,NPTX1,TP53I11	
TPM3		other	Inhibited	-2	0.0042	IGF1,ITGA1,ITGA3,MMP2	
MNT		transcription regulator			0.0042	DDIT3,GADD45B,TERT,TXNIP	
ELK1		transcription regulator		-0.64	0.00426	MMP13,MTHFD2,NUPR1,SLC7A11,SNAI1,SPP1,TIPARP	
mir-29		microrna		-0.744	0.00426	ADAM12,AOX1,CLDN1,IGF1,ITGA11,NAV3,PTEN	
BCL6		transcription regulator		-0.57	0.00441	ALCAM,BLNK,CHEK1,CISH,COL1A1,FCGRT,FMOD,GADD45A,ITGA3,SERPINE1,SOX5,SYK	
EZH2		transcription regulator		-0.898	0.00444	AXIN2,C15orf48,C3,CEMIP,CX3CL1,CXCL10,DAB2IP,DENND2A,FLT1,FZD1,GDF15,GPR68,LAMB3,LAMC2,MMP2,PCDH10,PIK3IP1,PKMYT1,PLA2G4A,PTGS2,RAP1GAP,SNAI1,TGFB2,UBD	
NCOR2		transcription regulator			0.00449	AR,AXIN2,C3,FOS,IGF1,PTGS2	104 (5)

MYB		transcription regulator		1.134	0.00462	AXIN2,BIRC5,CCNB1,CDH2,CXCL12,FN1,MMP3,SNAI1	
RNA polymerase II		complex			0.00466	BLNK,BRCA1,CA12,CCNB1,CDH11,CSDC2,FOS,GADD45A,GDF15,GOT1,MCM3,MMP2,MYBL2,PKMYT1,PTGS2,RAD51AP1,RBBP8,RND3,TERT,TOP2A	184 (7)
STAT1		transcription regulator		0.213	0.00481	ABCB9,C1S,C4A/C4B,CD14,CX3CL1,CXCL10,FAS,GBP2,GBP3,GBP4,IFIT3,ISG15,LRRTM2,NOX4,OAS1,SLAMF8,TNFSF10,WARS	
SYK	-4.465	kinase	Inhibited	-2.598	0.00485	AKAP12,BCL2L11,CXCL10,DOCK10,GADD45A,KLF2,OAS1,TNFSF10,TP53INP1,TRIB2,YPEL2	26 (2)
KDM3A		transcription regulator			0.00499	EDN1,GDF15,SERPINE1	
GATA6	10.44	transcription regulator		-0.355	0.00524	ABCB1,CEBPG,COL4A1,COL4A2,DSCC1,EDN1,GATA6,SOX2,TGFB2,TNFSF10	
MDM2		transcription regulator		-0.335	0.00553	CLIP3,ESR2,FCGRT,INHBB,LYZ,NDUFA1,TERT,TIMP1,TNFSF10	
CXCL8		cytokine		-0.535	0.00553	AR,CD74,COL12A1,COL18A1,IFRD1,ITGB2,MMP2,PTGS2,SOX2	
TRAP1		enzyme	Activated	2.121	0.00562	AK3,ALDH1L2,GARS,GPT2,MARS,MTHFD2,PTGS2,SARS	
SMARCD3		transcription regulator		0.447	0.00574	ALCAM,COL1A1,FMOD,ITGA3,SOX5	
TBXT		transcription regulator		0.447	0.00574	ALCAM,COL1A1,FMOD,ITGA3,SOX5	
PTAFR		g-protein coupled receptor		0.381	0.00574	BIRC5,CDH2,MMP2,PTGS2,SNAI1	236 (17)
mir-17		microrna		-0.527	0.00574	AR,BCL2L11,CAPRIN2,CTGF,FAS	
NKX2-1		transcription regulator		-1.974	0.00574	MMP2,SERPINE1,SNAI1,TG,TGFB2	
SUZ12		enzyme		-0.447	0.00615	ANXA8/ANXA8L1,DAB2IP,INSIG1,IVNS1ABP,LAMB3,LAMC2,MMP2,PCDH10,PLAU,PTEN,RAN,SLC7A5,SNAI1,TNFAIP2,TNFRSF11B	
RELA		transcription regulator		0.239	0.00649	ARHGDIB,BACH2,BTG2,COL1A1,CXCL10,EDN1,ERAP1,FAS,FCGRT,FN1,FOSB,FSCN1,GCH1,LYN,MGMT,NR4A2,PDE4B,PLAU,PTGS2,SLC2A4,TGM2,TLR2,TUBB3,UBD	208 (15)

CEBPA		transcription regulator		-0.607	0.0065	AKAP12,ANPEP,ASNS,BTG2,C3,FOXO1,GCH1,ISG15,KLF5,NFATC2,NFIL3,PLOD2,PTGS2,RGS2,SPP1,THBD,TNFSF10,TUBB2A,VDR	
BRCA1	-2.959	transcription regulator		-0.891	0.00654	BIRC5,BRCA1,CCNB1,DDIT3,EXO1,FOXO1,GADD45A,GDF15,PTEN,RAD51,TERT,VCAN	294 (20)
DEF6		other		1.996	0.00655	CDH2,FN1,MMP2,SNAI1	
let-7a-5p (and other miRNAs w/seed GAGGUAG)		mature microrna		-1.274	0.00655	ACTA2,BIRC5,CDH2,COL1A2	
MUC1		other		0.278	0.00676	CDH11,CHEK1,EGLN3,MRC1,PLAU,POLD2,PYCR1,TERT	86 (4)
CD3 group		group		-1.147	0.00676	ACSL3,ACSL4,CBLB,HAVCR2,PLA2G4A,PLA2G4B,SCD,SLC7A5	
EGFR		kinase		-0.491	0.00678	ACTA2,AR,BCL2L11,CTGF,DDIT3,E2F2,F3,FOS,HERPUD1,IGF1,ITGA6,MYBL2,PLAU,POSTN,PTGS2,SLC7A11,TGM2	271 (20)
MCPH1		other			0.00685	BRCA1,CHEK1	
IPMK		kinase			0.00685	FOS,RAD51	
DNAJC3		other			0.00685	ATF4,DDIT3	
MXD1		transcription regulator			0.00685	ODC1,TERT	
mir-196		microrna			0.00685	COL1A1,FOS	
miR-292-3p (and other miRNAs w/seed AGUGCCG)		mature microrna			0.00685	AR,BIRC5	
mir-95		microrna			0.00685	AR,BRCA1	
NCK1		kinase			0.00685	ATF4,DDIT3	
BCAN		other			0.00685	CDH2,ITGB3	
RBP3		transporter			0.00685	ATF4,DDIT3	
BECN1		other			0.00685	BCL2L11,BMF	

BARD1	-2.537	transcription regulator			0.00685	AURKB,BRCA1	
RBFOX2		transcription regulator			0.00685	PLOD2,PTEN	
MAPK1		kinase		-0.998	0.00686	ADAM12,AR,BIRC5,C1S,CCNB1,DEPTOR,ERAP1,FN1,FOS,HBA1/HBA2,IFIT3,IFITM3,ISG15,ITPR2,MYBL2,OAS1,PHF11,PLA2G4A,PTGS2,RAD54L,SPRY4,TNFSF10,TRIM14,TSKU,VDR	120 (7)
CDKN2A		transcription regulator	Activated	2.07	0.00735	BIRC5,CCNA2,CCNB1,CDC25B,FOS,MMP3,MRC1,PTEN,PTGS2,SERPINE1,SOX2	323 (22)
PPARG		ligand-dependent nuclear receptor		-0.353	0.0074	ABCG1,ACTA2,BIRC5,COL1A1,COL1A2,FN1,INSIG1,PDK4,PTEN,PTGS2,SERPINE1,TNFSF10	75 (4)
TAB1		enzyme		0	0.00749	CXCL10,GBP2,GCH1,MMP13,RGS2,TNFSF10	70 (5)
JAK		group		-0.277	0.00749	CDH2,CXCL10,IFIT3,IFITM3,ISG15,PTGS2	144 (12)
SATB1		transcription regulator		0.286	0.00757	ADCY3,EPSTI1,FAM129A,GADD45B,GPT2,HLA-DMB,HVCN1,IL18RAP,LRRN3,NR4A2,PIK3IP1,PTGS2,S1PR1,TMEM2,WARS	
MAP3K7		kinase		0.333	0.00767	CXCL10,GBP2,GCH1,IFIT3,MMP13,MMP3,RGS2,SERPINE1,TNFSF10	239 (14)
FGF7		growth factor	Inhibited	-2.169	0.00779	CEBPD,FGFR2,HPRT1,IL7,SCD	
PIK3CA		kinase			0.00779	BIRC5,CCNB1,CD14,FOXM1,PIK3CD	129 (6)
CUX1		transcription regulator			0.00808	ARHGDIB,CDH2,CXCL10,ITGB2,PCDH10,SNAI1,WISP2,WNT10A	
UPF2		other		0.436	0.00812	CACNB2,CYFIP2,DSP,HEY1,NRP1,PTGS2,TNFRSF11B	
ATF6		transcription regulator		0	0.00812	DAPK1,FDPS,FOS,HERPUD1,RAN,SLC35B1,UNC13B	
MAPK14		kinase		0	0.00812	AXIN2,FOS,GREM1,MMP13,PTEN,PTGS2,VDR	152 (12)
YBX1		transcription regulator		-0.946	0.00812	ABCB1,ACTA2,COL1A2,FOS,MMP3,PTGS2,STC2	
TFAP2A		transcription regulator		1.225	0.00817	ABCA1,ALCAM,BIRC5,GEM,MMP2,PLAU,RAB27B,SESN3,TERT,WISP2	
AURK		group	Inhibited	-2.53	0.00817	BTG2,CYFIP2,CYP26B1,FAM212B,HSPA4L,PDE2A,PHLDB3,SRGAP3,SULF2,TCEA3	

ANLN		other	Inhibited	-2.53	0.00817	BTG2,CYFIP2,CYP26B1,FAM212B,HSPA4L,PDE2A,PHLDB3,SRGAP3,SULF2,TCEA3	
NOTCH1	4.414	transcription regulator		-1.234	0.00837	ACTA2,ENO2,HEY1,IL18,MMP3,PTPRK,RHOU,RND3,RUNX3,TGFB2,TGFB3	30 (2)
IL6		cytokine		0.469	0.00839	ABCA1,AFP,BIRC5,C3,CEBPD,CXCL10,DCN,E2F2,ENO2,FOS,IL6R,LYZ,MERTK,MMP13,MMP2,PLAU,PTGS2,TGM2,TIMP1	244 (21)
TGFBR		group			0.00937	ACTA2,CDH11,NOX4	
L1CAM		other			0.00937	IDI1,MMP2,SQLE	
mir-296		microrna			0.00937	CA9,CALML4,COL1A1	
mir-145		microrna			0.00937	CCNA2,FSCN1,TAGLN	
TNFRSF 11A		transmembrane receptor			0.00937	FN1,SNAI1,SOX2	
MAP2K7		kinase			0.00937	CXCL10,FN1,MMP3	
S1PR1	-3.869	g-protein coupled receptor			0.00937	HGF,IGF1,PTGS2	
S1PR3		g-protein coupled receptor			0.00937	HGF,IGF1,PTGS2	
NAB2		transcription regulator			0.00937	F3,FLT1,PLAU	
SF1		transcription regulator			0.00937	ACTA2,COL1A1,FN1	
NET1	-3.109	other			0.00937	CDH2,FN1,SERPINE1	
WNT3A		cytokine			0.00937	AHR,AXIN2,TMEM2	
miR-122-5p (miRNAs w/seed GGAGUGU)		mature microrna		-0.102	0.00939	BACH2,CCNG1,CS,EGLN3,ENTPD4,FUBP1,NCAM1,OSMR,SLC7A1,SLC7A11	
ATF3	-18.175	transcription regulator		-0.218	0.00944	ASNS,ATF3,AURKB,CHAC1,SERPINE1,SLC11A1	
MAX		transcription regulator			0.00957	DDIT3,DHFR,GADD45A,GADD45B,LRRN3,RBBP8,TERT,TXNIP	

SERPINF 1		other		1	0.00963	AXIN2,CTGF,ENO2,TNFSF10	84 (4)
EIF2AK3		kinase		-0.991	0.00963	ANG,ATF4,CA9,DDIT3	
VTN		other			0.00963	F3,IRS1,ITGB3,SERPINE1	69 (7)
ITGB1		transmembr ane receptor		-0.561	0.00985	COL1A1,EIF4EBP1,FOS,ITGB3,PLAU,PTGS2,SNAI1	297 (22)
NSD2		enzyme			0.00985	BACE2,BTG2,E2F2,GADD45A,IGF1,ITGB7,TUBB4A	
mir-223		microrna		1.432	0.0103	ABCA1,HMGCS1,MSMO1,NFIA,STMN1	
LGALS1		other		1.253	0.0103	ACTA2,FN1,GLI1,MMP13,MMP3	
ACSL5		enzyme		0.447	0.0103	ADAMTS6,GPRC5A,MAP2,MCAM,PPP4R4	
LMNA		other		0.152	0.0103	COL12A1,MMP3,PTEN,TIMP3,TMPO	
EGLN		group		-0.088	0.0112	BNIP3,CA9,CSRP2,DYRK4,EFNA3,EGLN3,EIF4EBP1,GPR146,KDM5B,NOL3,PDK1,PEX11A,RNF165,RRAGD,SAP30	
MYC		transcriptio n regulator		0.601	0.0112	ADAMTS1,ALCAM,BCAT1,CCNB1,CHEK1,COL1A1,CYFIP2,DDIT3,E2F2,EZR,FMOD,FOXM1,GADD45A,GADD45B,ITGA1,ITGA3,ODC1,PDCD4,PDK1,PLAU,PYCR1,RAD51,RCC1,SHMT1,SHMT2,SLC22A4,SOX5,SPP1,TERT,TXNIP,UXT	
POU5F1		transcriptio n regulator		1.658	0.0116	AFP,BAG3,BCL2L11,BFAR,BNIP3,CDH2,DAPK1,DLX4,FAS,GADD45A,GATA6,HOPX,KRT18,MMP2,NCAM1,SNAI1,SOX2,TNFRSF11B,TNFSF10	
KMT2D		transcriptio n regulator		1.546	0.0117	CRIP1,ENO3,FHL1,LAMB3,LOXL1,SMAD6	
BMP7		growth factor		0.223	0.0117	ACTA2,BIRC5,IGF1,IGF2,MMP13,MMP2	
SREBF1		transcriptio n regulator	Inhibited	-3.04	0.0126	AARS,AK4,CD14,CYCS,CYP51A1,ELOVL7,FDPS,FOXO1,HFE,NUPR1,P4HA2,PCK2,RBP1,SCD,SLC20A1,SLC22A4,VGF	
WISP2	28.193	growth factor		-0.788	0.0132	CLDN1,CTGF,DSP,FN1,KRT18,LAMB3,LAMC2,SERPINE1	
CREBBP		transcriptio n regulator			0.0132	BIRC5,CXCL10,FOSB,NR4A2,PTGS2,RGS2,TLR2,TNFSF10	
TWIST2	-2.803	transcriptio n regulator		1.274	0.0133	CDH2,FN1,POSTN,SNAI1,SOX2	
IL1		group		0.468	0.0133	FOS,MMP13,MMP3,PTGS2,TNFRSF11B	
SCD	-2.646	enzyme		1.961	0.0135	ATF3,DDIT3,GADD45A,HERPUD1	
mir-30		microrna		-0.781	0.0135	ADAM12,AR,BCL2L11,GADD45A	

PTGS2	-9.846	enzyme		0.095	0.0139	CXCL10,EZR,FLT1,ITGA6,ITGB4,MMP2,PPA1,PTGS2,SEMA7A,TNFSF10	
UXT	-4.354	transcription regulator		0.768	0.0141	BRIP1,CCNA2,CDC6,CHEK1,ENO2,KDELR3,KRT18	
PRKCE		kinase		-1.302	0.0141	ABCB1,AMIGO2,BIRC5,IL2RB,PDCD4,PTGS2,RGS2	
NCOA3		transcription regulator		-1.987	0.0141	CCDC80,CCNA2,CDC6,IGF1,MCM7,OTUB2,PTGS2	
PDLIM2		other	Inhibited	-2.138	0.0144	CEMIP,ENPP2,F2RL1,FHDC1,IFIT3,IL7,NAP1L2,NR2F1,RASGEF1A,SCARA3,STC2,TAGLN,TNS1,TXNIP	
MET		kinase	Activated	2.267	0.0153	CDH2,FN1,HLX,ITGA3,MMP2,SNAI1,SOX2,TGFA	
EFNA5	4.977	kinase		-0.707	0.0153	CTGF,ETV5,ITGB4,KRT16,KRT18,PKP1,PLAT,SLC20A1	
TLR7/8		group		-1.463	0.0153	EDN1,GEM,PLLP,PTGS2,RECQL4,SLC7A11,TRIB3,VDR	
ZNF100		other			0.0154	POSTN,SI,TGFB3	
ZNF85		transcription regulator			0.0154	POSTN,SI,TGFB3	
ZNF254		other			0.0154	POSTN,SI,TGFB3	
RASSF8		other			0.0154	POSTN,SI,TGFB3	
ZNF431		other			0.0154	POSTN,SI,TGFB3	
E2F7	-2.516	transcription regulator			0.0154	CCNA2,CCNB1,CDC6	
IGF2R		transmembrane receptor			0.0154	POSTN,SI,TGFB3	
miR-221-3p (and other miRNAs w/seed GCUACAU)		mature microrna			0.0154	PIK3R1,PTEN,TIMP3	
mir-214		microrna			0.0154	ALCAM,BIRC5,PTGS2	
EED		transcription regulator			0.0154	DAB2IP,MMP2,PTGS2	
mir-154		microrna			0.0154	BCL2L11,BIRC5,PTEN	
ZNF665		other			0.0154	POSTN,SI,TGFB3	
ZNF528		other			0.0154	POSTN,SI,TGFB3	
RACK1		enzyme			0.0154	FOXM1,GLI1,ISG15	

ERBB3		kinase			0.0154	FN1,PTGS2,SNAI1		
ZNF43		other			0.0154	POSTN,SI,TGFB3		
ZNF429		other			0.0154	POSTN,SI,TGFB3		
TAZ		enzyme			0.0154	CTGF,EDN1,TAGLN		
RPS6KA3		kinase			0.0154	DDIT3,FOS,FSCN1		
ZNF91		transcription regulator			0.0154	POSTN,SI,TGFB3		
ZNF708		other			0.0154	POSTN,SI,TGFB3		
PRKN		enzyme			0.0154	ACTA2,MAOA,MAOB		
ATM		kinase		-0.482	0.0166	CTGF,FN1,GADD45A,GADD45B,RRM2,SERPINE1,TGM2		
Notch		group		-0.647	0.0166	BIRC5,GADD45B,HEY1,MMP2,NET1,SMAD6,TAGLN		
WNT5A		cytokine		1.308	0.0168	AXIN2,COL1A1,FN1,IL15,SNAI1		
KLF5	-3.133	transcription regulator		-0.169	0.0168	BIRC5,CDT1,NOTCH1,SOX2,WNT10A		
APP		other		-0.655	0.0168	ABCA1,PTGS2,TNFAIP2,TNFSF10,TUBB3		
STK11		kinase		-1.207	0.0168	CCNA2,ETV4,FN1,SERPINE1,SNAI1		
miR-486-5p (and other miRNAs w/seed CCUGUAC)		mature microrna		-1.516	0.0168	AFF3,FOXO1,PTEN,SLC4A8,TOB1		
REST		transcription regulator		0.919	0.0174	GAP43,MAD2L1,MAPK8IP1,NRP1,SYN1,TUBB3		
SND1		enzyme			0.0174	ALDH3A1,CDC6,DHFR,NR2F1,PTGS2,QPCT		
GH1		growth factor		-1.067	0.0183	ATF3,PTEN,SNAI1,TWIST2		
WWTR1		transcription regulator			0.0183	CTGF,FN1,ITGB2,SERPINE1		
PIK3R1	-2.601	kinase			0.0183	AR,PIK3CD,PIK3R1,PLAU		
EHMT2		transcription regulator			0.0183	BIRC5,CXCL12,GREB1,PTGS2		

NR3C1		ligand-dependent nuclear receptor		-0.415	0.0188	AMIGO2,BAG3,BARD1,BCL2L11,BMF,BRCA1,ELMOD3,ENDOG,FN1,FOXO1,GADD45A,GADD45B,GEM,IL15,IL18,INHBA,ITGB2,MAOA,MAP4K3,MOAP1,NAV3,NOL3,NRG2,PDCD4,PDE4B,PIK3CD,PIK3R1,PLA2G4A,PTGS2,RBMS3,RGS2,RHOB,SCNN1A,SERPINB9,SERPINE1,SH3KBP1,SIAH2,SPP1,THBD,TIMP3,TNFAIP2,TNFRSF11B,TRIB3,UNC13B,WDR31	
ENTPD5		enzyme			0.0194	COL1A1,COL1A2	
GALNT3		enzyme			0.0194	CDH2,FN1	
PTPN23		phosphatase			0.0194	CDH2,SNAI1	
AKR1C3		enzyme			0.0194	PROS1,SIAH2	
TRIM65		other			0.0194	PDCD4,PTEN	
Inflammasome (Nalp3, Asc, Casp1)		complex			0.0194	FAS,SCD	
APOA4		transporter			0.0194	FOXO1,MTTP	
MBNL1		other			0.0194	ABLIM1,PTEN	
CBX1		transcription regulator			0.0194	BIRC5,PROS1	
NEUROD1		transcription regulator			0.0194	NCAM1,SLIT2	
miR-92a-3p (and other miRNAs w/seed AUUGCAC)		mature microrna			0.0194	BCL2L11,PTEN	
AMBP		transporter			0.0194	ACTA2,PLAU	
NOS3		enzyme			0.0194	CXCL12,GSTP1	
DDR1	3.231	kinase			0.0194	CDH2,COL1A1	
ITGA3	4.591	other			0.0194	PLAU,PTGS2	
ARID1A		transcription regulator			0.0194	PIK3IP1,TERT	
LIF		cytokine			0.0194	ERAP1,HGF	

MMP1		peptidase			0.0194	CXCL12,TACR1	
Muscarinic cholinergic receptor		group			0.0194	FOS,RGS2	
PTEN	3.004	phosphatase		1.391	0.0195	AR,BCL2L11,BIRC5,CCNB1,MCAM,PREX1,PTEN	
H2AFY	-2.51	other		-0.378	0.0195	BRCA2,FN1,GADD45A,MAP2K6,PLAU,SERPINE1,TERT	
HMOX1		enzyme		-0.113	0.0203	CXCL10,FGF1,HGF,IGF1,ITGB3,MAPT,TGFB2,THBS2	
EFNA4	2.789	kinase		-0.707	0.0203	CTGF,ETV5,ITGB4,KRT16,KRT18,PKP1,PLAT,SLC20A1	
EFNA3	6.452	kinase		-0.707	0.0203	CTGF,ETV5,ITGB4,KRT16,KRT18,PKP1,PLAT,SLC20A1	
IFNA2		cytokine		0.736	0.0203	BIRC5,CISH,CXCL10,FAS,GBP2,GOT1,HMGCS1,IFIT3,IFITM3,ISG15,OAS1,TNFSF10,TRIM14	
HMGA1	-6.747	transcription regulator	Activated	2.236	0.0208	HMGCR,IDI1,INSIG1,MVD,MVK,PTGS2	
PRNP		other		-1.732	0.0208	ABCB1,AHR,IGF2,IRS1,MMP2,TCF7L1	
FOSL1		transcription regulator		1.091	0.021	MMP13,MMP2,MMP3,SERPINE1,SNAI1	
AURKB	-2.445	kinase		-1.342	0.021	ATF3,CCNA2,CYP4F3,MCM3,RFC3	
mir-486		microrna		-1.446	0.021	AFF3,FOXO1,PTEN,SLC4A8,TOB1	
Gm-csf		group		-1.782	0.0227	BIRC5,CD14,MED14,MRC1,PTGS2,TERT,TRIB2	
ETV5	-3.532	transcription regulator			0.0227	ALCAM,CDH2,CLDN1,FN1,KRT16,MMP2,PKP3	
SAFB		other		0.426	0.0229	CD74,CX3CL1,CXCL10,FOS,NOV,NUPR1,TNFRSF11B,TNFSF10,UBD	
FOXL2		transcription regulator		-0.256	0.0229	ATF3,FOS,NFATC2,OSR2,PTGS2,RGS2,RSPO3,SMAD6,SPRY1	
E2F8	-2.596	transcription regulator			0.0231	CCNA2,CCNB1,CDC6	
RARRES3		enzyme			0.0231	CXCL10,ISG15,UBD	
HTATIP2	-6.457	transcription regulator			0.0231	MMP2,SOX2,SPP1	
mir-185		microrna			0.0231	AR,FDFT1,HMGCR	
STK4		kinase			0.0231	FAS,FOXO1,PROS1	
ERBB4		kinase			0.0231	BRCA1,CXCL12,PGK1	

USP18		peptidase			0.0231	IFITM3,OAS1,TNFSF10	
SCUBE3		other			0.0231	MMP2,SERPINE1,SNAI1	
ITGAL		transmembrane receptor			0.0231	HEY1,ITGB2,NOTCH1	
PROX1		transcription regulator			0.0231	HEY1,NRP1,PCK2	
MIF		cytokine		1.187	0.0239	FOS,MMP2,MMP3,TIMP1	
KDM5A		transcription regulator		0.762	0.0239	COL1A2,MCM2,MCM3,TERT	
MUC4		other		0.038	0.0239	CDH2,KRT18,SNAI1,TWIST2	
NFYB		transcription regulator			0.0239	CCNB1,IGF1,TOP2A,VWF	
ARNT		transcription regulator			0.0239	BNIP3,CA9,CLDN1,PGK1	
PTHLH		other			0.0239	CDC25B,ITGA6,PLAU,SERPINE1	
IL18	2.828	cytokine		0.185	0.0246	CXCL10,CXCL12,FAS,HAVCR2,MMP13,MMP15,MMP3,PTEN,PTGS2,TIMP1	
IL1B		cytokine		-0.883	0.0252	BMF,C3,COL1A1,CXCL10,EDN1,FAS,FGFR2,FLT1,IGF1,IL15,IL18,IL6R,MMP13,MMP3,NR1H4,NR4A2,NRP1,PLA2G4A,PTGS2,SCNN1A,SERPINE1,TACR1,TOB1,UBD,VEGFC	
RAF1		kinase		1.091	0.0257	CDC6,FAS,MMP13,MMP3,NGF	
Histone h3		group			0.0282	ADD2,ADD3,BCL2L11,CA9,COL4A6,CXCL10,DAB2IP,ENO3,FAM49A,FOXM1,MAGED2,MGMT,MYBL2,NDUFAF2,OSGIN1,PCBD2,PROS1,PTEN,PTGS2,RAB27B,RBL1,RGS10,SCD,SHANK3,TERT,TLR2,TNFSF10,TOP2A,ZNF423	
SOX11		transcription regulator		1.461	0.0283	ADAM9,AS3MT,EBF1,FAS,IGIP,NREP,SPIB,TIAM1,TP53INP1,TUBB3,YPEL1	
CD44		other		0.594	0.0283	ABCB1,ACTA2,BIRC5,CCNG1,FAS,FN1,MBNL3,PDCD4,PLAU,SBK1,SOX2	
EFNA1		other		-1	0.0288	BACH2,CTGF,ETV5,GATA6,ITGB4,KRT18,PKP1,PLAT,SLC20A1	
TLR3		transmembrane receptor		-0.492	0.0288	C3,CX3CL1,CXCL10,FOS,GBP4,IFIT3,IL15,ISG15,LIPA,OAS1,PIK3CD,PIK3R1,TNFSF10	
SRF		transcription regulator			0.0288	ACTG2,AKAP12,CTGF,DUSP5,FGF1,FOS,FOSB,ITGA1,NR4A2,PTGS2,RAI2,RND3,TAGLN	

PTGES		enzyme			0.029	BIRC5,EZR,FLT1,ITGA6,ITGB4,PPA1	
IgG		complex		0.258	0.0297	CEBPD,DDIT3,DSP,EDN1,EFNA3,EZR,GPRC5A,IFITM3,ISG15,KRT16,KRT18,PTGS2,RND3,SLC2A3,TOB1,TUBB2A	
PPRC1		transcription regulator		-0.707	0.0298	ATF4,CLDN1,DUSP5,GDF15,LAMB3,PTGS2,RND3,TMEM154	
IGFBP2		other		-0.524	0.0301	EPHA4,GDF15,POSTN,PTEN,RBMS3,SI,TGFB3	
DNAJB6		transcription regulator		1.091	0.0305	CDH2,KRT18,SPP1,VGF	
ADORA2A		g-protein coupled receptor		0.849	0.0305	CCL17,CXCL10,FOS,NR4A2	
SMARCE1		transcription regulator		0	0.0305	BRCA1,BRCA2,CCNB1,FOS	
ATG7		enzyme		-0.283	0.0305	ACTA2,BCL2L11,BMF,CDH2	
mir-181		microrna		-0.635	0.0305	AR,BCL2L11,GATA6,TIMP3	
PPP1R13L		transcription regulator		-1	0.0305	CLDN1,DSP,ITGA3,PKP1	
FGFR2	8.252	kinase			0.0305	COL1A1,FGFR2,SNAI1,SPP1	
E2F2	-6.449	transcription regulator			0.0305	CDC6,MYBL2,RBL1,UXT	
BSG		transporter		1.519	0.031	BCL2L11,CXCL12,IL18,MMP2,MMP3	
BMP2		growth factor		0.346	0.031	GADD45B,KLF9,RUNX3,SPP1,WNT4	
ATF2		transcription regulator		-1.067	0.031	ASNS,ATF3,DDIT3,FN1,PTEN	
CEBPB		transcription regulator		0.236	0.0313	ABCB1,ASNS,C3,DAPK1,DDIT3,FGFR2,GADD45A,IGF1,KRT18,MMP3,NFATC2,PTGS2	
JUN		transcription regulator		-0.099	0.0319	ABCB1,ASNS,CXCL10,DUSP5,GOT1,GSTP1,ITGB4,MMP13,MMP2,PARD6B,PDK1,PTGS2,SERPINE1,SOX2,SPP1,SULF2,SYK,VAV3	
MAP2K1/2		group		-1.782	0.0321	ATF3,BCL2L11,C3,CTGF,DDIT3,ELN,FOS,HERPUD1,KLF5	
WWC1	-12.4	transcription regulator			0.0326	CDH2,CTGF,FGF1	
TENM1		transmembrane receptor			0.0326	SEMA6A,SLC1A4,SOX5	

SUMO1		enzyme			0.0326	ABCB11,BIRC5,CXCL10	
UBE3A		enzyme			0.0326	ARC,TERT,TP53INP1	
GABPA		transcription regulator			0.0326	BRCA1,FAS,ITGB2	
MCAM	227.134	other			0.0326	ATF3,FN1,MMP2	
CYP1B1		enzyme			0.0326	MCAM,RAD51,TNFSF10	
WT1		transcription regulator		0.816	0.0327	ASNS,BTG2,CDC45,CSF1,FOXK2,GPKOW,NME1,NR4A2,ODC1,PCK2,SLC20A1,SLC2A3,TERT,VDR,WARS	
EGF		growth factor		0.484	0.0334	CCL17,CEBPD,DUSP5,EZR,FOS,NGF,PTGS2,SERPINE1,SNAI1,SPRY4	
HRAS		enzyme			0.0335	BIRC5,CXCL10,ELN,FAS,FOXO1,IGF2,ITGA6,SESN3	
ZBED6		other		0	0.0338	CRIM1,IGF2,MB21D2,TUBB3,UPP1,WWC1	
CCNK		kinase		-1.673	0.0338	BRCA1,PLTP,RPA2,TAF15,TIMELESS,UTP14A	
Gsk3		group		-1.982	0.0338	ATF4,DDIT3,FOSB,GDF15,HMGCR,NR4A2	
MECP2		transcription regulator			0.0338	ADAM12,BRCA1,EFNA5,GSTP1,MGMT,PTGS2	
TFRC		transporter		-1	0.0343	CCNA2,CCNB1,FOS,GADD45A,ITGB2,SULF2,TNFSF10	
RARA		ligand-dependent nuclear receptor		-1.886	0.0363	ABLIM1,BIRC5,CA12,CCNA2,CD14,CENPM,CENPN,CENPU,CLMN,EIF5A2,GDF15,GREB1,LIFR,MAD2L1,MAOB,PBK,PLK4,PLXNA2,SLC7A11,SPP1,THBD,TNFRSF11B,YPEL1,ZNF367	
NCF1		enzyme			0.0367	FN1,SERPINE1	
FGF16		growth factor			0.0367	MMP2,SNAI1	
ATP8B1		transporter			0.0367	ABCB11,NR1H4	
CERS2		transcription regulator			0.0367	CERS6,DDIT3	
GALNT6		enzyme			0.0367	CDH2,FN1	
LTB4R2		g-protein coupled receptor			0.0367	MMP2,NOX4	
LATS1		kinase			0.0367	CCNB1,CTGF	
RPS11		other			0.0367	PDCD4,PTEN	
CTNND1		other			0.0367	CDH2,SNAI1	
RPSA		translation regulator			0.0367	ITGA6,MMP2	
CDK5R1		kinase			0.0367	MMP2,SERPINE1	

GDF15	-13.916	growth factor			0.0367	GDF15,PLAU	
miR-192-5p (and other miRNAs w/seed UGACCUA)		mature microrna			0.0367	BIRC5,IGF1	
mir-320		microrna			0.0367	AQP1,BIRC5	
mir-32		microrna			0.0367	BCL2L11,BTG2	
NR1H2		ligand-dependent nuclear receptor			0.0367	ABCA1,C3	
HDAC9	13.18	transcription regulator			0.0367	CCNB1,KLF2	
MTTP	3.22	transporter			0.0367	GOT1,MTTP	
NKX2-3		transcription regulator			0.0367	ACTA2,AOC3	
MGAT3	24.526	enzyme			0.0367	CDH2,SNAI1	
CALR		transcription regulator			0.0367	COL1A2,FN1	
NRAS		enzyme			0.0367	NOL3,SESN3	
PTH		other			0.0367	CSF1,FOS	
GABPB1		transcription regulator			0.0367	BRCA1,ITGB2	
CRH		cytokine			0.0367	IL18,SLC2A3	
CANX		other			0.0367	ABCA1,NOX4	
ISG15	-2.628	other			0.0367	IFITM3,OAS1	
HBEGF		growth factor			0.0367	AR,NRP1	
RPS5		other			0.0367	PDCD4,PTEN	
PRKCB		kinase			0.0367	FN1,MMP2	
NF1		other			0.0367	GLI1,MAF	
PRKD1	-2371.19	kinase			0.0367	MMP3,PTGS2	
RPS18		other			0.0367	PDCD4,PTEN	

DYSF	8.555	other			0.0367	FN1,ITGB3	
NDFIP1		other			0.0367	LYN,PTEN	
SRC (family)		group		1.342	0.037	CDH2,FN1,ITGB3,MAOA,SNAI1	
ZEB1		transcription regulator		-1.187	0.037	ITGB4,KRT18,LAMC2,PLAU,SERPINE1	
FOXA1		transcription regulator		1.704	0.0372	ALDH6A1,COL18A1,CXCL10,GBP2,GREB1,KDM4B,KRT16,LYN,PALM2,SIAH2,TMEM74	
NOX4	1333.354	enzyme		1.925	0.0381	BCL2L11,BMF,MMP2,TGFA	
mir-27		microrna		0.132	0.0381	BIRC5,FLT1,IGF1,PKMYT1	
BRD7		transcription regulator		-0.283	0.0381	CCNG1,PIDD1,RAD51,VCAN	
SPHK1		kinase		-1.067	0.0381	CCL17,CTGF,PTGS2,SERPINE1	
Rb		group			0.0381	AURKB,DHFR,MMP3,TERT	
C3	12.773	peptidase			0.0381	ACTA2,C3,FN1,THBD	
HELLS		enzyme			0.0381	CCNA2,CCNB1,CDC6,RBL1	
Ap1		complex			0.0389	EDN1,EZR,F3,MMP2,NGF,SERPINE1,SPP1	
IL3		cytokine		-1.732	0.0391	CD14,FOS,MMP13,MMP3,RBM3,SEMA7A	
SMARCA2		transcription regulator			0.0391	DHFR,RBL1,RRM2,TAGLN,TBX2,VDR	
mir-122		microrna		1.741	0.0402	CCNG1,CS,MTHFD2,PTPN14,RBM3,RCC2,SLC1A5,SLC7A1,TGM2,VAV3,WARS,YARS	
SPP1	-6.743	cytokine		0.41	0.0403	BCL2L11,CDH2,CXCL12,DSP,FN1,KRT18,MMP2,PLAU,SNAI1,TM7SF2	
PRKCA		kinase		-0.156	0.0403	ARHGDIB,FN1,HEY1,IFIT3,INSIG1,MMP3,PTEN,PTGS2,SERPINE1,TGFB2	
POU2F2		transcription regulator		-0.333	0.0403	EBF1,GATM,MCM7,MVK,PFKFB4,PRR7,SCD,SPP1,TERT,TNFAIP2	
ERG		transcription regulator	Activated	2.333	0.0434	AR,ARHGEF2,AXIN2,CAMK1D,CCNA2,DNMBP,DOCK10,ELMO1,FLT1,HERPUD1,NLGN1,ORC6,PLAU,PTPN22,RCAN2,RHOBTB1,RHOJ,SLIT2	
Nr1h		group		1.356	0.0437	ABCA1,ABCG1,ACSL3,C3,PTGS2	
mir-15		microrna		0.672	0.0437	AR,CHEK1,ISG15,OAS1,RECK	
FOXP3		transcription regulator			0.0437	BRCA1,IKZF2,MAF,NFIL3,PDCD4	

ICAM1		transmembrane receptor			0.0437	FOS,HEY1,NOTCH1	
SH3KBP1	-2.862	other			0.0437	FN1,SERPINE1,SNAI1	
PRAME		other			0.0437	CYP26B1,IL13RA1,TOP2A	
IGFBP5		other			0.0437	F3,IRS1,SERPINE1	
ITGA6	-4.949	transmembrane receptor			0.0437	ENPP2,ITGA3,ITGB4	
FOXC2		transcription regulator			0.0437	CDH2,FN1,SNAI1	
EIF3E		other		0.52	0.044	COL18A1,COL5A1,MAD2L1,PAPD7,PLAU,RAD54L,SNAI1	
TGM2	-3.309	enzyme		1.753	0.0465	BNIP3,BTG2,C3,CD74,CXCL10,CYP4F3,DAAM2,GM2A,IFIT3,ITGA6,ITGB3,KCNQ5,LSP1,MERTK,MMP2,MPP6,OAS1,RTKN2,SEMA7A,SIRPA,SLC16A13,SPP1,TIMM8A,TNFAIP2	
ETS2		transcription regulator		1.123	0.0466	BRCA1,MMP3,SYK,TERT	
SLC29A1		transporter		1	0.0466	CCNG1,FAS,GADD45A,TP53INP1	
NONO		other		0	0.0466	PDE1B,PDE2A,PDE3B,PDE4B	
F2		peptidase		-0.038	0.0466	CTGF,FN1,PTGS2,TNFSF10	
Growth hormone		group		1.667	0.0479	CISH,CX3CL1,DDR1,FZD5,PDK4,PIK3C2B,TGFB3,TNFSF10,TXNIP	
IL7R		transmembrane receptor			0.0479	AHR,BEND5,DAPK1,FAM49A,FBXL16,IL7,MMRN2,REEP1,ZNF750	
VHL		transcription regulator		-1.993	0.0591	CA12,CDH2,MAD2L1,NOX4,TGFA	
IL12 (complex)		complex	Inhibited	-2.41	0.0614	FOS,HAVCR2,IL18RAP,ITGB2,MMP2,PLAU,TIMP1	
ITGAV		transmembrane receptor		1.981	0.0667	COL1A1,COL1A2,ITGB3,MMP2	
SMARCB1		transcription regulator		1.948	0.0871	ABCB1,CCNA2,CDC6,CSF1,OAS1	
UHRF2		enzyme	Activated	2	0.132	BCL2L11,FHDC1,FOXO1,MOAP1	

CTLA4		transmembrane receptor	Activated	2	0.148	CCNA2,KPNA2,SLC7A5,TOP2A	
ADIPOQ		other		-1.987	0.148	FN1,PTGS2,TIMP1,VEGFC	
CST5		other	Activated	2.548	0.154	ABLIM1,ACAT2,AKAP12,ARHGEF2,ATP2C2,C17orf49,CEMIP,COL12A1,DDX21,DSP,EZR,FHL1,GPRC5A,KDM5B,MAK16,NAV3,NHP2,NR2F1,NRP1,OTUD4,RBM3,RBP1,SEPT3,SLC7A11,SLC9A3R2,UCHL5,UTP14A,VCAN	
PELP1		other	Activated	2.236	0.174	COL12A1,HS3ST5,MRC1,PSAT1,RBP1	
LONP1		peptidase	Activated	2.813	0.178	ALDH1L2,GARS,GPT2,HSPA9,MARS,MTHFD2,PTGS2,SARS	
EIF2AK2		kinase		-1.969	0.179	ATF4,BRIP1,CEBPD,DDIT3,ISG15,NNMT,OAS1	
CDH1		other	Inhibited	-2.201	0.188	BIRC5,CCL17,CDH2,EIF4EBP1,MMP3	
HSF1		transcription regulator		1.778	0.223	ABCB1,BAG3,CDC6,CKS2,COL18A1,CSRP2,HSPA4L,KNTC1,MAPT,NFATC2,PGK1	
OSM		cytokine		1.975	0.234	FOS,IL6R,LIFR,OSMR	
CCL5		cytokine	Inhibited	-2.236	0.25	AHR,ALCAM,ATF4,HMGA1,SQLE	
miR-483-3p (miRNAs w/seed CACUCCU)		mature microrna	Activated	2.229	0.266	ASH2L,BRCA1,HNRNPA0,RAN,SLC7A1	
mir-8		microrna		-1.951	0.29	ADAM12,PTEN,SNAI1,TGFB2	
