# Supplementary material for: The impact and outcomes of cancer-macrophage fusion
Source: BMC Cancer. 2023 Jun 1;23:497. doi: 10.1186/s12885-023-10961-9 (PMC10236829; doi:10.1186/s12885-023-10961-9)
Supplement: Supplementary file 5 — Supplementary Material 5 [file 12885_2023_10961_MOESM5_ESM.pdf]

Supporting information

S3 Table. Upstream regulatory analysis of D2 hybrid cells vs. D3 hybrid cells. The RNA-seq data analyzed by Ingenuity Pathway Analysis (QIAGEN).

© 2000-2018 QIAGEN. All rights reserved.							
Upstream Regulator	Expr Fold Change	Molecule Type	Predicted Activation State	Activation z-score	p-value of overlap	Target molecules in dataset	Mechanistic Network
ERBB2		kinase	Activated	4.593	6.33E-22	ABL1,ADAM19,AKT1,ANGPTL4,BARD1,BIRC5,BMP1,BRIP1,BUB1,BUB1B,CCNA2,CCNB1,CCND1,CCND3,CCNE2,CDC14B,CDC20,CDC25A,CDC25B,CDC42BPG,CDC42EP3,CDC42SE1,CDC45,CDC6,CDCA3,CDCA5,CDCA8,CDH11,CDK1,CDT1,CEBPB,CENPA,CENPE,CHD1L,CHD7,CKS1B,CKS2,COL1A1,COL5A1,COL6A3,COL7A1,DHFR,E2F1,E2F2,E2F3,E2F7,E2F8,EGFR,ESPL1,FN1,FOXO1,FSCN1,FSTL3,GINS1,GINS3,GINS4,HES1,HEY1,ID1,IL6R,IL6ST,ITGA5,JUNB,KDM5B,LAMC2,LIG1,LIG3,LTBP2,LTBP3,MCM10,MCM2,MCM3,MCM4,MCM5,MCM7,MKI67,MXD3,MYBL1,MYBL2,NCAPD2,NCAPG,NEDD9,NET1,ORC1,ORC2,ORC6,PCNA,PDLIM4,PGK1,PLAU,POLA1,POLA2,POLB,POLE2,POLI,POLQ,POLR2A,POLR2G,POLR2L,POLR3H,PRC1,PTGS2,PTPRK,RAD51AP1,RFC3,RFC4,RHOD,RPA2,RRM2,SDC1,SMC2,SNAI2,TAGLN,TOP2A,TOPBP1,TYMS,VEGFA,XBP1	575 (18)
TGFB1	1.693	growth factor		-1.837	1.72E-15	ABL1,ACTA2,ADAM19,ANGPTL4,ARHGEF2,ASPN,BARD1,BCL2L11,BIRC5,BMP1,BRIP1,BUB1,BUB1B,C9orf3,CCNA2,CCNB1,CCND3,CCNE2,CDC20,CDC42SE1,CDH11,CDK1,CDT1,CENPA,CENPE,CITED2,CKS1B,CKS2,COL1A1,COL1A2,COL3A1,COL5A1,COL6A3,COL7A1,E2F1,ESPL1,FBN1,FLT1,FN1,FOXO1,FSCN1,FSTL3,GATA3,GLI1,HAS2,HBEGF,HEY1,ID1,IGF1,ILK,IRS1,ITGA1,ITGA3,ITGA5,ITGA6,ITGAV,ITGB2,JUNB,JUND,KDM5B,LAMC2,LTBP1,LTBP2,LTBP3,MKI67,MMP2,MMP9,MXD3,MYBL1,MYBL2,NCAPD2,NCAPG,NEDD9,NET1,NRP1,ORC1,PDGFA,PDLIM4,PLAU,PLOD2,POLE2,PRC1,PRKCA,PTGS2,PTP4A3,PTPRK,RAD51AP1,RFC4,RHOD,SCD,SDC1,SEMA3A,SIX1,SMC2,SNAI2,SPARC,SPHK1,TAGLN,TBX2,TFAP2A,TGFA,TGFB1,TGFB1I1,TGFBI,TGFBR2,TIMP1,TIMP3,TNC,TNFRSF12A,TOP2A,TWIST2,VCAN,VEGFA,VIM,WNT5A,XPC,XYLT1,ZFP36,ZFYVE9	417 (14)

TP53		transcription regulator	Inhibited	-3.37	8.49E-14	ABCB1,ACTA2,ADA,AKT1,AMOTL2,APAF1,ARHGEF2,ARPC1B,ATG4A,AURKA,AURKB,AXIN2,BAK1,BCL2L11,BIRC5,BLZF1,BNIP3,BRCA1,BTG1,BUB1,BUB1B,CAMLG,CASP1,CASP4,CAT,CAV1,CCNA2,CCNB1,CCND3,CCNE2,CDC20,CDC25A,CDC25B,CDC6,CDK1,CDT1,CEP55,CHEK1,CHIC1,CHUK,CITED2,CKAP2,CMBL,CPOX,CSMD3,CYFIP2,CYP26B1,DBF4,DCLRE1A,DDIT3,DDIT4,DEK,DHFR,DLX1,DNMT1,DUSP4,DUT,E2F1,EGFR,EGR3,ENSA,FDPS,FDXR,FEN1,FKBP1B,FOSL1,FOXO1,FUBP1,GADD45A,GLB1,GLIPR1,GSR,H2AFX,HK2,HMGCR,HMGCS1,HSPA1L,HSPA4L,ID1,IGF1,IGF1R,IGFBP7,IL7,IRS1,ISCU,JUNB,KIF23,KIF24,KRT8,LATS2,LIMK2,LTBP1,MAD1L1,MAD2L1,MAP2K4,MAP2K6,MBNL2,MCM2,MCM3,MCM4,MCM7,MDM4,MMP2,MMP9,MST1,MYBL1,MYO6,NCAPG,NDRG1,NINJ1,NUP153,OAS1,P2RX4,PALLD,PANK1,PARD6B,PBK,PCBP4,PCNA,PDCD6IP,PDE4B,PDK1,PFKM,PGM3,PHGDH,PHLDB3,PIM1,PLK2,POLE2,PPP3CA,PRC1,PRKAB2,PRKCB,PRKD1,PSEN2,PSTPIP2,PTP4A1,PTP4A3,PTPN1,PTPN6,RAD17,RAD23A,RAD54B,RECQL4,RFC3,RFC4,RPS25,RPS6KA1,RRAD,RRM2,SCRIB,SESN1,SFN,SIAH1,SLC16A1,SLC19A1,SMC2,SMC3,SMC4,SNAI2,SNRK,SORBS1,SOX2,SQLE,SSPN,STMN1,TAGLN2,TANK,TCEA3,TGFA,TGFB1,TGFBR2,TJP1,TNFRSF10A,TNFRSF9,TOP2A,TOP2B,TPD52L1,TPX2,TRAF1,UBE2B,UBE2C,UIMC1,UNC5B,VCAN,VEGFA,XPC,XRCC5,YES1	336 (10)

E2F1	2.068	transcription regulator		0.045	9.01E-14	ABCG2,ACTR1A,APAF1,ATG14,AURKB,BCL2L11,BIRC5,BRCA1,CASP7,CBX5,CCNA2,CCNB1,CCND1,CCND3,CDC20,CDC25A,CDC6,CDK1,CRADD,CTNNBIP1,CWC27,DBF4,DHFR,DUSP1,DUSP10,DUSP4,DUT,E2F1,E2F3,EYA2,EYA4,FANCD2,FEN1,FGFR2,FLT1,GINS1,GRAP,HIST1H2AC,HIST1H2BJ,HIST2H2AA3/HIST2H2AA4,ID3,IGF2,ILK,JMY,KIF23,MAF,MAP3K5,MCM10,MCM3,MCM5,MYBL2,PCNA,POLA1,POLA2,PPP1R13B,PPP1R8,PPT2,PSAT1,RACGAP1,RAD51,RAD54L,RBBP8,RBL1,RFC3,RFC4,RPA3,RRM2,SMC4,TOP2A,TOPBP1,TRAF3,TYMS,UNG,UXT,XPC,YBX3	343 (9)
E2F4		transcription regulator			1.95E-13	ANLN,ASF1B,ATRX,AURKB,BARD1,BRCA1,CBX5,CCNA2,CCND1,CCNE2,CDC25A,CDC6,CDK1,CENPA,CENPE,CHEK1,CKS1B,CKS2,DBF4,DHFR,DUT,E2F1,E2F2,E2F3,EIF2B2,FEN1,GINS1,H2AFX,H2AFZ,HIST1H2AC,HIST2H2AA3/HIST2H2AA4,ID3,KIF4A,MAD2L1,MCM10,MCM3,MCM5,MKI67,MRTO4,MYBL2,ORC1,PCNA,PLSCR1,POLA1,POLA2,PPP1R13B,PPP1R8,PRC1,PSAT1,RAD51,RAD51AP1,RAD54L,RBBP8,RBL1,RFC3,RFC4,RPA3,RRM2,SMC2,SMC4,TOP2A,TOPBP1,TTK,UNG,UXT,XPC	
MITF	2.228	transcription regulator	Activated	4.214	6.28E-13	ALCAM,APEX2,ATRX,AURKB,BRCA1,C1orf74,CCNB1,CCNF,CCNG2,CDC25B,CDCA3,CDCA8,CDT1,CENPH,CENPM,CEP55,CHAF1A,CHTF18,COL1A1,ESPL1,FANCA,FBXO32,FMOD,ITGA3,KIF20A,KIF4A,KIFC1,LIG1,MCM2,MCM4,MCM5,MITF,MMP14,NCAPD2,NCAPD3,ORC6,POLE2,POLM,RAD51D,RECQL4,RMI1,RPA3,SOX9,TACC3,TPX2,TXNIP,TYR,UBE2C,UIMC1,UPP1	

AR		ligand-dependent nuclear receptor	Inhibited	-2.192	3.85E-12	ABCC4,AKT1,BIRC5,BUB1,BUB1B,CAST,CAV1,CCNA2,CCND1,CDC25B,CDCA5,CDK14,CDT1,CEBPB,CENPE,CFLAR,CHTF18,CIT,CTSO,CXCL12,DEGS1,DHCR24,EBP,EGFR,ENO2,ERN1,ESPL1,ETS2,FEN1,FKBP5,HMGCR,HSPH1,IGF1,IGF1R,IL1R1,ITGA6,KIF2C,KNTC1,KPNA2,LMOD1,LRIG1,LRRK1,MAD2L1,MAOA,MCM2,MCM4,MRAS,MSMO1,MTHFD2,MYOM1,NUPR1,NUSAP1,P4HA1,PDIA5,PKMYT1,PLOD2,PRKCA,PROS1,RASSF3,SLC43A1,SLC7A11,SNAI2,STAT1,TGFB2,TGFBR2,TNFRSF12A,TPD52,TPD52L1,TPX2,UBE2C,VCAN,VEGFA,XBP1	516 (20)
NUPR1	1.915	transcription regulator		-1.646	3.09E-11	ACAD10,ADCY9,AGRN,ALG8,ANGEL1,ANK3,ARHGAP11A,ATAD5,AURKA,AVPI1,B3GAT3,BNIP3,BRCA1,BRCC3,BTG1,BUB1,BUB1B,C3orf58,C8orf58,CCDC134,CCNA2,CCNF,CD68,CDCA3,CDCA8,CEBPB,CENPI,CERK,CHUK,CITED2,CKAP2L,COL1A2,COL3A1,CYB5B,DAGLA,DDIT3,DDX46,DEPTOR,DHCR24,DHTKD1,E2F8,ELL2,ENO2,ERCC6L,ESPL1,FAM114A1,FAM162A,FANCD2,FGF1,FOXO3,FUT11,GAB2,GABBR1,GADD45A,GBP2,GCH1,GCNT2,GINS1,GMEB1,GPCPD1,GTSE1,HBEGF,HIVEP3,HJURP,HK2,HSPA2,IGF1R,IL13RA1,IL6R,ISCA1,KANK2,KBTBD7,KDM3A,KIF11,KIF18A,KIF20A,KIF23,KIF2C,KIFC1,KLF4,LRP8,MAGI1,MAN2B2,MCM10,MKI67,MMS22L,MRS2,MTFR2,NAAA,NDRG1,NFIL3,NR1D1,NSL1,P4HA2,PCTP,PDK1,PEA15,PHLDA1,PIK3R1,PIM1,PLK3,PM20D2,PMPCA,PODNL1,POLA2,POLE2,POLQ,PRELID2,PRKACA,PXDC1,RAD51,RANBP6,RASAL2,RCN2,RMI1,RNF19B,RNFT2,SAMD4A,SAT1,SHCBP1,SHOX2,SHROOM3,SIPA1L2,SLC16A6,SLC25A12,SPDL1,SPTSSA,STIL,SYTL2,TANGO6,TESK1,TFAP2A,TOB1,TRIB1,TRIB3,UNC5B,UPP1,VPS45,XBP1,XRCC2,ZBTB34,ZDHHC13,ZFAND2A	

MGEA5		enzyme		0	1.38E-10	ADAM19,AIFM1,AIFM2,ARHGAP29,CAV1,CCND1,CCNG2,CD302,CD99L2,CDK5,CEACAM1,CIAPIN1,CLDN1,CPE,CYFIP2,DAB2,EFNB1,EFNB2,ELK1,EPHA4,EPHB3,FDPS,FERMT2,FGFR1OP,FLNA,FLNB,FN1,FSCN1,FZD6,GADD45A,GALK2,GPER1,GPT2,HIPK2,HIVEP2,HMGCR,HMGCS1,HSF1,IGF2,IL17RD,IL6R,ITGB7,ITPKB,KREMEN1,LRIG1,MCAM,MED14,MMP14,MSMO1,NSDHL,PDCD6IP,PDK1,PECR,PFKM,PHKA2,PLAU,PLIN3,RAB27A,RAP2A,S100A3,S100A6,SERPINB9,SREBF2,TCF19,TFDP1,TGDS,TGFA,TGFB1,TGFBR2,TGFBR3,TIMP1,TIMP3,TNC,TPD52L1,TRADD,TSPAN5,UXT,VIM	223 (5)
RABL6		other	Activated	5.396	4.6E-10	AURKB,BUB1,BUB1B,CCNA2,CCNB1,CCNE2,CDC25A,CHEK1,CKS1B,DUT,EZH2,FEN1,FERMT2,H2AFX,HBEGF,KIF23,MAD2L1,MCM10,MCM2,MCM5,MCM7,MELK,NCAPG,PBK,POLA1,POLE2,PRC1,RAD54B,RFC3,TOP2A,TPX2,TTK,UBE2C	310 (7)
AREG		growth factor	Activated	2.917	8.3E-10	ARHGAP11A,AURKB,BIRC5,BSG,C3,CCNA2,CCNB1,CCND1,CCNF,CDC20,CDC45,CDCA8,CENPA,CPEB2,FOXM1,HIST1H2AC,HIST1H2BK,HIST2H2AA3/HIST2H2AA4,HJURP,IQGAP3,ITGB8,KIF20A,MKI67,MMP15,MYBL2,PCDHGA11,PLAU,PRC1,PTGS2,RRM2,SLC36A1,TOP2A	
FSH		complex		1.884	1.3E-09	ACP2,ACTA2,ACTG2,ADCY7,ADCY9,AMOTL2,ATP9A,AXL,BAD,BDNF,BMPR1A,BNIP3L,CAMK2G,CARD10,CASP4,CDK14,CDK16,CFLAR,CHUK,CREBL2,CREBZF,CTNND1,CYFIP2,DAB2,DUSP1,DUSP9,EFNA1,ELL2,FDXR,FKBP5,FLNC,FOXK2,GATA6,GCLC,GEM,GOT1,GPRC5A,GRK5,HK2,ILK,ING1,ISG20,ITGA3,KRT18,MAP3K5,MMP2,MMP9,MSMO1,P4HA2,PDXK,PGK1,PHKA2,PI4K2A,PLIN3,PPIH,PPP2R5B,PRKD1,PRKX,PSIP1,PTGS2,PTP4A1,PTPN1,PTPRF,RAB27A,RAD17,RANGAP1,RASAL2,RGS4,RGS7,SGK1,SMAD1,SMAD5,STAR,STAT1,STIP1,STK24,TFRC,TGFBR3,TLK1,TOB1,TRIB1,UPP1,VCL,VEGFA,VGF,YBX3	

KDM5B	-4.55	transcription regulator		-1.088	3.02E-09	AURKA,BRCA1,BUB1B,CAV1,CCNB1,CCND1,CDCA3,CDK1,DDIT3,DHCR24,DNAL4,E2F1,FBXO5,FHL1,GADD45A,HSD17B8,IARS2,KIF2C,LGALS3BP,LRCH4,MCAM,MCM2,MCM3,MIA3,NCAPH,NDE1,NEDD9,PBK,PDE3B,POLB,PPOX,PSIP1,RECQL,REEP1,SAT1,SMOX,SOX9,SPTSSA,SWAP70,TMEM14A,TOP2A,TTK,TUBB2A	
Lh		complex		0.631	4.05E-09	ACP2,ACTA2,ACTG2,ADCY7,ADCY9,ATP9A,AXL,BAD,BNIP3L,CAMK2G,CARD10,CASP4,CDK14,CDK16,CFLAR,CHUK,CREBL2,CTNND1,CYFIP2,DAB2,DUSP1,DUSP9,EGFR,FDXR,FKBP5,FLNC,GEM,GJA1,GRK5,HK2,ILK,ITGA3,KRT18,MAP3K5,MMP2,MMP9,MSMO1,P4HA2,PDXK,PGK1,PHKA2,PI4K2A,PLIN3,PPIH,PPP2R5B,PRKACA,PRKAR2A,PRKD1,PRKX,PSIP1,PTGS2,PTP4A1,PTPN1,PTPRF,RAB27A,RANGAP1,RASAL2,RGS4,RGS7,SGK1,STAR,STAT1,STIP1,STK24,TLK1,TRIB1,UPP1,VCL,VEGFA,YBX3	
TP63	7.376	transcription regulator	Activated	2.289	4.48E-09	ADA,AKT1,APAF1,ATG4A,BAD,BCL2L11,BRCA1,CAST,CCNA2,CCND1,CCND3,CDK1,CITED2,CKS2,COL4A1,COL5A1,DLX1,DNMT1,DUSP10,DUSP6,EGFR,ENSA,F2R,FBN1,FDXR,FGFR3,FN1,FOSL1,FST,FUBP1,GADD45A,HBEGF,HES1,HIRA,HK2,HOXC4,ID3,IER3,IGFBP6,IGFBP7,INHBA,ITGA3,ITGB8,KIF23,LYN,MAD2L1,MCM10,MFGE8,PCNA,PLAU,POSTN,PRPF4,PRPF8,RACGAP1,RAD51,RAD9A,RBBP8,RCC1,REXO4,SFN,SNAI2,SSPN,TAGLN,TGFB1,TGFB2,TGFB3,TGFBR2,TINAGL1,TIPIN,TNC,TNFRSF10A,TP63,VIM,WNT4,WNT5A	444 (16)
FOXM1	2.444	transcription regulator	Activated	3.484	7.07E-09	ATF2,AURKB,AXIN2,BIRC5,BUB1B,CAV1,CCNA2,CCNB1,CCND1,CCNE2,CDC20,CDC25A,CDC25B,CDCA8,CDK1,CENPA,CENPB,CENPE,CKS1B,CKS2,FOXF1,FOXM1,GTSE1,KIF20A,MMP2,MMP9,PCNA,PDGFA,PGK1,PLK4,PRC1,PTCH1,SOX2,STMN1	444 (14)

CDK4		kinase			4.48E-08	ATP6V0E1,AURKA,CDC45,CDC6,CDCA8,CENPH,CENPK,CENPN,CEP55,CIP2A,CPEB1,CPED1,DHFR,DONSON,E2F7,EIF5A2,ELN,ENPP2,FAM204A,FZD7,H2AFJ,HJURP,KDM6B,KIF11,KIF20A,KIF20B,KIF2C,KIF4A,MAGI2,MBLAC2,MCM7,MED30,MELK,MFSD6,MORC4,MTFR2,MTMR1,MYBL1,NCAPH,PBLD,PTBP3,THNSL1,TMEM219,TPX2,TRIP13,UTRN,ZFP28,ZNF367,ZNF423	
PGR		ligand-dependent nuclear receptor	Activated	2.137	6.15E-08	ABCG2,ACSL1,AMD1,ATP1B1,BTG1,CAPN2,CCND1,CCND3,CDC6,CDK1,CEBPB,CTBS,DDX21,FKBP5,FN1,GATA3,GOT1,GPER1,HBEGF,HES1,ID1,IER3,IL1R1,ITGA6,KLF11,KLF4,KLF9,KRT18,LAMB2,NDRG1,NEDD9,NET1,NQO2,P2RY2,PCNA,PFN2,PLAU,PTGS2,PTP4A1,SDC1,SGK1,SLC9A3R1,SNTB2,TGFB3,TM4SF1,TNC,UCK2,UGCG,VCAN,VCL,WNT4	425 (14)
E2F3	1.686	transcription regulator	Activated	3.69	9.94E-08	CA12,CASP7,CCNA2,CCNB1,CD9,CDC25A,CDC6,CDCA3,CDK1,CTNND1,E2F1,E2F2,ECE1,FAM46A,FST,HIST1H1C,HIST1H2BJ,HMGB2,IGF2,INHBA,KRT80,MAD2L1,MCM4,MYBL2,NCAPG2,PCNA,POLA1,PPP1R13B,PPP1R8,RALGPS2,RBL1,RRM2,SMG6,THBD,TIMP3,TM9SF2,TNFAIP2,TPX2,UBE2C,UXT,XPO4,ZNF76	
CCND1	1.661	transcription regulator		0.842	1.07E-07	AFAP1,ARHGEF2,ATP6V0E1,AURKA,CCNE2,CDC45,CDC6,CDCA8,CENPH,CENPK,CENPN,CEP55,CIP2A,CPEB1,CPED1,DNMT1,DONSON,DYNC1LI1,E2F1,E2F7,EIF5,EIF5A2,ENPP2,FAM120A,FAM204A,FLNA,FZD7,H2AFJ,HJURP,ITGAV,KDM6B,KIF11,KIF20A,KIF20B,KIF2C,KIF4A,LAMB2,MAGI2,MAP3K5,MBLAC2,MCM7,MED30,MELK,MFSD6,MORC4,MTFR2,MTMR1,MYBL1,NCAPH,PBLD,PCYT1B,PDLIM4,PTBP3,RAD51,RPL10,TALDO1,TGFBR2,THNSL1,TMEM219,TPX2,TRIP13,TUBA1B,TUBA1C,TYMS,UTRN,ZFP28,ZNF367,ZNF423	

estrogen receptor		group	Activated	2.346	2.32E-07	ABCG2,AGO1,AXL,C3,CA12,CAV1,CCND1,CCNE2,CD68,CDH11,CDH13,COL12A1,COL4A1,COL4A2,COL4A5,COL5A1,COL6A2,COL7A1,CXCL12,DCHS1,EGFR,FGF1,FGFR2,FGFR3,FLNC,FN1,GATA3,HBEGF,HSP90AA1,ITGA3,KRT18,KRT8,LAMC2,LOXL2,LY6E,LYN,MAN1A1,MMP14,MMP15,MMP9,MSN,NEDD9,PCDH7,PDGFA,PDGFC,PLAU,PLEK2,SAT1,SMAD1,SMN1/SMN2,SPARC,TGFA,TGFB1,TGFB2,TGFB3,TGFBR2,TGFBR3,TIMP1,TIMP3,TNC,VEGFA,VIM,WNT5A,XBP1	141 (5)
RBL2	1.533	other			2.66E-07	AURKB,BRCA1,CCND1,CDC25A,CDC6,CDK1,DHFR,DUSP10,E2F1,MYBL2,PCNA,PPP1R8,RBL1,RRM2,TYMS,UXT,XPC	
CDKN1A		kinase	Inhibited	-2.266	5.72E-07	APP,BIRC5,BRCA1,CCNA2,CCNB1,CCND1,CDC25A,CDC25B,CDK1,CHEK1,DUSP1,FOXM1,GLB1,IL1R1,KRT18,LGALS3BP,LIMA1,MAD2L1,MBNL2,MMP9,RBL2,SOX9,STMN1,TGFA,TOP2A	425 (15)
E2f		group	Activated	3.317	6.07E-07	CAV1,CBX5,CDC45,CDC6,CDK1,E2F2,EFHC1,FANCD2,GMNN,HIST1H2BJ,LIG1,MCM10,MCM2,MCM5,MYBL2,NASP,NUSAP1,POLA2,RAD51,RBL1,RECQL,RPA3,RRM2,TMEM126A,TMEM126B,TPX2,TTK,TYMS,UBE2C,UXT,YBX3	

TNF		cytokine		1.455	1.08E-06	A4GALT,ACTA2,ADAM8,APLN,BIRC5,BMP2,C3,CAT,CCL17,CCND1,CCND3,CDH11,CFB,CFLAR,CHST2,COL1A2,CXCL2,CYP27A1,DUSP1,DUSP10,EFNA1,EGFR,FGFR2,FLT1,FN1,FST,GADD45A,GCH1,GCLC,GCLM,GPR176,GSTM2,HBEGF,HDAC9,HIVEP1,IER3,IGF2,IKBKE,IL15,IL1R1,IL1RN,IL4R,INHBA,ITGA5,ITGAV,LAMA3,LAMC2,LBP,LTBP2,LYN,MAFF,MAP2K4,MCAM,MCM3,MFHAS1,MITF,MMP2,MMP9,MYO9B,NCK1,NCOA2,NEDD9,NFATC1,NFKB1,NGF,NINJ1,NR3C1,NR4A2,OAS1,OSMR,OTUD7B,PHGDH,PKMYT1,PLAU,PLOD2,PLSCR1,PPARD,PPP2R1B,PRSS23,PTGS2,RANBP9,RFTN1,RND3,RPS13,RRM2,SAT1,SDC1,SDC4,SERPINB1,SERPINB9,SLC7A1,SMAD1,SOX9,SQLE,STAR,STAT1,SYNPO,TGFA,TGFB1,TGFBR2,TICAM1,TIMP1,TIMP3,TJP1,TLR2,TLR3,TM4SF1,TNC,TNFAIP2,TPST1,TRAF1,TRAF3,UGCG,USP2,VCL,VEGFA,WISP1,WNT5A,XIAP,ZNF318	371 (13)
TP73		transcription regulator		1.059	1.96E-06	ABCB1,ACTA2,ADA,AUH,BIRC5,BLZF1,CAMLG,CCND3,CCNG2,CDK1,COL1A1,COL5A2,CXCL2,DCLRE1A,DDIT3,DHFR,DIMT1,DUSP11,FEN1,FGFR3,FKBP1B,FST,GAB2,GRK5,HAGH,HBEGF,HES1,HIVEP1,IL4R,IL7,KLHL21,LIG1,LTBP1,P2RX4,PIEZO2,PLK2,PROCR,PTP4A3,PTPN3,RAD17,SAT1,SFN,SNAI2,SNRK,STMN1,TCEAL1,TGFB1,TNFRSF10A,TNFRSF12A,TSPYL1,UBL3,VEGFA	403 (12)
SPDEF		transcription regulator	Activated	2.432	2.93E-06	CDH11,COL1A1,COL4A1,COL4A2,COL4A5,COL5A1,COL5A2,COL6A2,COL6A3,EGFR,ITGA3,ITGA5,ITGA6,LAMB2,LAMC1,PLAU,PRKCA,PTPRF,SDC1,SMAD1,SNAI2,TGFB1,TNC,VIM,WNT5A	178 (5)
RB1		transcription regulator		0.928	4.78E-06	BCL2L11,BIRC5,BRCA1,CASP4,CASP7,CBX5,CCNA2,CCNB1,CCND1,CDC25A,CDC6,CRADD,CWC27,DHFR,E2F1,FLT1,GMPS,HIST1H2BJ,ID1,IGF1,KPNA4,MAF,MYBL2,PCNA,PPT2,RAD51,RBL1,RRM2,TGFB1,TUBG1,TYMS,UXT,VEGFA	252 (7)
HELLS	2.642	enzyme	Activated	2.121	6.56E-06	CCNA2,CCNB1,CDC6,E2F1,MCM4,PCNA,RALGPS2,RBL1,SLC44A1,XPO4,ZNF76	

ESR1		ligand-dependent nuclear receptor	Activated	2.985	7.78E-06	ABCB1,ABCG2,BIRC5,BMP2,BRCA1,C3,CAPN2,CAV1,CCNA2,CCND1,CCNG2,CCNT2,CCPG1,CDK1,CEBPB,CENPA,CENPM,CENPN,CENPU,CP,CPE,CXCL12,DDIT4,DDX21,DECR1,DNAJC21,DUSP9,EFNA1,EGFR,EIF5A2,ENY2,FLCN,FMN1,FOXM1,FRAT2,FST,GATA3,GPER1,HELLS,HOXC6,HSF2BP,ID1,IER3,IGF1,IGF1R,INIP,ISG20,ITGA6,KIF23,LGALS3BP,LTBP1,MAD2L1,MINOS1,MKI67,NCAPG,NCAPH,NR1D1,PBK,PDZK1,PHLDB1,PLK4,POLA1,PROS1,PRSS23,PTGS2,RGS19,RGS3,SEMA3B,SESN1,SETD5,SGK1,SHANK2,SHANK3,SIAH2,SIPA1L1,SLC7A11,SMC4,SNAI2,SP110,TGFA,TM4SF1,TMEM74,TNFAIP2,TNFAIP6,TNFRSF10A,UBL3,VEGFA,VIM,WISP2,XBP1,ZNF367,ZNF703	311 (15)
HDAC1		transcription regulator		1.408	9.24E-06	APAF1,AXIN2,BRCA2,CCNB1,CCND1,CCNE2,CDC25A,CDC6,CDK1,COL1A1,COL1A2,DAB2IP,DHFR,FOXM1,GSTP1,ID1,MAD1L1,MMP9,PREX1,PTGS2,RBL1,RGS10,RRM2,SMN1/SMN2,SNAI2,SPP1,TGFBR2,TOP2A,TYMS	464 (14)
MAPK1		kinase		0.234	9.97E-06	ADAM12,ARHGAP11A,AURKA,BIRC5,BLZF1,C1S,CCNB1,CCND1,CCNF,CFB,COL7A1,CRYL1,DEPTOR,DSE,DUSP1,E2F1,ERAP1,FLNC,FN1,GLS,HOXA11,IFI16,IFIH1,ISG20,ITGAV,ITPR2,LGALS3BP,MITF,MKI67,MMP28,MYBL2,OAS1,PHF11,PLA2G4A,PLSCR1,PPT2,PTGS2,RAD54L,SHANK2,SLC4A7,SP110,SPRY2,SPRY4,STAT1,SUN2,TLR3,TRIM14,TRIM21,TRIM25,UBE2I,VIM,XIAP,ZNF184,ZNF318	
FOXO1	-1.68	transcription regulator		1.45	1.06E-05	ANLN,APAF1,BCL2L11,BIRC5,BRIP1,CCNB1,CCNG2,CDC42EP3,CDK1,DEPDC1,EGR2,FBXO32,FOXO1,GADD45A,IER3,IGF1R,IRS1,ITGA3,KIF11,KIF18A,MCM5,MMP9,MTTP,NCAPG,NUSAP1,PRC1,SGK1,SOX2,SPRY2,TGFB1,TXNIP,WNT4	368 (11)
26s Proteasome		complex		1.114	1.08E-05	ATG4A,ATG9B,ATP6V0E1,BAG3,BCL2L11,BRCA2,CCNA2,CCNE2,CD68,CDC25A,CDC6,CDK1,CENPE,CFLAR,CLCN7,EGFR,FOSL1,H2AFX,HBEGF,HMGB2,JUNB,KIF23,MAD2L1,MDM4,MICB,POLA1,PSMB3,PSMC2,PSMD2,RFC3,STAT1,TOP2A,WIPI1	445 (15)

GLI1	3.341	transcription regulator		0.358	0.000011	ABCG2,AKT1,CCND1,CMBL,CMPK2,COL1A1,DEGS1,EGR3,FGF1,FHL1,FUCA2,HPSE,IDS,IGFBP7,LMNA,MAN1C1,MMP2,MMP9,MRPS6,MTMR1,NQO2,PAPSS2,PDGFRA,PDZRN3,PSTPIP2,PTCH1,RAP2A,RGS10,RNASEL,RPS6KA1,SESN3,SPP1,TMEM79,TNC,TNFRSF9,VEGFA,ZC3HAV1L	
NR3C1	-1.618	ligand-dependent nuclear receptor		-0.475	1.16E-05	ABL1,ACAT1,AKT1,AMIGO2,ANGPTL4,ARRB1,ATP1B1,BAG2,BAG3,BARD1,BCL2L11,BNIP3L,BRCA1,BTG1,C1QTNF1,CAPN2,CARD10,CASP4,CASP7,CD38,CDC42EP3,CEBPB,CKAP2,CORO2A,DNER,DPP7,DUSP16,DUSP22,ENDOG,ERN1,F2R,FKBP5,FN1,FOXO1,FOXO3,GADD45A,GEM,GLIPR1,GULP1,IER3,IFIH1,IGF1R,IL15,IL15RA,IL18,IL3RA,ING1,INHBA,ITGAV,ITGB2,JUND,MAOA,MAP4K3,MDM4,NAIP,NFATC1,NFKB1,NME5,NR3C1,PAK1,PARP4,PDE4B,PEA15,PIK3R1,PIK3R3,PLA2G4A,PLK2,PPP2R1B,PPP3CA,PRKCB,PTGS2,PTK2B,RAD21,RELT,RTN4,SERPINB9,SGK1,SH3KBP1,SIAH1,SIAH2,SLC19A2,SMAD1,SNAI2,SPP1,STK39,THBD,TIMP3,TLR5,TNFAIP2,TNFAIP6,TNFRSF10A,TNFRSF12A,TRAF1,TRAF3,TRAF5,TRIB3,UNC13B,WDR31,WDR37,YWHAZ,ZNF346	
RBL1	2.39	transcription regulator			1.16E-05	AURKB,CCND1,CDC25A,CDC6,CDK1,DHFR,DUSP10,E2F1,MYBL2,PCNA,PPP1R8,RBL1,RRM2,TYMS,UXT	
CHEK1	1.904	kinase		0.306	1.62E-05	BCL2L11,CDC25A,CDC25B,E2F1,E2F3,H2AFX,MICB,PCNA,RRM2	344 (10)
ATF4	1.538	transcription regulator	Activated	3.369	2.11E-05	ASNS,CA9,CEBPB,CHAC1,DDIT3,DDIT4,FUT7,PHGDH,PSAT1,PSPH,PTGS2,SLC38A2,SLC7A11,VEGFA	
YAP1		transcription regulator		1.289	2.29E-05	AMOTL2,ANLN,AURKB,BCL2L11,BIRC5,CCND1,E2F1,EGFR,FOXM1,LATS2,MSLN,PTGS2,SLC2A3,SNAI2,SOX9,TAGLN,TP63,VIM	
Cg		complex		1.339	2.32E-05	ARHGAP22,BARD1,BMP2,CCNE2,CDC6,DUSP6,FST,GATA6,HAS2,HK2,ITGA1,ITGAV,MAF,MCAM,MCM10,MMP2,MRPS6,PHLDA1,PKIA,PLAT,PLAU,PTGS2,RECQL,RGS4,SLC20A1,SLC4A4,SPRY2,STAR,TIPIN,TM4SF1,UNG,UPP1,VEGFA,XIAP	227 (9)

Akt		group		0.796	2.33E-05	ABCB1,ACTA2,BIRC5,CCND1,CCND3,CEBPB,CFLAR,COL3A1,EZH2,FKBP5,FOSL1,FOXO1,FOXO3,GSR,IGF1R,IL1R1,MCAM,MMP14,MMP2,MMP9,MSI1,NET1,PDK4,PEA15,PTGS2,RDH10,SGK1,SLC4A7,SNAI2,TIPARP,TJP1,TNC	560 (16)
E2F8	1.942	transcription regulator			2.84E-05	CCNA2,CCNB1,CCND1,CDC6,E2F1,KIF23,RACGAP1	
HIF1A		transcription regulator		-0.973	4.21E-05	ABCF2,ANGPTL4,AURKA,AXL,BNIP3L,CA9,CAV1,CCND1,CEMIP,CLDN1,CTPS1,CXCL12,CYP4F3,EGFR,EGLN3,ENO2,ERGIC1,FHL1,FN1,FSCN1,GJA1,GPER1,HIST1H2BK,HK2,IGF1,IL15,ITGA1,ITGA5,ITGB2,KDM3A,LOXL2,MMP2,MMP9,NDRG1,NEK8,NOV,P4HA1,P4HA2,PDK1,PHLPP1,PLOD2,POMK,PRKCA,PTGIS,PTGS2,SDC4,SIAH1,SLC29A1,SOX9,SPHK1,SRD5A1,TAF9B,TFRC,TGFB1,TGFB3,TLR2,TLR6,TMEM79,VEGFA	448 (12)
FBN1	-5.647	other	Activated	2.396	4.45E-05	COL1A2,COL3A1,COL4A2,COL6A3,FBN1,LTBP1,LTBP3,TGFBR2,TIMP3	29 (2)
E2F6		transcription regulator	Inhibited	-3.317	5.12E-05	BRCA1,CBX5,CDC25A,CDC45,CDC6,E2F1,E2F2,GMNN,KDM3A,LIG1,MCM2,MCM3,MCM5,POLA2,RAD51,RAD51AP1,RBBP8,RECQL,RRM2,UXT	
mir-122		microrna	Inhibited	-2.029	6.31E-05	ACTR1A,CDA,CLIC4,CS,CSRP1,GALNT10,GNPDA1,GYS1,H1F0,LAMC1,LETM1,MTHFD2,NUDC,NUMBL,NUTF2,PLIN3,PTPN1,PTPN14,RAD21,RBM3,RCC2,SEMA4D,SEPT2,SF3B4,SLC7A1,TNPO2,VIM,WARS,YARS	
DUSP1	2.647	phosphatase		-1.849	6.77E-05	BMP2,COL3A1,DUSP1,DUSP6,EGFR,NRP1,PIK3R1,PLAT,PTGS2,PTPRK,THBD,TLR2,WISP2	213 (9)
WISP2	2.658	growth factor		0.726	9.44E-05	CLDN1,FN1,GATA3,IGFBP7,ITGA5,KLF4,KRT18,KRT8,LAMC2,PROCR,SNAI2,SPARC,TGFB1,TGFBR2,TGFBR3,VIM,XBP1	28 (2)

IL2		cytokine		1.342	0.0001	ADCY3,ADCY9,AHR,BMP2,CARD10,CASP1,CCND1,CCND3,CCNE2,CCNG2,CDC25A,CDC6,CDK5R1,CSF2RB,CTPS1,CXCL12,DUSP4,DUSP6,EEF1E1,ENPP2,EPHA4,FGFR2,FOXO3,GADD45G,HK2,IDI1,IER3,IL18RAP,IL2RG,ISG20,JUND,MAP2K6,PDE3B,PDE4B,PDGFC,PEA15,PHLDA1,PIK3R3,PIM1,POLE2,PRKX,PTCH1,RFFL,RFTN1,SERPINB9,SESN1,SESN3,SLC2A3,SPP1,STIP1,TFDP2,TGFB1,TNFRSF10A,TNFRSF12A,TNFRSF9,TRIB3,UCK2,UGP2,VEGFA	125 (3)
PDGF BB		complex		1.67	0.000104	CCND1,CCNO,CEBPB,DAB2,DUSP1,DUSP6,EGR2,EGR3,FOSB,GADD45A,GEM,IER3,JUNB,KLHL21,LMNA,NR4A2,PHLDA1,PIM1,PLAU,PLK2,RND3,SGK1,SLC2A3,SYK,TOB1,TRIB1,ZFP36	265 (12)
miR-296-5p (miRNAs w/seed GGGCCCC)		mature microrna		-0.97	0.000106	AKT1,COL1A1,HMGA1,LYPLA2,NUMBL,PCNA,SCRIB,TNC,VEGFA	
SIN3B		transcription regulator			0.000124	CCNG2,COL1A2,DDIT3,DHFR,FBXO32,HES1,RRM2,SRD5A1,TXNIP,TYMS	
SYVN1		transporter		-1.305	0.000143	ABCC4,ACP2,ADCY9,AHR,AMOTL2,APP,ASF1B,ATP1B3,AXL,CCND1,CDA,CRTAP,CTPS1,DAB2,DUSP1,ERCC6,GGCX,GPRC5A,IGF1R,ITGA3,ITGA6,ITGAV,LDHB,LGALS3BP,MCAM,NDFIP2,RAB34,RPL10,SCARA3,SLC16A1,SLC20A1,SLC2A3,SLC30A1,SLC39A10,SLC43A2,SLC4A7,TARS,TFRC,UNC13D,ZNF281	
E2F7	1.533	transcription regulator			0.000146	CCNA2,CCNB1,CDC6,E2F1,KIF23,RACGAP1	
SMAD3		transcription regulator		-0.228	0.000166	ACTA2,ASPN,C3,CCNG2,COL1A1,COL1A2,COL3A1,DEPTOR,FN1,FSTL3,GLI1,HAS2,HEY1,NET1,PTGS2,PTP4A3,PTPRK,SNAI2,SOX2,TIMP3,VEGFA,VIM,XIAP	182 (7)

WT1		transcription regulator		-0.32	0.000167	ASNS,BAK1,CDC45,CDC73,CHAF1B,CIRBP,CMPK1,CUL4B,EFNA1,FIBP,FOXK2,GPKOW,HBEGF,IGF1R,JUNB,JUND,MMP9,MTIF2,NCSTN,NDRG1,NQO2,NR4A2,ODC1,PHLDA1,SEC13,SLC20A1,SLC2A3,SNAI2,TFAP2A,TRIM21,TSPAN5,VEGFA,WARS,YBX1	
PRNP		other		-0.084	0.000186	ABCB1,AHR,APP,DAB2,ETS2,FOSL1,FST,GJA1,IGF2,IRS1,MMP2,SOX9,TLE1	
SMAD2		transcription regulator		-1.245	0.000186	ACTA2,BCL2L11,CCNG2,FN1,FSTL3,HAS2,MMP2,MMP9,NET1,SNAI2,SOX2,TIMP3,XIAP	
SP1		transcription regulator		1.922	0.00019	ACVRL1,ASNS,ATP6V0C,BIRC5,CAT,CAV1,CCND1,CDC42BPG,CDK1,CDKN2D,CEACAM1,CEBPB,CEBPD,COL1A1,DBF4,EGFR,F2R,FLT1,FN1,FOSL1,FOXF2,FOXM1,GJA1,GLTP,HAS2,HBEGF,HK2,HMGA1,HPSE,IGF1R,IGF2,IL15,ITGAV,KRT16,KRT18,LTBP3,MAOA,MAOB,MMP14,MMP2,NFKB1,PDGFA,PDK1,PIGM,PIM1,PLAU,POLB,PPARD,PREX1,PRKCA,PROCR,PTGS2,RBL1,SLC19A1,SLC22A4,SLC4A7,SOX9,SPP1,STAT1,TEAD1,TGFB1,TGFB2,TGFBR2,TLR2,TRIB1,UGCG,VEGFA,VIM	365 (11)
miR-122-5p (miRNAs w/seed GGAGUGU)		mature microrna		0.286	0.000193	BACH2,CS,DSTYK,EGLN3,ENTPD4,FUBP1,GALNT10,NCAM1,NFATC1,NFATC2IP,NUMBL,OSMR,PSME1,RAB11FIP1,RAB6B,SLC7A1,SLC7A11,TRIB1,TTYH3,VIM	
Mek		group		1.76	0.00021	ABCE1,ACAT1,AXL,BCL2L11,BIRC5,CCND1,CDK5R1,DDR2,DDX21,DNMT1,DSCC1,DUSP4,DUSP6,ETV5,FOSL1,GPER1,HAS2,ID1,IER3,ITGA6,LRP8,MAF,MAFF,MMP2,MMP9,NGF,PDGFRA,PHLDA1,PLCD1,PTGS2,RABGGTB,RND3,SLC16A6,SLC20A1,SMN1/SMN2,SPRY1,SPRY2,SPRY4,STON1,TFB2M,UNG,VIM	350 (14)
IGF1	-52.156	growth factor		0.052	0.000212	BAD,BAK1,BCL2L11,BIRC5,BMP2,CCND1,DDIT3,EFNB2,ELN,FN1,IGF1,IGF1R,IGF2,IRS1,ITGAV,PLAU,SFN,SLC20A1,VEGFA,VIM,XBP1,XIAP	472 (18)

Histone h4		group			0.000212	ABCB1,ADA,CCND1,CCND3,CDC25A,CDK1,CFLAR,DHFR,E2F1,FMR1,FOXM1,HAS2,HPSE,PREX1,PTGS2,RBL1,RPL12,RRM2,SMN1/SMN2,SPHK1,TGFBR2,TYMS	244 (6)
E2F2	13.207	transcription regulator			0.000224	CDC25A,CDC6,CDK1,E2F1,JMY,MYBL2,POLA1,RBL1,UXT	
ERK		group		1.488	0.000275	AXL,BCAR3,BCL2L11,CCND1,CDK5R1,COL1A1,CXCL2,DUSP1,EGFR,FOSL1,FOXO1,FST,GCLC,HBEGF,ITGAV,ITGB2,JUNB,MMP14,MMP2,MMP9,NDRG1,ODC1,PDK4,PPARD,PTGS2,SLC4A7,SNAI2,STAT1,TGFA,UGCG,VCAN,ZFP36	351 (14)
EPAS1		transcription regulator		-1.581	0.000316	ABCF2,ABI1,ANGPTL4,AXL,CA9,CAV1,CDC42EP5,CEMIP,CKMT1A/CKMT1B,CLDN1,EGFR,EGLN3,ENO2,FHL1,GJA1,GLS,KDM3A,LOXL2,NDRG1,NEK8,PRKCA,SLC29A1,SLC6A8,SOX9,SPHK1,TAF9B,UGP2,VEGFA,WISP2	
ERK1/2		group		0.172	0.000363	APH1A,APH1B,ARRB1,ASAH1,B2M,BCL2L11,BRCA1,C3,CAT,CCND1,CDC42EP5,CDK1,CEBPB,COL3A1,DDIT3,FGFR2,FKBP5,FN1,FOSB,FOXO1,HBEGF,HK2,ID1,IGF1R,IL17RD,JUNB,JUND,KCNH2,KIF2C,MAP3K11,MMP2,PCNA,PTGS2,SGK1,SOX9,SPRY4,TIMP1,VEGFA,WISP1	432 (15)
PPARG		ligand-dependent nuclear receptor		0.742	0.000365	ABCG2,ACSL5,ACTA2,ANGPTL4,APH1A,APH1B,APP,BIRC5,CAV1,CCND1,COL1A1,COL1A2,FN1,GPT,IGFBP6,KLF11,MMP9,PDK4,PTGS2,SDC1,SLC9A1,TGFBR2,VEGFA	287 (10)
UXT	2.6	transcription regulator	Inhibited	-2.401	0.000371	B4GALT1,BRIP1,BUB1,CCNA2,CDC6,CHEK1,ENO2,FKBP5,GTSE1,KRT18,NCOR1,SORD,TLR3,TTK	
PI3K (family)		group	Activated	2.263	0.000429	ACTA2,BCL2L11,BIRC5,CAT,CCND1,CCNG2,CFLAR,CXCL12,DDIT3,DDIT4,FBXO32,FOXO1,FOXO3,FTH1,GCLC,GCLM,GLI1,ITGA6,KLF2,MMP2,MMP9,PREX1,PTGS2,RDH10,SLC4A7,SNAI2,TJP1,TNC,TXNIP,VEGFA,XIAP	461 (16)
MAP2K1		kinase		0.348	0.000495	BCL2L11,DNMT1,DUSP1,DUSP6,FOSL1,JUND,MITF,MMP2,MMP9,NET1,PCNA,PLA2G4A,RAP2B,RRAGD,SNAI2,UNC5B	203 (9)

SMARCA4		transcription regulator		1.002	0.000533	ABCB1,ACSL5,AHR,ANGPTL2,ANO1,ARHGDIB,ASNS,BAK1,BEND5,BIRC5,CASP1,CCDC153,CD74,CDC25A,CDC6,CFP,CLK1,COL7A1,COTL1,CP,CXCR6,DHRS9,DLX2,DUSP4,DUSP6,E2F1,EFNB1,EMP3,EPHA4,FADS3,FKBP5,FLNB,FN1,GADD45A,GCLC,GSTO1,GSTP1,GULP1,ID3,IER3,IFI16,IFI30,IGF1,IGFBP7,IL15RA,INHBA,ITGA3,ITGA5,KDM6B,KIFAP3,KRT18,LAMA3,LMNA,LOXL2,MAFF,MAOB,MFGE8,MICB,MMP2,NFKBIZ,NRP1,OCIAD2,OXCT1,PDE4B,PDK1,PHLDB1,PKIA,PLAT,PLEKHG2,PLS1,POLB,PON3,PTP4A1,PTP4A2,RBL1,RBP1,S100A6,SEMA3B,SEMA7A,SERPINB7,SLC11A1,SOD3,SOX2,SPHK1,SPP1,SPRY2,STXBP6,SYK,TAGLN,TGFB2,TLR2,TMCC3,TMEM171,TNFRSF9,TRNP1,TWF1,UBD,UNC13D,USP24,VIM,WDR45,ZNF503	
MYC		transcription regulator		-0.384	0.00058	ABCC4,ALCAM,APEX1,AXL,C9orf3,CAV1,CCNB1,CCND3,CCNG2,CDC25A,CFLAR,CHEK1,COL1A1,CTDSP1,CYFIP2,CYTH2,DDIT3,E2F1,E2F2,FBXO32,FKBP5,FMOD,FOSL1,FOXM1,GADD45A,GCLC,GCLM,GJA1,GLS,GSR,HK2,HSP90AA1,HSPH1,ID1,ITGA1,ITGA3,MITF,MTHFR,ODC1,PDK1,PLAU,POLDIP3,PYCR1,RAD51,RCC1,RFFL,SAT1,SHMT2,SLC22A4,SOX9,SPP1,ST3GAL3,ST3GAL4,SUMO3,TMEM126A,TNFRSF10A,TXNIP,TYMS,UBE2C,UBE2I,UXT,VEGFA	108 (4)
SP3		transcription regulator		0.128	0.000582	ASNS,BIRC5,CCND1,COL1A1,COL1A2,EGFR,F2R,FLT1,FOSL1,GLTP,HAS2,IGF1R,IGF2,ITGB8,KRT16,MAOB,MMP2,NFKB1,PLAU,PREX1,PROCR,PTGS2,RBL1,SLC19A1,SLC4A7,STAT1,TGFBR2,VEGFA,XYLT1	334 (9)
ITGB1		transmembrane receptor		0.414	0.00067	COL1A1,FLNA,FOXO3,ITGA5,ITGAV,JUNB,MMP9,PLAU,PTGS2,TGFB1,VEGFA,VIM,WNT5A	424 (16)
MDM2		transcription regulator		1.703	0.000684	CCND1,CLIP3,E2F1,FCGRT,FOXO3,HIPK2,HIST2H2AA3/HIST2H2AA4,IGF1R,IGFBP6,IL1RN,LYZ,MDM4,NDUFA1,SFN,SPRY2,TIMP1	
Raf		group			0.000684	CCND1,DUSP4,DUSP6,ETV5,FOSL1,HAS2,IER3,IGF1R,MAFF,RND3,SLC16A6,SLC20A1,SPRY1,SPRY2,SPRY4,STON1	171 (8)

RNA polymerase II		complex			0.000687	B2M,BLNK,BRCA1,CA12,CASP7,CBX5,CCNB1,CCNE2,CDH11,CSDC2,CXCL2,GADD45A,GOT1,GPT,HAS2,HOXA11,ID1,IL1RN,JUNB,MCM3,MINOS1,MMP2,MMP9,MYBL2,PCNA,PDZK1,PKMYT1,POLB,POLR2A,PTGS2,RAD51AP1,RBBP8,RND3,RPL12,SFN,TGFB1,TOP2A	320 (7)
ZNF281	-2.085	transcription regulator	Activated	2.607	0.000724	FANCA,FANCD2,GADD45A,GADD45G,RAD9A,XRCC2,XRCC4	
ENG		transmembrane receptor		1	0.000724	BARD1,H2AFX,ITGA1,ITGA5,ITGA6,NTHL1,PLAU	86 (4)
L1CAM		other		-0.447	0.00074	FDXR,IDI1,MMP2,MMP9,SQLE	
ZMYND10		other		-1.408	0.00074	MMP14,MMP19,MMP2,MMP9,VEGFA	
Pkc(s)		group		0.103	0.000777	ACAT1,APP,ATF2,BCL2L11,DDIT3,DNAJC3,ERN1,FOSL1,GADD45A,GPER1,HMGCR,IGF2,JUNB,MAD1L1,MMP2,MMP9,PTGS2,STAR,VEGFA,XBP1,XIAP	236 (12)
Creb		group		-1.501	0.000781	BAK1,BDNF,CCND1,CEBPD,DUSP1,DUSP4,FOSL1,FSCN1,HAS2,HES1,IER3,MMP2,NEDD9,NR3C1,PTGS2,SOX9,STAR,TRAIP,VEGFA,WISP1	96 (4)
ITGB3		transmembrane receptor		0.132	0.000801	CDK1,CEACAM1,COL1A1,HAS2,ITGAV,MMP2,MMP9,SNAI2	281 (12)
PAK1	-1.505	kinase		0.064	0.000801	CCNB1,FN1,FOSL1,MMP14,MMP9,PFKM,TFPI,VEGFA	299 (12)
MXI1		transcription regulator	Inhibited	-2.137	0.000801	APEX1,CCNB1,FOXM1,IARS,ID1,MKI67,MTHFD2,SLC7A1	
NFYB		transcription regulator			0.000801	CCNB1,CDCA8,CDK1,COL5A3,FTH1,IGF1,TOP2A,VWF	
CST5		other		-0.97	0.000807	AGO1,ANK3,ANXA6,AP3D1,ARHGAP29,ARHGEF2,C17orf49,CAMK2G,CAV1,CBY1,CEMIP,COL12A1,DCP1A,DDX21,DECR1,DNAJB11,DOCK9,DRAP1,EIF2B2,EMP3,EVA1C,EXOC3,FHL1,GOLGA2,GPRC5A,GRAP,GTPBP10,ID3,KDM5B,KIF11,LAMC1,LIMD2,MAK16,MAP1S,MRTO4,MSN,MYL9,NCBP1,NHP2,NOL9,NR2F1,NR3C1,NRP1,NTHL1,OTUD4,PAPD5,PARP4,PIP4K2A,PPAN,PPIC,PRDX1,PRKRA,PRPF8,RBM3,RBP1,RRP15,SLC7A11,TBL3,TOMM22,TSPAN5,TXN,UTP14A,VCAN,VIM,VPS36,VTI1A,ZCCHC8,ZFC3H1	

SFN	5.496	other		-0.447	0.000983	FKBP5,IL1R1,KRT18,KRT8,NET1,PDK4,PKP3,SGK1,SNAI2,TJP1,VIM	
MAP2K1/2		group		1.284	0.00101	BCL2L11,C3,CCND1,CDK1,DDIT3,DUSP1,DUSP4,EGR2,ELN,FOSL1,H2AFX,HK2,MMP9,PCNA,PDGFA,PHLDA1,TGFB1,VEGFA,VIM	434 (15)
JUN		transcription regulator	Activated	2.217	0.00101	ABCB1,ASNS,AXL,CAPN2,CCND3,CDK1,DUSP1,DUSP6,FOSL1,FTH1,GCLC,GJA1,GOT1,GPT,GSTP1,ITGAV,ITGB8,JUNB,LTBP1,MMP2,MMP9,NFKBIZ,PARD6B,PDK1,PEA15,PTGS2,RASA1,SLC38A2,SNAI2,SOX2,SPP1,SYK,TGFB1,TNFRSF10A,VEGFA,VIM,XYLT1,ZFP36	407 (16)
EGF		growth factor		1.245	0.00125	CCL17,CEBPB,CEBPD,CFLAR,DUSP1,DUSP6,EGFR,MMP9,NGF,PSMB3,PSMB7,PTGS2,S100A10,SLC4A7,SNAI2,SPRY2,SPRY4,ST3GAL3,ST3GAL4,TGFBR2,VEGFA	342 (19)
EGR1		transcription regulator		0.95	0.00125	ACTA2,BCL2L11,CAV1,CDK5R1,COL1A2,FN1,FOSL1,GLI1,HBEGF,HPSE,IGF2,IL6R,NDRG1,PDGFA,SNAI2,VEGFA	97 (5)
CIP2A	1.606	other		-1.476	0.00128	ACTG2,CFB,CIP2A,CRLF1,E2F2,ENO3,GADD45A,GPNMB,HIST1H2BK,IER3,LXN,NPTX1,PDK4,PXYLP1,RHOC,RHOD,S100A16,SAT1,SCIN,SLC22A4	
FZD8		g-protein coupled receptor		-0.101	0.00128	ACTA2,COL1A1,FN1,TJP1,VCAN,VIM	
miR-210-3p (miRNAs w/seed UGUGCGU)		mature microrna		-0.186	0.00128	E2F3,EFNA3,FGFRL1,ISCU,NPTX1,PTPN1	
CTNNB1		transcription regulator		0.165	0.00134	ABCB1,ABCD2,AXIN2,BIRC5,CCND1,CDH11,CEACAM1,CNN2,DDIT3,ECM1,EGFR,ENO2,EPHB3,FN1,FOXQ1,GATA3,ID3,IL1R1,IRS1,ITGA1,KIF23,KIFC1,LAMC2,MMP14,MMP2,MMP9,PCCA,PDE4B,PIGC,PLAU,PPP3CA,PTCH1,RAD23A,SFN,SGK1,SOX9,TGFB1,TRAF1,UGCG,VCAN,VEGFA,VIM,WNT6	490 (18)

NCOA3		transcription regulator	Activated	2.778	0.00139	CCDC80,CCNA2,CCND1,CCNE2,CDC25A,CDC6,HES1,IGF1,MCM7,MMP9,PCNA,PSMA2,PTGS2	413 (17)
miR-17-5p (and other miRNAs w/seed AAAGUGC)		mature microrna		-0.69	0.00146	APP,BIRC5,BMP2,E2F1,H2AFX,ITGB8,PURA,TGFBR2	
N-cor		group			0.00146	AXIN2,CCND1,GSTP1,HES1,IGF1,INPP4B,PTGS2,SNAI2	
TCF4		transcription regulator		1.568	0.00152	AXIN2,BIRC5,CCND1,CDH17,FGF1,GLI1,IRS1,LAMC2,PLAU,SGK1,SOX9,SPP1,VCAN,WISP2	
NFE2L2		transcription regulator	Activated	2.031	0.00161	ATF4,BRCA1,CAT,DDIT3,FTH1,GCLC,GCLM,IL1RN,PHGDH,POMP,PRDX1,PSAT1,SHMT2,SNAI2,TALDO1	
EIF3E		other		-1.912	0.00161	ANGPT1,CCND1,CCNF,CDCA8,CMTM7,COL5A1,FARP1,LIG1,LIG3,MAD2L1,PAPD7,PLAU,RAD54L,SMC2,TNC	
CREB1		transcription regulator	Activated	2.537	0.00161	AURKA,BIRC5,CCNB1,CCND1,CDCA8,CEBPD,FLT1,FN1,FOSB,FUT7,HAS2,MCM5,MKI67,MYBL2,NR4A2,OPA3,PCNA,PLA2G4A,PTGS2,RAD54L	
CLDN7		other		-1.787	0.00163	ALDH3A1,ARHGDIB,B4GALNT1,C1S,C3,CA12,CD68,DPM3,FADS3,FOSL1,FOXQ1,GLS,GRINA,HELZ2,HIST2H2AA3/HIST2H2AA4,IGFBP7,LGR4,LIMA1,LTA4H,MMP2,MMP9,MRPL36,NSA2,PHGDH,PKMYT1,PRSS23,RPP40,SERPINB1,SERPINB7,SLC35B1,SLCO2A1,SYTL2,TINAGL1,WIPI1	
PRKCD		kinase		-1.641	0.00174	ARHGAP32,BIRC5,CCND1,CEMIP,COL1A1,COL1A2,CXCL12,DENND3,ERCC6L,ETS2,FBN1,FOSL1,GEM,GLI1,GPRC5A,HJURP,IL1RN,IL2RG,KIF2C,LIPG,MMP2,MMP9,MSI1,MYO9B,NAV2,OAS1,OSR2,PTCH1,SOX2,SPHK1,SPRY1,TLR2,TPBG,TRAF1,UGCG	363 (15)
ATF6		transcription regulator		0.577	0.00175	AURKA,BUB1,DNAJB11,FDPS,NUCB2,PDIA4,SLC35B1,SPARC,SRR,TROAP,UBE2C,UNC13B	
EIF4G1		translation regulator		1.588	0.0018	ATRX,BIRC5,BRCA1,BRCA2,CHEK1,CTNND1,GADD45A,RAD51,RFC4,XIAP	

SATB1		transcription regulator		-0.576	0.00201	ADCY3,CEACAM1,DSTYK,DUSP4,EPSTI1,FAM129A,FERMT2,GATA3,GLRX,GNG4,GPT2,HELZ2,HLA-DMB,HSP90AA1,HVCN1,IL18RAP,NR4A2,NUP153,PIK3IP1,PRKCB,PSMD2,PTGS2,SGK1,SIPA1L2,SUN2,UHRF1,WARS	
FOXO3	-1.608	transcription regulator		-1.074	0.00209	APAF1,BCL2L11,BIRC5,BNIP3,CAT,CCNG2,CDC42EP3,CLDN1,EGR2,FBXO32,FOXO1,GADD45A,IER3,MCAM,MMP9,SGK1,TXNIP,VIM,YBX1	473 (17)
KRAS		enzyme		1.455	0.00217	CCND1,CCND3,DUSP4,DUSP6,E2F1,EGFR,ETV5,FTH1,GADD45A,GLI1,IER3,IGF1R,ILK,PDE4B,PLCD1,TNFRSF10A,UPP1	412 (14)
NELFCD		other		1	0.00222	BSG,CCNE2,JUNB,MYBL2,TOP2A	
TMPO		other	Inhibited	-2.236	0.00222	ASPN,COL12A1,COL1A1,COL3A1,MMP15	
ZNF652		other			0.00222	EGFR,TGFB1,TGFB2,TGFBR2,VIM	
AMH		growth factor			0.00222	CXCL2,E2F1,IER3,RBL1,RBL2	
STAT3		transcription regulator		-1.633	0.00245	ACTA2,AKT1,ANGPTL4,BIRC5,BNIP3L,CA9,CCND1,CEACAM1,CHEK1,COL1A1,DDIT3,EGR2,EGR3,FN1,FSCN1,GLIPR1,HAS2,HIST2H2AA3/HIST2H2AA4,HK2,IKBKE,IL1RN,ITGAV,JUNB,KCTD11,LBP,MMP2,MMP9,NDRG1,NFATC2,NR4A2,NUMBL,PDK1,PGK1,PIM1,PSIP1,PTGS2,SGK1,SNAI2,SOX2,STAT1,TAGLN,TIMP1,TNS1,VCAN,VEGFA,VIM	467 (15)
CCNK		kinase	Activated	2.366	0.00246	BRCA1,BRCC3,CBY1,FANCD2,GABPB2,PLTP,POLA2,RFC4,RPA2,TAF12,TIMELESS,UTP14A	
ZBED6	-1.665	other		0.707	0.00246	BOP1,CRIM1,DDIT4,IGF2,KITLG,MB21D2,MYBL1,NASP,SGK1,TTK,UPP1,WWC1	
BRD4		kinase		-1.137	0.0026	ABCC4,ACSL5,ACTA2,AURKB,CCND1,CDC25A,COL1A1,COL1A2,COL4A1,COL5A1,CXCL2,FBN1,FKBP11,FN1,ITGA1,ITGA5,KCNQ5,LOXL1,LOXL2,MAP3K1,PDGFA,PDGFC,PIM1,PLAT,PLAU,POLE2,PTPN22,SLC19A1,SORD,THBS2,TIMP1,XBP1	
LRP6		transmembrane receptor		-0.436	0.00274	ACTA2,CAV1,GATA3,HK2,KRT18,SLC2A3,VIM	

miR-451a (and other miRNAs w/seed AACCGUU)		mature microrna		0	0.00275	ADAMTS5,AKT1,CCND1,FBXO33,MMP2,MMP9	
miR-100-5p (and other miRNAs w/seed ACCCGUA)		mature microrna		-0.405	0.00275	CCND1,CCND3,EGR2,FGFR3,ID1,IGF1R	
IgG		complex		-0.255	0.00301	ASAH1,ATP1B1,ATP1B3,B2M,CD9,CEBPB,CEBPD,CYP27A1,DDIT3,DEGS1,DUSP1,EFNA3,FST,GPRC5A,HDGF,IL1RN,JUND,KRT16,KRT18,PEA15,PIM1,PTGS2,RND3,SERPINB7,SLC2A3,STK24,TNFSF15,TOB1,TUBB2A,UGCG,VEGFA,ZFP36	311 (11)
let-7		microrna		-1.753	0.00305	ACTA2,AURKB,BDNF,BMPR1A,BOP1,CCND1,CDC25A,CEBPD,DUSP6,E2F2,FN1,FOSL1,GAB2,HK2,HMGA1,IGF2BP2,LSM6,MCM2,MRTO4,NFKB1,PTGS2,RBM38,RDH10,SLC25A13,TRIB1,VIM	186 (7)
TFAP2A	1.897	transcription regulator	Inhibited	-2.213	0.00351	ALCAM,BIRC5,CXCL2,DCLK1,F2R,GEM,ITGBL1,KLHDC3,MMP2,PLAU,PPARD,RAB27B,SESN1,SESN3,TPM3,VEGFA,WISP2	
ETV5	1.615	transcription regulator		-1	0.00357	ALCAM,CCND1,CLDN1,FN1,ITGA5,KRT16,KRT80,MMP2,PKP2,PKP3,SNAI2,TJP1,TJP2	
NCOA1		transcription regulator		-0.132	0.00364	AGRN,C3,CCND1,DGKQ,FRAT2,GPBP1,HAS2,HIC1,HIRA,NCAPH2,NFKBIZ,PGS1,SMG6,TLE1	
GNE		kinase	Inhibited	-2	0.00364	ASNS,CHAC1,DDIT3,TRIB3	
NFYC	1.642	transcription regulator			0.00364	CDCA8,COL5A3,FTH1,SGK1	
A2M		transporter		1.929	0.00394	ATF4,BAD,CCND1,ERN1,FOXO1,MAP3K5,NFKB1,SREBF2,XBP1,XIAP	

IL5		cytokine		1.479	0.00456	CCND3,CEACAM1,CSF2RB,DUSP6,EGR2,EGR3,GCLM,HSPH1,IER3,IL3RA,PIM1,QSOX1,RBM3,UPP1,XBP1,ZNF25	
GATA6	-3.046	transcription regulator		0.793	0.00456	ABCB1,CDCA8,COL4A1,COL4A2,DAB2,DSCC1,ESPL1,GATA6,HES1,NUSAP1,PDGFRA,SOX2,SPARC,STAR,TGFB1,TGFB2	
HDAC2		transcription regulator		1	0.00461	BRCA1,COL1A2,DPT,FDXR,FOXM1,IGF1,ITGB8,MMP9,PREX1,PTGS2,SMN1/SMN2,TP63	443 (13)
miR-34a-5p (and other miRNAs w/seed GGCAGUG)		mature microrna	Inhibited	-2.857	0.00461	BIRC5,CCND1,CDK1,DHFR,E2F1,E2F2,E2F3,IKBIP,KLF4,MCM10,MCM3,SOX2	
SRSF3		other		1.304	0.00468	CCND1,CCND3,E2F1,E2F7,FOXM1,HIPK2,VEGFA	
SP4		transcription regulator		-0.115	0.00468	BIRC5,CCND1,EGFR,FLT1,IGF1R,NFKB1,VEGFA	
AKT1	1.71	kinase		1.437	0.00473	ABCB1,AKT1,BCL2L11,CCND1,FOSB,FOXO1,GCLC,GCLM,GSR,JUND,SLC4A7,STUB1,UNC5B	230 (12)
SUPT16H		transcription regulator			0.00494	AP1G1,HIST1H1C,HIST1H2AC,HIST1H2BA,KRT8,LOXL2,MSN,PDGFA,PLAU	
TEAD1	-1.505	transcription regulator			0.00495	LATS2,MSLN,TP63	
SRSF5		other			0.00495	AZIN1,NR3C1,VEGFA	
PRKDC		kinase		1.455	0.0051	ABCB1,AHR,CCND1,EGR2,NR1D1	187 (7)
NELFE		other		0.528	0.0051	BSG,CCNE2,JUNB,MYBL2,TOP2A	214 (3)
SMAD1	1.742	transcription regulator		-0.179	0.0051	ACTA2,COL1A1,COL1A2,CXCL2,PDGFA	
SCUBE3	-2.209	other		-0.179	0.0051	MMP2,MMP9,SNAI2,TGFB1,VIM	27 (3)
I kappa b kinase		complex	Inhibited	-2.438	0.00517	BRCA1,BRCA2,FANCA,FANCC,FANCD2,RAD51	

P38 MAPK		group		0.433	0.00521	CCNF,CCNG2,CEBPB,COL3A1,CXCL12,CXCL2,CYP4F3,EGR2,FDXR,FLNA,FN1,FST,GCLC,GOLGA2,IER3,INHBA,ITGAV,ITGB8,LAMA3,MMP2,MMP9,NFKB1,PLA2G4A,PLA2G7,PTGS2,RASA1,RBP1,RND3,RRAD,STAT1,TGFA,TGFB1,TIMP1,TJP2,TLR2,TOP2A,TRADD,TRIB1,VEGFA	532 (17)
CD44		other		-1.367	0.00523	ABCA5,ABCB1,ABCG2,ACTA2,BIRC5,CCND1,COMMD3,DNMT1,FN1,HDGF,ITGAV,LAMC1,MBNL3,MMP14,MMP9,PARVB,PLAU,SOX2,VIM,WNT5A,XIAP	276 (12)
IL13		cytokine		0.751	0.00525	ACADVL,ADA,AMPD2,AVPI1,BDNF,C3,CASP1,CD14,CHCHD7,CHST2,COL1A2,DHCR24,DUSP10,ENPP2,FAM162A,FLOT1,GAS7,GPNMB,HPSE,IARS,IL13RA1,IL1R1,IL1RN,IL3RA,KITLG,LTA4H,MAF,MAOA,MCUR1,MSMO1,NCAPH,PAPSS1,PDGFC,PDLIM2,PHLDA1,QSOX1,RFTN1,SEPT11,SERPINF1,SLA,SLC16A6,ST3GAL6,SWAP70,TNS1,WNT5A	
RARA		ligand-dependent nuclear receptor	Activated	3.415	0.00545	BIRC5,CA12,CCNA2,CCND1,CCNT2,CCPG1,CD14,CD38,CD9,CDK1,CENPA,CENPM,CENPN,CENPU,CP,CTNND1,DNAJC21,EIF5A2,ENY2,FLCN,HELLS,INIP,KIF23,LGALS3BP,MAD2L1,MAOB,NAV2,NCAPG,NCAPH,NEDD9,PBK,PHLDB1,PLK4,RAP1A,RGS3,SESN1,SETD5,SHANK2,SIPA1L1,SLC7A11,SMC4,SP110,SPP1,THBD,TNFRSF10A,UBL3,XBP1,ZNF367	
Cdk		group	Activated	2.53	0.00558	CXCL2,DDIT4,DUSP1,GADD45A,GADD45G,ING1,NEDD9,PHLDA1,SIAH1,TXNIP	
HDAC6	2.341	transcription regulator		-1.584	0.00558	BIRC5,COL1A1,IGF1,JUNB,LTBP2,NR3C1,PLAU,TGFB1I1,TGFBI,VIM	
miR-29b-3p (and other miRNAs w/seed AGCACCA)		mature microrna		0.389	0.00596	COL1A2,COL5A2,FRAT2,GAS7,LAMC1,LOXL2,MYBL2,PPIC,PURA,SPARC,TUBB2A	

HNRNPA 2B1		other			0.0061	ABCB1,ACSL5,ANXA7,ARSJ,ATRX,CA12,CEMIP,CLDN1,CP,CRIP1,DCLK1,DNER,DSE,EYA4,FHL1,FN1,FSCN1,GJA1,HIPK2,IDS,MFHAS1,MSRB3,NR2F1,OAS1,P4HA1,PDGFC,PLCB4,PLOD2,SEMA3B,SFI1,SYT1,TGFB2,TLE1,UTRN,VCAN,WNT4	
MMP2	-1.513	peptidase	Activated	2.06	0.00614	CCND1,CSF2RB,IL1RN,IL2RG,IL4R,IL6ST,TGFB1,TJP1	266 (7)
MYB		transcription regulator		0.271	0.00614	AXIN2,BIRC5,CCNB1,CCND1,CXCL12,FN1,GATA3,KLF4,PCNA,POLA1,SNAI2,VIM	
ATM		kinase		-0.353	0.00614	ALKBH8,CCND1,CDKN2D,CFLAR,DUSP1,FN1,GADD45A,GADD45G,IGF1R,MICB,PCNA,RRM2	476 (17)
CDH1		other		0.989	0.00617	BIRC5,CCL17,EGFR,MMP14,MMP19,MMP9,RALA,SOX9,TGFB1,VEGFA,VIM,XIAP,XPC	341 (15)
MAX		transcription regulator		0	0.00617	APEX1,CBX5,CCNG2,CDC25A,DDIT3,DHFR,E2F1,FBXO32,GADD45A,RBBP8,TMEM126A,TXNIP,UBE2C	
Hdac		group		0.498	0.00636	APAF1,BIRC5,CCND1,CCNG2,DDIT3,EGFR,EGR3,FBXO32,KCNH2,KLF9,NDRG4,PIK3R5,PREX1,RGS10,SP1,TGFBR2,TP63,TXNIP	285 (8)
Growth hormone		group		-0.466	0.00636	ANGPTL4,BMP2,CAT,CCND1,CITED2,FKBP5,FZD5,FZD7,GCLC,IER3,NEDD9,NR1D1,PDK4,PIK3C2B,PLK2,SGK1,TGFB3,TXNIP	
ERG		transcription regulator		-1.659	0.00687	ARHGAP22,ARHGAP28,ARHGEF2,AXIN2,CAMK1D,CCNA2,CDKN2D,CLIP1,DBN1,FLNB,FLNC,FLT1,FMNL3,HDAC6,LAMA5,MAGI1,MYO1D,NPHP1,ORC6,PLAU,PTPN22,RALGPS2,RASA2,RASSF1,RCAN2,RGS3,RHOBTB1,RHOJ,RSU1,SLIT2,SREBF2,SVIL,TMEM263,TRIOBP,UTRN,VIM	
TRIB3	2.55	kinase	Inhibited	-2.596	0.00749	ASNS,DDIT3,DDIT4,MTHFD2,PSAT1,PSPH,TRIB3	
VHL		transcription regulator		1.757	0.0077	CA12,FTH1,H2AFX,LMNA,MAD2L1,MMP14,NEK8,TFRC,TGFA,VIM	
ELK1	1.902	transcription regulator		1.354	0.0077	BMP2,FOSL1,ITGAV,MTHFD2,NUPR1,PRKCA,SLC7A11,SPP1,TIPARP,TPD52L1	
Gsk3		group		1.594	0.00798	ATF4,CCND1,CFLAR,DDIT3,EGFR,FOSB,HMGCR,IGF1R,NR4A2,SREBF2,XIAP	298 (14)
mir-34		microrna		-1.992	0.00798	ACTR1A,BIRC5,CCND1,CCNE2,CDC25A,CPLX2,E2F3,EFNB1,KCNH2,MDM4,PIP5K1A	

TRAP1		enzyme		-1.732	0.00804	EARS2,GPT2,HIBADH,ISCU,MARS,MRPL18,MTHFD2,ODF2,PTGS2,SARS,SMDT1,TXN2	
Interferon alpha		group		-1.089	0.00819	A4GALT,ACP2,ADAM19,BAK1,CASP1,CCND3,CDC25A,CGAS,CSF2RB,DDIT4,DRAP1,DUSP6,E2F1,ENPP2,EPSTI1,GATA3,GLS,GPR180,HELZ2,IFIH1,IKBKE,IL15,IL18RAP,ISG20,MORC2,MYLIP,NABP1,NFIL3,OAS1,PAPD7,PHF11,PLSCR1,PRKACA,RABGAP1L,SAT1,SERPINB1,SERPINB9,SLC2A3,SLFN5,SP110,SPRY1,SPRY2,SPRY4,SREBF2,STAT1,TGFB1,TICAM1,TLR2,TLR3,TMEM140,TRIB2,TRIOBP,TTC28,WDFY1	
EFNA2	-1.94	kinase		0	0.00875	BACH2,DUSP4,DUSP6,ETV5,FOSL1,FOXQ1,KRT16,KRT18,NFIL3,PKP1,PLAT,SLC20A1,SOX9,UBE2C,UBE2S,WWP1	
NR6A1		ligand-dependent nuclear receptor		-0.816	0.00886	BMP1,CCND3,GATA3,GLS,LMNA,VIM	
PTTG1		transcription regulator			0.00886	CCNA2,CCNB1,CCND1,SOX2,VEGFA,VIM	303 (7)
Histone h3		group			0.0089	B2M,BCL2L11,CA9,CAV1,CCND1,CD9,CDK14,CEACAM1,CFLAR,DAB2IP,EFNA1,ENO3,FAM49A,FKBP5,FMR1,FOXM1,GABPB2,HES1,HOXA11,HOXB6,HPSE,ID1,IGF1R,MAGED2,MTUS1,MYBL2,NDUFAF2,NDUFAF7,NDUFS2,NDUFS4,PALLD,PCBD2,PCNA,POLR2A,PROS1,PSMA2,PTCD2,PTGS2,RAB27B,RASSF1,RBL1,RGS10,RPL12,SCD,SHANK3,SIAH1,SMN1/SMN2,SPHK1,STAR,TLR2,TOP2A,TPM3,VKORC1,WASF1,XBP1,ZNF423	394 (11)
NEUROG1		transcription regulator		-0.471	0.00931	AMIGO2,C1S,C3,CEMIP,CFH,COL3A1,FN1,HAS2,INHBA,LRIG1,MARC2,MMP9,P4HA2,PLK2,PPIC,PXDN,SERPINF1,TACR1	
EIF3A	1.535	other		0.132	0.00945	RPA2,RPA3,XPA,XPC	
HIF1AN		enzyme		0	0.00945	CA9,EGLN3,HK2,SOX9	
PIWIL4		other		-1	0.00945	FGFR2,TGFB1,TGFB3,TGFBR2	
POSTN	-25.428	other		-1.131	0.00945	EGFR,MMP9,TGFB1,VIM	
NAB2		transcription regulator			0.00945	BCAR1,FLT1,PLAU,RRAD	

ELAVL1		other	Activated	2.045	0.00953	CDC6,CENPA,CENPE,DGAT1,DUSP1,FLNA,GATA3,KIF11,KIF18A,MYBL2,NCAPG,OSBPL2,PBK,PLCG2,PRC1,PTGS2,SAT1,TAF9,TIMM17A,TOP2A,TP63,TRIOBP	
ESR2		ligand-dependent nuclear receptor		-0.881	0.0097	BIRC5,C3,CAPN2,CCNA2,CCND1,CXCL12,EGFR,FOXM1,GSTP1,HOXC6,IGF1R,LTBP1,TGFA,VEGFA	377 (17)
XBP1	-1.831	transcription regulator		-1.718	0.0097	APBB2,CAT,DDIT3,DNAJB11,DNAJC3,ERLEC1,KLF4,NUCB2,PDIA4,S100A6,SMC3,SPARC,TFDP1,XBP1	
PAX8		transcription regulator	Activated	2.217	0.00987	CCNA2,CDC6,DHFR,E2F1,MCM3	
TPM3	1.652	other		1.342	0.00987	IGF1,ITGA1,ITGA3,MMP2,VEGFA	
NELFA		other		1	0.00987	BSG,CCNE2,JUNB,MYBL2,TOP2A	
MCAM	-62.696	other		-0.243	0.00987	FN1,ID1,MMP2,SNAI2,VIM	
CSNK2A1		kinase		-0.447	0.00987	CCND1,DUSP6,ITGA3,TP63,ZFP36	
FLT3		kinase			0.00987	CCND3,CDC25A,CEBPB,LIG3,PIM1	
LEP		growth factor		-0.318	0.0101	ACAT1,APAF1,BAD,BAK1,CASP7,CCNA2,CCND1,DFFB,E2F3,FAAH,GADD45A,IL1RN,PCNA	
BRCA1	1.615	transcription regulator		0.806	0.0103	BAK1,BIRC5,BRCA1,CCNB1,CCND1,DDIT3,E2F3,EGFR,ENSA,FEN1,FOXO1,FOXO3,GADD45A,H2AFX,PCBP2,PCNA,RAD51,UBE2I,VCAN	
SPP1	23.369	cytokine		0.043	0.0103	AURKA,BCL2L11,CDC20,CXCL12,FN1,HAS2,ILK,KRT18,MMP2,MMP9,NDUFS4,NDUFS8,PLAU,SNAI2,SOX9,SSB,TGFB1,TXNRD2,VIM	
S100A6	1.956	transporter		1.897	0.0104	AURKA,CENPA,CXCL2,DEPDC1,DHRS9,INHBA,NCAPH,PBK,TYMS,YBX3	
SIN3A		transcription regulator		0.816	0.0104	CCNG2,COL1A1,COL1A2,DDIT3,FBXO32,GSTP1,MAD1L1,MMP9,PTGS2,TXNIP	
HMGA1	3.518	transcription regulator		0.526	0.0104	CAV1,CCND1,ELK1,HMGCR,IDI1,KITLG,MMP9,PSEN2,PTGS2,XPA	
SMARCA2		transcription regulator		0	0.0105	CP,DHFR,E2F1,FKBP5,RAB27A,RBL1,RRM2,TAGLN,TBX2,TYMS,TYR	

Jnk		group		0.119	0.0108	ACTA2,APP,ASAH1,BCL2L11,BIRC5,BRCA1,CCND1,CFLAR,CXCL12,DUSP1,FOSB,GJA1,JUND,MAP3K11,MMP14,MMP2,MMP9,PTGS2,TGFB1,TGFBR2,TIMP1,VCAN,XIAP,ZFP36	
CD24		other		0.943	0.0109	CAV1,CHAC1,DEPDC1,DEPDC1B,ECM1,EDEM3,EMP3,FRYL,ISG20,KIF18A,KNTC1,MBNL1,MCAM,MTUS1,PLAU,PTP4A1,RASA1,SLC30A1,SLC4A4,SMC4,SPHK1,TFPI,TMEM135,TOP2A,TRIM8,UPF2,VPS13C	
EGFR	-2.139	kinase		0.862	0.0113	ABCG2,ACTA2,AKT1,BCL2L11,CAV1,CCND1,CEBPB,DDIT3,E2F1,E2F2,E2F3,EGFR,HK2,IGF1,ITGA6,MYBL2,PLAU,POSTN,PSMB3,PSMB7,PTGS2,SLC7A11,SOX9,SPHK1,TPST1,VEGFA,VIM,XIAP	
FGF7		growth factor	Activated	2.028	0.0114	ADSS,CEBPD,FGFR2,HPRT1,IL7,SCD,STAT1	
MYOC		other			0.0122	CARD10,CDH11,DAB2,DDIT3,DDIT4,DPP7,FBN1,FN1,FSCN1,HOXA7,MAFK,PRKCB,PTGER4,PXDN,RAB27B,SLC2A3,SLC7A11,UPP1,VAPA	
IL6		cytokine	Activated	2.021	0.0126	ABCG2,BAK1,BIRC5,C3,CCND1,CEBPD,E2F1,E2F2,ENO2,ETS2,GADD45G,IL1RN,IL6R,IL6ST,JUNB,LBP,LYZ,MMP2,NEU3,NFKB1,PDGFA,PLAU,PTGS2,RASA1,RASSF1,SGK1,TGFB1,TIMP1,TRAF3,VEGFA,XIAP,XRCC5	
STAT5a/b		group		0	0.0127	AHR,CCND1,EPHA4,IDI1,MAF,PDE4B,PIM1,RBP1,RFTN1,SLC2A3,STIP1,UGP2,XIAP	
NEDD9	2.026	other		-1.554	0.0132	APP,BMP2,BNIP3,CA9,DDIT4,MKNK2,MMP14,MMP2,MMP9,PGK1,PLOD2,TXNIP	
BCL6		transcription regulator		0.444	0.0133	ALCAM,BLNK,CCND1,CHEK1,COL1A1,DUSP6,FCGRT,FMOD,FTH1,FUT7,GADD45A,ITGA3,MITF,NFKB1,SDC1,SOX9,SYK,XBP1	
UHRF2		enzyme		0.333	0.0136	ADIPOR2,AKT1,BCL2L11,CBX7,FOXO1,FOXO3,PPP1R13B,TRAF3,ZNF672	
SSRP1		other			0.0136	AP1G1,HIST1H1C,HIST1H2AC,HIST1H2BA,KRT8,LOXL2,MSN,PDGFA,PLAU	
PRKCE		kinase		1.008	0.0136	ABCB1,AMIGO2,BIRC5,CAV1,CCND1,MMP9,PDGFA,PIM1,PTGS2,VEGFA,XIAP	
BRAF		kinase		0.555	0.0136	BCL2L11,CCND1,CDK1,DNMT1,DUSP4,MMP9,PCNA,RND3,TYR,XBP1,XIAP	

SBDS	-1.53	other			0.0137	CHAC1,COL27A1,DDIT3,DNAJB1,EPDR1,FBXL16,FBXO32,HOXB6,ID1,IFI16,IGFBP7,IL4R,MAFF,MAOA,OPN3,P2RX5,PCDH7,PTBP3,RGS4,SNAI2,SPP1,TNC,TRIB3	
EZR		other	Inhibited	-2.449	0.0141	ATF4,DDIT3,DDIT4,MMP9,PTGS2,TRIB3	
TGFBR2	-2.25	kinase		-0.97	0.0144	ACTA2,ANGPTL4,ATF4,BDNF,CASP1,GATM,ING1,MAN2B2,MAP3K11,MMP2,SLC12A4,SLC2A3,TGFB1,TGFBR2,TIMP1,TXNIP,UBE3A,VCL,WDFY3	
SREBF1		transcription regulator		0.394	0.0149	ACADS,ARF4,CD14,CHMP2A,CSRP1,CYCS,DARS,DGKQ,FBXO32,FDPS,FOXO1,GLB1,GPNMB,GSR,IFI30,NUPR1,OAT,P4HA2,PMPCA,RBP1,RPS24,S100A13,SCD,SLC20A1,SLC22A4,SLC22A5,SORBS3,TTN,VGF	
IL15	2.87	cytokine		1.397	0.016	AKT1,BPGM,CAMK2G,CD38,ENO2,ENO3,GPC1,HK2,HLX,IGF1R,IL18RAP,IL2RG,NFKB1,PDK1,PGK1,PIK3R1,PIK3R2,PIK3R3,PLCG2,RPIA,SLC16A1,SLC16A3,TALDO1,TFRC,TGFBR2,VEGFA,ZFP36	
USF1		transcription regulator		-0.155	0.0166	APLN,B2M,BRCA2,CCNG2,CEACAM1,FBXO32,GATA3,P4HA1,SLC19A1,SPP1,TXNIP,TYR	
let-7a-5p (and other miRNAs w/seed GAGGUAG)		mature microrna		0.875	0.017	ACTA2,BIRC5,CCND1,COL1A2,VIM	
SRSF1		other		0.562	0.017	BIRC5,FN1,LMNA,SMN1/SMN2,VEGFA	
mir-203		microrna		-1.387	0.017	BIRC5,EZH2,HES1,RNF2,TP63	
DEF6		other		-1.412	0.017	FN1,MMP2,MMP9,SNAI2,VIM	
DPY30		enzyme			0.017	CCNE2,E2F1,E2F2,ID1,ID3	
NOTCH1		transcription regulator		0.366	0.0171	ACTA2,CEBPB,CHST1,EFNB2,EGFR,ENO2,HES1,HEY1,IGF1R,IL18,PTPRK,RHOU,RND3,TGFB1,TGFB2,TGFB3,TP63	
GLIPR2		other			0.0173	ACTA2,EGFR,VIM	
PHF8		enzyme			0.0173	CDC25A,E2F1,RBL1	
HCAR1		g-protein coupled receptor			0.0173	BSG,SLC16A1,SLC16A3	

CDK1	1.564	kinase			0.0173	CDK1,H2AFX,XBP1	
BMP15		growth factor			0.0173	HAS2,PTGS2,TNFAIP6	
RPSA		translation regulator			0.0173	DUSP1,ITGA6,MMP2	
miR-515-5p (and other miRNAs w/seed UCUCCAA)		mature microrna			0.0173	FGFR2,PIK3C2B,SPHK1	
miR-192-5p (and other miRNAs w/seed UGACCUA)		mature microrna			0.0173	BIRC5,IGF1,IGF1R	
mir-320		microrna			0.0173	AQP1,BIRC5,TFRC	
GDF9		growth factor			0.0173	HAS2,PTGS2,TNFAIP6	
MTTP	-1.815	transporter			0.0173	GOT1,GPT,MTTP	
NKX2-3		transcription regulator			0.0173	ACTA2,AOC3,SHOX2	
SRSF6		other			0.0173	BCL2L11,LMNA,VEGFA	
MFAP2		other			0.0173	CDC42EP3,PTCH1,VCAN	
HSPA1A/HSPA1B		enzyme			0.0173	CDK1,KCNH2,PSIP1	
ACO1		enzyme			0.0173	APP,FTH1,TFRC	
SNAI2	1.661	transcription regulator		0.427	0.0177	AXL,BRCA2,BSG,CCND1,ITGA3,SNAI2,TP63,VIM	
HRAS		enzyme			0.018	BIRC5,EGFR,ELN,FOXO1,IGF2,ILK,ITGA5,ITGA6,PRKCA,PRKCB,SESN1,SESN3,SPRY2,TNFRSF10A	
CTLA4		transmembrane receptor	Inhibited	-2.333	0.018	CCNA2,IL3RA,KPNA2,MKI67,PADI2,PRKD3,RAB30,TOP2A,TPX2	

TREM1		transmembrane receptor	Activated	2.245	0.0185	ASNS,ATP1B1,CCL17,CDK1,CENPU,CFB,CKS2,CXCL2,DTNBP1,DUSP4,E2F7,EGR2,EGR3,ETS2,FERMT2,FNDC3A,FOSL1,GCLM,GEM,GPRC5A,HBEGF,HES1,HSD3B7,IL15RA,INHBA,MAD1L1,MAFF,MMP19,NR4A2,PHLDA1,PTGS2,RHOU,RRAD,SPP1,SPRY2,TBC1D7,THBD,TNFSF15,WNT5A,YRDC	
EP300		transcription regulator	Activated	2.2	0.0189	AXIN2,BIRC5,CA12,CCND1,CCNG2,CDC25A,CFLAR,E2F1,GATA3,ID3,IGF1,ITGB2,MKI67,PLA2G4A,PLA2G7,PTGS2,SOX2,SPHK1,TLR2,UBE2C,XBP1	
EDN1		cytokine		1.954	0.0191	FST,HBEGF,INHBA,PTGS2	
PRKN		enzyme		0.328	0.0191	ACTA2,EGFR,MAOA,MAOB	
DYRK1B	-1.896	kinase		0.064	0.0191	CCND1,CCND3,CP,SOD3	
Calcineurin protein(s)		complex		0	0.0191	AQP5,BCL2L11,NFATC2,PTGS2	
ZNF100		other		0	0.0191	DDAH1,NEDD9,POSTN,TGFB3	
ZNF85		transcription regulator		0	0.0191	DDAH1,NEDD9,POSTN,TGFB3	
ZNF254		other		0	0.0191	DDAH1,NEDD9,POSTN,TGFB3	
RASSF8		other		0	0.0191	DDAH1,NEDD9,POSTN,TGFB3	
ZNF431		other		0	0.0191	DDAH1,NEDD9,POSTN,TGFB3	
IGF2R		transmembrane receptor		0	0.0191	DDAH1,NEDD9,POSTN,TGFB3	
ZNF665		other		0	0.0191	DDAH1,NEDD9,POSTN,TGFB3	
ZNF528		other		0	0.0191	DDAH1,NEDD9,POSTN,TGFB3	
ZNF43		other		0	0.0191	DDAH1,NEDD9,POSTN,TGFB3	
ZNF429		other		0	0.0191	DDAH1,NEDD9,POSTN,TGFB3	
ZNF91		transcription regulator		0	0.0191	DDAH1,NEDD9,POSTN,TGFB3	
ZNF708		other		0	0.0191	DDAH1,NEDD9,POSTN,TGFB3	
mir-214		microrna		-1.979	0.0191	ALCAM,BIRC5,PTGS2,TFAP2A	
MIA		other		-1.98	0.0191	FN1,MMP14,PLAT,SPARC	
BTRC		enzyme			0.0191	AXIN2,CCND1,DEPTOR,PHLPP1	

POU5F1		transcription regulator		0.808	0.0196	AKT1,APAF1,BAD,BAG3,BAK1,BCL2L11,BFAR,BNIP3,BNIP3L,CASP1,CASP7,CRADD,DLX1,DLX4,GADD45A,GATA6,HOPX,HOXC4,IER5L,IGF1R,KRT18,LHX2,MMP2,MMP9,NAIP,NCAM1,SNAI2,SOX2,TNFRSF10A,TNFRSF9,TRAF3,ZFHX3	
FGF2		growth factor		1.119	0.0206	BIRC5,EFNB2,FLT1,MMP9,PLAU,SDC1,ST3GAL3,ST3GAL4,TFPI,TGFBR3,VEGFA,VIM	
TGFA	-3.373	growth factor		1.172	0.0212	AKT1,CEBPB,EGFR,PTGS2,VEGFA,VIM	
AZGP1		transporter		0.105	0.0212	CCND1,CEBPB,GYS1,HK2,ITGA5,VIM	
MUC4		other		-0.875	0.0212	CCND1,EGFR,KRT18,MMP9,TWIST2,VIM	
UPF1		enzyme		-0.896	0.0212	DNAJB11,SGK1,SMG5,SMG6,UPF2,UPF3B	
miR-145-5p (and other miRNAs w/seed UCCAGUU)		mature microrna		0.01	0.0228	CES1,DTD1,FSCN1,KLF4,SOX2,SWAP70,TAGLN,TPM3,UNG,VASN	
TWIST2	1.947	transcription regulator		-0.479	0.0232	CCND1,FN1,POSTN,SNAI2,SOX2,TGFB1,VIM	
NPPB		other		-1.964	0.0232	EBP,FDXR,HMGCR,HMGCS1,IDI1,MSMO1,STAR	
ECSIT		transcription regulator	Activated	3	0.0235	CFLAR,IER3,IL1RN,JUNB,NFKB1,NR4A2,PIM1,PTGS2,TRAF1	
PPARD	1.529	ligand-dependent nuclear receptor		1.185	0.0235	ANGPTL4,BIRC5,CEBPB,FLT1,ILK,PDK4,PPARD,PTGS2,VEGFA	
SND1		enzyme			0.0235	ALDH3A1,ANGPTL4,CDC6,DHFR,E2F1,ID1,LTBP1,NR2F1,PTGS2	
CTGF		growth factor		-0.309	0.0238	ADAMTS5,EGLN3,FN1,MMP2,MMP9,SOX2,TIMP1,TIMP3	
BSG	-1.685	transporter		-1.143	0.0238	BCL2L11,BSG,CCND1,CIP2A,CXCL12,IL18,MMP2,MMP9	

NFKB1	1.517	transcription regulator		0.631	0.024	B2M,COL1A1,CXCL2,DUSP1,EGFR,FANCD2,FCGRT,FOSB,FSCN1,HAS2,HLA-DMB,IL1RN,MMP9,NFKB1,NR4A2,ORAI1,PLAU,PLK3,POLB,PTGS2,PTPN6,TGFB1,VEGFA,XIAP	
COL18A1		other		-1.809	0.025	CCND1,DDIT4,EFNA1,EFNB2,EGFR,F2R,FN1,ID1,ID3,ITGB2,JUNB,MMP2,NRP1,PLAU,PTGS2,STAT1,VEGFA,VWF	
DNMT3B		enzyme		-0.221	0.0263	CBX5,CDK1,DNAJB1,FBXL16,FOXF2,GLT8D2,LACC1,LXN,NAP1L2,PLP2,PRKCB,PRRC2C,PUSL1,RASSF1,RCC1,RECK,SERPINB9,SLC30A1,STAT1	
CNOT7		transcription regulator			0.0286	B2M,CLDN1,CMPK2,LGALS3BP,OAS1,PLSCR1,SLC14A1,SLC7A11,SP110,STAT1	
Fgf		group			0.0291	SPRY2,SPRY4	
MCPH1		other			0.0291	BRCA1,CHEK1	
CERS5		transcription regulator			0.0291	CERS2,DDIT3	
THRAP3		transcription regulator			0.0291	CCND1,HAS2	
BTBD7		other			0.0291	MMP2,MMP9	
ASCL1		transcription regulator			0.0291	CDK5,CDK5R1	
SELENOH		other			0.0291	GLB1,H2AFX	
DNAJC3	-2.753	other			0.0291	ATF4,DDIT3	
DAPK3		kinase			0.0291	BIRC5,CCND1	
NODAL		growth factor			0.0291	CCNG2,FOXO3	
TAC4		other			0.0291	MMP14,MMP2	
mir-192		microrna			0.0291	IGF1,IGF1R	
miR-133a-3p (and other miRNAs w/seed UUGGUCC)		mature microrna			0.0291	FSCN1,STK3	

miR-331-3p (miRNAs w/seed CCCCUGG)		mature microrna			0.0291	CDCA5,KIF23	
miR-224-5p (miRNAs w/seed AAGUCAC)		mature microrna			0.0291	MMP9,PAK4	
NUBP1	1.784	other			0.0291	FTH1,TFRC	
NCK1	-1.895	kinase			0.0291	ATF4,DDIT3	
PTH1R		g-protein coupled receptor			0.0291	CDC25B,CDK1	
RHOH		enzyme			0.0291	IL3RA,ITGB2	
EIF4E2		translation regulator			0.0291	EGFR,PDGFRA	
FGF10		growth factor			0.0291	MMP14,TGFB1	
FOLR1		transporter			0.0291	CAV1,TYMS	
MUC16		other			0.0291	CCNB1,CFLAR	
PTGS1		enzyme			0.0291	MMP2,MMP9	
RBP3		transporter			0.0291	ATF4,DDIT3	
PTP4A2	1.703	phosphatase			0.0291	BCAR1,VCL	
SRY		transcription regulator			0.0291	MAOA,SOX9	
SENP1	2.463	peptidase			0.0291	MMP2,MMP9	
ATP7A	-2.751	transporter			0.0291	APP,PLA2G4A	
SERPINB5		other			0.0291	KRT18,VEGFA	
CDC20	2.062	other			0.0291	CFLAR,UBE2C	
CDC27		other			0.0291	CCND1,UBE2C	
BARD1	1.841	transcription regulator			0.0291	AURKB,BRCA1	

TXNIP	-1.688	other			0.0291	CCNA2,DDIT4	
PDE4D		enzyme			0.0291	BCL2L11,MITF	
SNRNP70		other			0.0291	APP,MDM4	
FTL		enzyme			0.0291	FTH1,TFRC	
KLF6		transcription regulator		-0.103	0.0301	BAK1,BCL2L11,CXCL2,MKI67,MMP9,MSLN,PCNA,TGFB1,VEGFA	
TAL1		transcription regulator	Activated	3.045	0.0303	ADCY3,BTBD3,BUB1,C3,CCNB1,CCNG2,CENPU,CHST11,CIB2,CMTR2,CWC27,DSCC1,EEF1E1,FBN1,GATA3,GDF5,GINS1,GSDMD,HELLS,HIVEP3,KIF20A,LRR1,MAP2,MAP3K1,MCM2,MCM4,MCTP1,MELK,MPND,MSLN,NCAPG,NFKBIZ,NOS1,NTNG2,PPP3CA,PTGER4,PTK2B,SLC2A3,SPRY2,SYK,TOB1,TRAF3,YARS,ZFYVE9	
PPP1R13L		transcription regulator		0.928	0.0303	CLDN1,GJA1,ITGA3,ITGAV,PKP1,TP63	
DNAJB6		transcription regulator		0.294	0.0303	KRT18,SNAI2,SPARC,SPP1,VGF,VIM	
mir-17		microrna		-0.564	0.0303	AKT1,APP,BCL2L11,CAPRIN2,E2F1,H2AFX	
PTAFR		g-protein coupled receptor		-0.673	0.0303	BIRC5,EGFR,MMP2,MMP9,PTGS2,VIM	
SMARCD3		transcription regulator		-0.816	0.0303	ALCAM,COL1A1,FMOD,ITGA3,MITF,SOX9	
TBXT		transcription regulator		-0.816	0.0303	ALCAM,COL1A1,FMOD,ITGA3,MITF,SOX9	
STUB1	1.644	enzyme	Inhibited	-2.449	0.0303	CCNA2,CDK1,PCNA,RAD51,RBL2,VEGFA	
NOTCH3		transcription regulator		1.89	0.0314	BIRC5,CFLAR,HES1,HEY1,ID1,MMP9,NRARP	
miR-486-5p (and other miRNAs w/seed CCUGUAC)		mature microrna		0.431	0.0314	CREBL2,FOXO1,RFFL,SLC4A8,TOB1,TWF1,WDFY3	

ITGAV	-3.315	transmembrane receptor		-0.564	0.0314	CDK1,COL1A1,COL1A2,HAS2,ITGAV,MMP2,TGFB1	
IRF2		transcription regulator			0.0314	B2M,CCND1,CEACAM1,E2F3,ERAP1,IL7,PSME1	
KIT		transmembrane receptor			0.0314	BCL2L11,BIRC5,MITF,PSTPIP2,PTPN6,SH3BP2,STAT1	
CSF2		cytokine		1.099	0.033	CCND3,CD38,CEACAM1,CSF2RB,DUSP6,EGR2,EGR3,FLT1,GCLM,HSPH1,IER3,IL1R1,IL3RA,PIM1,QSOX1,RBM3,TGFB1,TLR2,TLR5,UPP1,XBP1,ZNF25	
Igm		complex			0.033	BAK1,BCL2L11,BIRC5,CCNA2,CFLAR,CYCS,NFKB1,POLI,TRAF1,TRAF5,UGCG	
AIF1		other		-1.154	0.0331	ACTA2,COL1A1,COL3A1,TGFB1	
TFDP1	1.561	transcription regulator			0.0331	CCND1,CDC6,CDK1,RBL1	
MZF1		transcription regulator			0.0331	AXL,NOS1,PRKCA,TGFB1	
HGF		growth factor		0.037	0.0346	AKT1,ANK3,CA9,CCNG2,DDX21,EFNB2,FEN1,HLX,IGF1,LSM1,MMP2,MMP9,NEK4,ORC2,PDGFA,PGK1,PLA2R1,PLAU,PTGS2,SF3B4,SLC9A3R1,TAGLN2,TNFAIP2,TOPBP1,VEGFA	
CXCL8		cytokine		0.995	0.0367	ABCG2,CD74,CFLAR,COL12A1,IFRD1,ITGA5,ITGB2,MMP2,MMP9,PTGS2,SNAI2,SOX2	
LGALS3		other		0	0.0367	CCND1,COL3A1,COL4A1,COL5A2,CTSV,DUSP6,ENPP2,ITGA6,ITGAV,KRT18,NFATC2,PLEK2	
VEGFA	1.662	growth factor		-0.634	0.0367	ANGPT1,EFNB2,FLT1,HES1,ITGA1,ITGAV,MMP2,MMP9,NRP1,PLAU,PRKCA,VEGFA	
Integrin		complex			0.0377	AHR,MMP9,TGFB2	
KMT2E		enzyme			0.0377	CDC6,CDK1,E2F1	
ZDHHC2	1.866	enzyme			0.0377	CD9,TJP1,VIM	
RSF1		transcription regulator			0.0377	CFLAR,PTGS2,XIAP	
SPTAN1		other			0.0377	ITGA3,ITGA5,ITGAV	
ADAM10		peptidase			0.0377	APP,IL6ST,ITGA5	
PHLPP1	-1.555	enzyme			0.0377	IRS1,PRKCA,PRKCB	
mir-637		microrna			0.0377	AKT1,CCND1,FOXO1	
RBX1		enzyme			0.0377	BIRC5,CCNB1,CDK1	
HAS2	-1.866	enzyme			0.0377	ACTA2,HYAL2,TIMP1	

MYBL2	2.323	transcription regulator			0.0377	BIRC5,CCNB1,CDK1	
PPP2R2A		phosphatase			0.0377	CLSPN,SAT1,SLC22A4	
URI1		transcription regulator			0.0377	CCNB1,CDK1,FKBP5	
Hif1		complex			0.0377	CP,PGK1,VEGFA	
RPS6KB1		kinase	Activated	2.219	0.0397	CCND1,CFLAR,DHCR24,HMGCR,SREBF2	
EIF4E		translation regulator		1.964	0.0397	BIRC5,CCND1,CEBPB,UBE2C,XIAP	
OGT		enzyme		0.277	0.0397	BIRC5,CCND1,FOXM1,MMP2,VIM	
SMAD7		transcription regulator		-1	0.0397	BCL2L11,CFLAR,CITED2,TAGLN,XIAP	
SRA1		transcription regulator		-1.264	0.0397	CAV1,MMP9,SLC2A3,TGFB2,TMEM65	
HCAR2		g-protein coupled receptor	Inhibited	-2.236	0.0397	CCNE2,IQGAP3,KIF20A,PLK4,TRIM8	
HOXA13		transcription regulator			0.0397	BMP2,HOXB6,SHROOM3,VAPA,WNT5A	
RFX5		transcription regulator			0.0397	B2M,CD74,COL1A2,GCNT2,HLA-DMB	
SIRT1		transcription regulator		-1.03	0.0399	BIRC5,BNIP3,CCNG2,FN1,FOXO3,HMGCR,IGF1,MMP2,MMP9,NAT1,NR1D1,SCX,XPC	
TWIST1		transcription regulator		1.177	0.04	AXL,C3,COL1A1,FGFR3,FMOD,FOXO1,LMNA,MMP2,RHOC,VEGFA,YBX1	
mir-486		microrna		0.522	0.0414	CREBL2,FOXO1,RFFL,SLC4A8,TOB1,TWF1,WDFY3	
TFEB		transcription regulator		-0.57	0.0414	ARSB,ATP6V0E1,CLCN7,GNS,NAGLU,NEU1,TPP1	
PRKAA		group		-0.624	0.0417	BIRC5,CLDN1,FOXO3,VIM,XPC,YBX1	
CAV1	-2.068	transmembrane receptor		-1.698	0.0417	CAV1,HK2,SLC2A3,TFPI,TJP1,TJP2	
PIK3CA		kinase			0.0417	BIRC5,CCNB1,CCND1,CD14,FOXM1,GSR	
ATF1	1.735	transcription regulator			0.0417	FOSL1,FTH1,FUT7,PTGS2,TEAD1,YWHAZ	

AURK		group		1.069	0.0425	CMBL,CSMD3,CYFIP2,CYP26B1,FDXR,HSPA4L,PHLDB3,PSTPIP2,RRAD,SCRIB,SESN1,TCEA3,TNFRSF10A,XPC	
ANLN	2.389	other		1.069	0.0425	CMBL,CSMD3,CYFIP2,CYP26B1,FDXR,HSPA4L,PHLDB3,PSTPIP2,RRAD,SCRIB,SESN1,TCEA3,TNFRSF10A,XPC	
CBX5	1.694	transcription regulator		-0.067	0.043	ABCB1,ALDH3A1,AXIN2,BIRC5,CD68,CDC25A,CDC6,CDH17,CEACAM1,CEMIP,FKBP14,FOXQ1,HIST1H1C,HIST2H2AA3/HIST2H2AA4,HOXB6,KRT80,MAP1LC3A,OAS1,PODXL2,PRSS23,SLC2A3,SYTL2,TCEAL1,TGFBI,TM4SF1,TXNIP	
FAS		transmembrane receptor		-0.447	0.0437	AIFM1,CEBPD,CFLAR,EFNA1,FOXO3,IER3,JUND,MMP9,NUAK2,PLAU,RND1,ZFP36	
miR-483-3p (miRNAs w/seed CACUCCU)		mature microrna		-0.669	0.0437	ADIPOR2,ASH2L,BRCA1,CREBL2,DCAF6,ECHDC3,GJA1,HNRNPA0,MKI67,PSEN2,SLC7A1,TVP23B	
FGF8		growth factor		0.277	0.0468	AIF1L,CDK20,CRIP1,CYCS,DDAH2,ELL2,FBXL16,FGFR2,RTKN2,RTN4RL1,SPARC,SPP1,TNXB	
LIN28A		other		1.992	0.0517	CCND1,CDC20,CDC25A,CDK1	
PTEN		phosphatase	Inhibited	-2.913	0.0524	BCL2L11,BIRC5,CCNB1,CDC25A,EGFR,FOXO3,FTH1,GSR,MCAM,PREX1	
MMP9	-2.035	peptidase	Activated	2.2	0.0557	CCND1,SDC4,TGFB1,TJP1,VEGFA	
OLR1		transmembrane receptor	Inhibited	-2.213	0.0557	RARG,RBP1,TGFB1,TGFBR2,TGFBR3	
KLF4	2.963	transcription regulator	Inhibited	-2.626	0.0571	ALCAM,BUB1B,CCNB1,CENPE,FOXM1,ODC1,SLC4A7,SNAI2,VIM	
CD3 group		group	Activated	2.158	0.0625	ACSL4,ACSL5,CBLB,CCND3,EGR2,LPCAT4,MBOAT1,PLA2G4A,SCD,TFRC	
IGFBP5		other		1.998	0.0748	CCND1,EFNB2,IRS1,SFN	
NR4A1		ligand-dependent nuclear receptor	Activated	2.165	0.097	ACTA2,BIRC5,COL1A1,COL1A2,E2F1	
EIF2AK3		kinase		1.966	0.102	ATF4,CA9,DDIT3,VEGFA	

mir-148		microrna		1.962	0.102	ALCAM,IGF1R,MMP15,PIK3IP1		
YBX1	1.568	transcription regulator	Activated	2.4	0.107	ABCB1,ACTA2,COL1A2,EGFR,ID1,KLF4,PTGS2,TGFB1		
GATA4		transcription regulator	Activated	2.05	0.162	CDCA8,COL1A1,COL1A2,COL3A1,DES,DSCC1,ESPL1,GJA1,HES1,NFATC2,NUSAP1,TNNT2,TTN		
HMOX1		enzyme		1.929	0.167	ANGPT1,FGF1,ID1,ID3,IGF1,IL1RN,MMP9,TGFB2,THBS2,VEGFA		
CAPN3		peptidase	Activated	2	0.168	DUSP1,FTH1,GCLM,PTGS2		
SMARCB1		transcription regulator	Inhibited	-2.429	0.214	ABCB1,AURKA,CCNA2,CCND1,CDC6,E2F1,OAS1		
Gm-csf		group	Activated	2.194	0.237	BIRC5,CD14,MED14,MMP9,PTGS2,TGFB1,TRIB2,XIAP		
TRAF2		enzyme	Activated	2	0.245	AURKA,AURKB,CCNB1,CDK1		
mir-218		microrna		1.98	0.245	FZD4,MDGA1,PDGFRA,SASH1		
MED1		transcription regulator		1.942	0.256	AURKA,BOP1,CCND1,CDC6,CHAF1A,HAS2,MAD1L1,PIM1,XBP1		
NFkB (complex)		complex	Activated	2.84	0.265	ABCB1,AHR,BAD,BAK1,BMP2,C3,CCL17,CCNB1,CCND1,CDC25B,CEBPB,CFLAR,CXCL12,CXCL2,ERAP1,FOXF1,FTH1,HAS2,HDAC6,HLA-DMB,IER3,IL15,IL15RA,ITGAV,ITGB8,JUNB,LITAF,MMP2,MMP9,NFKB1,NFKBIZ,NOS1,NUMBL,PLAU,PTGS2,RFTN1,SDC4,SENP1,SENP2,SNAI2,TACR1,TGFB1,TRAF1,TRIB3,XIAP		
BRD7		transcription regulator		1.98	0.285	KRT8,NDRG1,RAD51,VCAN		
RAE1		other	Inhibited	-2	0.285	EIF1AD,FRAT2,ING1,SIAH1		
MTOR		kinase		1.93	0.287	BNIP3,CASP1,CCND1,CFLAR,CXCL2,DDIT4,EGFR,PHLPP1,SNAI2,SREBF2,UBE2C		
YY1		transcription regulator		1.972	0.308	BRCA1,HAS2,NR3C1,POLB,STAR,VEGFA		
LONP1		peptidase	Inhibited	-2.121	0.331	ACADS,ACAT1,EARS2,ETFB,GPT2,MARS,MRPL18,MTHFD2,OXCT1,PTGS2,SARS,SMDT1,TPM3		
RBM5		other		-1.795	0.331	ATP6V0C,GPER1,HSP90AA1,ITPA,PIM1,PLK4,TRAF1,UBA1,XIAP		
ETS1		transcription regulator		-1.96	0.401	ARHGDIB,CASP1,CCDC90B,CDK11A,CHUK,FN1,ITGA2B,MMP9,MRPL9,PLAU,SCAMP1,SZRD1,TGFA,TGFB2		

ADIPOQ		other		1.984	0.427	ADIPOR2,CCND1,FN1,PTGS2,TIMP1	
IL12 (complex)		complex	Activated	2.785	0.455	GATA3,IL18RAP,ITGB2,MMP2,MMP9,PLAU,TGFBR2,TIMP1	
IL1B		cytokine	Activated	3.042	0.462	A4GALT,AMPD3,C3,CAT,CEBPB,COL1A1,CXCL2,DNMT1,DUSP1,FGFR2,FLT1,IGF1,IL15,IL18,IL1RN,IL6R,ITGB8,LBP,MMP9,NCOA2,NFKB1,NFKBIZ,NR4A2,NRP1,PLA2G4A,PTGS2,SEMA3A,TACR1,TGFB1,TLR3,TNFAIP6,TOB1,UBD,UGCG,VEGFA	
miR-22-3p (miRNAs w/seed AGCUGCC)		mature microrna		-1.98	0.531	ARRB1,CHD7,FRAT2,MTHFD2	
AHR	6.109	ligand-dependent nuclear receptor	Activated	2.415	0.534	CCND1,COL1A1,COL3A1,PTGS2,TGFB2,VEGFA	
Fcer1		complex		1.987	0.568	IL1RN,INHBA,NFKB1,TNFRSF9	
